# Vanadium-Containing Ionic Liquids Derived from Complexes of Modified Edta as Catalysts of Epoxy-Anhydride Ring-Opening Copolymerization

**DOI:** 10.1021/acs.inorgchem.4c01663

**Published:** 2024-08-29

**Authors:** Lukáš Hanzl, Jaromír Vinklárek, Miroslava Litecká, Marwa Rebei, Hynek Beneš, Aleš Eisner, Tomáš Mikysek, Anna Krejčová, Jan Honzíček

**Affiliations:** † Department of General and Inorganic Chemistry, Faculty of Chemical Technology, University of Pardubice, Studentská 573, Pardubice 532 10, Czech Republic; ‡ Department of Materials Chemistry, Institute of Inorganic Chemistry of the CAS, Husinec-R̆ež 1001, R̆ež 25068, Czech Republic; § Institute of Macromolecular Chemistry, Czech Academy of Sciences, Heyrovského nám. 2, Prague 6 162 00, Czech Republic; ∥ Department of Analytical Chemistry, Faculty of Chemical Technology, University of Pardubice, Studentská 573, Pardubice 532 10, Czech Republic; ⊥ Institute of Environmental and Chemical Engineering, Faculty of Chemical Technology, University of Pardubice, Studentská 573, Pardubice 532 10, Czech Republic; # Institute of Chemistry and Technology of Macromolecular Materials, Faculty of Chemical Technology, University of Pardubice, Studentská 573, Pardubice 532 10, Czech Republic

## Abstract

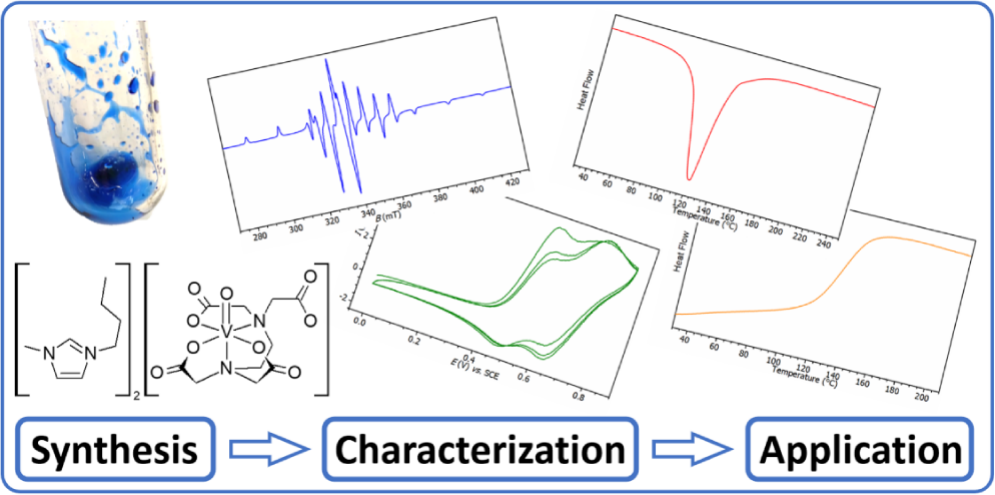

A new type of vanadium-containing ionic liquids (ILs) was synthesized by cation exchange from barium salts of oxidovanadium(IV) complexes stabilized by edta and its congeners (dcta, oedta, and heedta) serving as pentadentate ligands. All starting barium salts and several magnesium and cesium salts, serving as models for the cation exchange, were structurally characterized by single-crystal XRD analysis. The synthesized ILs consisting of organic cations (Bu_4_N^+^, Bmim^+^, and Bu_4_P^+^) and complex anions ([VO(edta)]^2–^, [VO(dcta)]^2–^, [VO(oedta)]^−^, and [VO(heedta)]^−^) were characterized by analytical and spectroscopic methods including EPR spectroscopy and cyclic voltammetry. Then, ILs were tested as catalysts for the ring-opening copolymerization of epoxy resin with cyclic anhydride showing significant catalytic activity, which led to production of highly cross-linked glassy thermosets. A detailed isothermal DSC kinetic study was performed for the most promising IL showing that the progress of cross-linking can be successfully fitted by the Kamal–Sourour model. Based on the DSC and NIR results, the initiation mechanism of the cross-linking in the presence of vanadium-containing IL was suggested. IL had ability to activate a rapid hydrolysis of anhydride cycle and the formed carboxyl groups initiated a polyesterification. In parallel, the role of imidazolium cation of IL for the initiation of chain-growth anionic copolymerization is also discussed.

## Abstract 2

Vanadium-containing ionic liquids were synthesized and characterized. They serve as catalysts for the ring-opening copolymerization.

## Introduction

Ionic liquids (ILs) are defined as salts with a melting point lower than 100 °C. They show unique properties such as low volatility, low flammability, high thermal and chemical stability,^1^ which predispose them to be promising alternatives to conventional solvents,^2^ although their low environmental impact and sustainability have recently been partially questioned.^3−5^ The wide diversity of ILs is due to their modular nature. They usually consist of organic cations and organic or inorganic anions.

Transition-metal-containing ILs (TM-IL) have been extensively studied since a strong magnetic field response was discovered for paramagnetic iron-containing IL, [Bmim][FeCl_4_], in 2004.^6^ Magnetic TM-ILs have been thoroughly scrutinized, leading to their use in the fields of analytical microextraction,^7−9^ and in sensing applications.^10^ TM-ILs are usually classified as task-specific ILs, as the role of the ionic liquid goes beyond that of a solvent.^11^ It is not surprising that the transition metals present in the cation or anion catalyze various reactions used for organic synthesis^11−13^ and industrially relevant processes.^14−18^ Furthermore, TM-ILs were found to be suitable precursors for electrodeposition of transition metals^19,20^ and luminescent materials.^21−23^

**Scheme 1:**
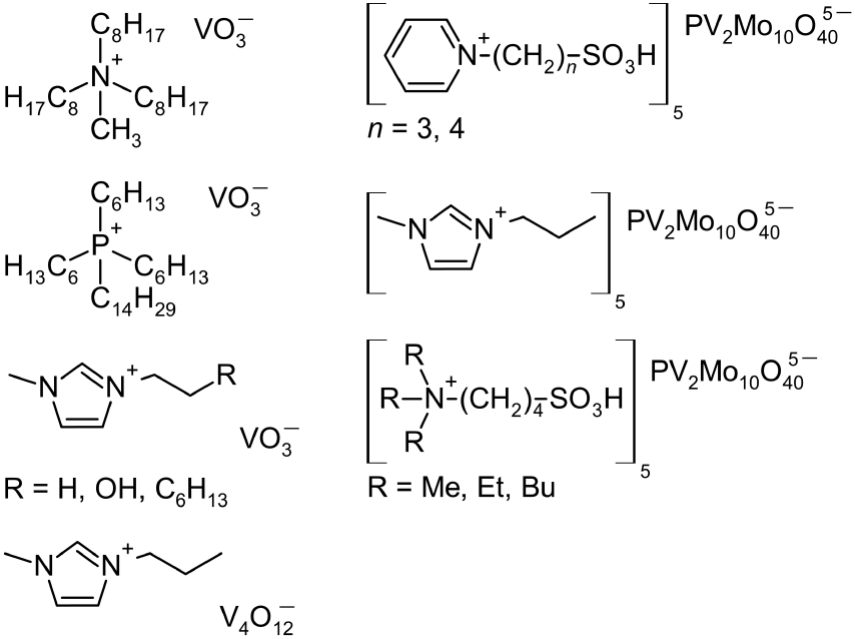
Examples of Vanadium-Containing ILs Reported Elsewhere^24−26^

Although ILs containing first-row transition metals have been deeply scrutinized, only a few studies deal with vanadium. They involve vanadium in the form of vanadate and molybdovanadate anions given in Scheme 1.^24−26^ ILs containing metavanadate anion were studied for their electrochromic behavior.^24^ Molybdovanadate-based ILs serve as cocatalysts of Heck coupling reaction.^26^

Due to their high thermal and chemical stability, liquid state and compatibility/miscibility with epoxy monomers, ILs are considered as promising initiators/catalysts of epoxy ring opening.^27,28^ Imidazolium and phosphonium ILs, which enable the preparation of epoxy networks with excellent mechanical properties, appear particularly promising.^29−31^ Unfortunately, a relatively high amount of IL (>5 wt %) is usually required. Recently, TM-ILs have also been recognized as very efficient catalysts/initiators for epoxy polymerizations.^31,32^ Rebei et al. demonstrated TM-IL (based on Co, Zn and Fe) as perspective catalysts of the epoxy-anhydride ring-opening copolymerization^33^ in applications requiring a moderate exotherm profile (typically curing of thick composites) and a low cure onset temperature (using mold materials sensitive to temperature). The advantage of TM-IL is their sufficient catalytic activity at a low content (0.4–2.8 wt %). Moreover, Kleij reported that vanadium(V) complexes derived from aminotriphenolate ligands were highly active catalysts for the coupling of various terminal and internal epoxides with carbon dioxide to provide a series of substituted organic carbonates in good yields.^34^ The reported coordination of the epoxide to the vanadium center followed by oxirane ring opening indicated the possibility of using vanadium-containing compounds as initiators/catalysts of epoxy-anhydride copolymerizations.

This study focuses on the investigation of catalytically active vanadium-containing ILs mainly due to the low overall toxicity of vanadium compounds,^35^ their high thermal stability, and sufficient commercially exploitable reserves of vanadium.^36^ As proof of concept, we set out to synthesize and characterize a series of anionic oxidovanadium(IV) complexes stabilized ethylenediaminetetraacete (edta) and its congeners 1,2-diaminocyclohexanetetraacetate (dcta), *N*-octylethylenediaminetriacetate (oedta), and hydroxyethylethylenediaminetriacetate (heedta). As counterions, tetrabutylammonium (Bu_4_N), 1-butyl-3-methylimidazolium (Bmim) and tetrabutylphosphonium (Bu_4_P) cations were selected. The ability of vanadium-containing ILs to perform as homogeneous catalysts will be demonstrated in the ring-opening copolymerization of epoxy resin with cyclic anhydride. Note that vanadium complexes of edta exhibit high stability constants^37^ and are expected to resist harsh conditions during the curing process.

## Results and Discussion

### Synthesis of Edta Complexes

**Scheme 2:**
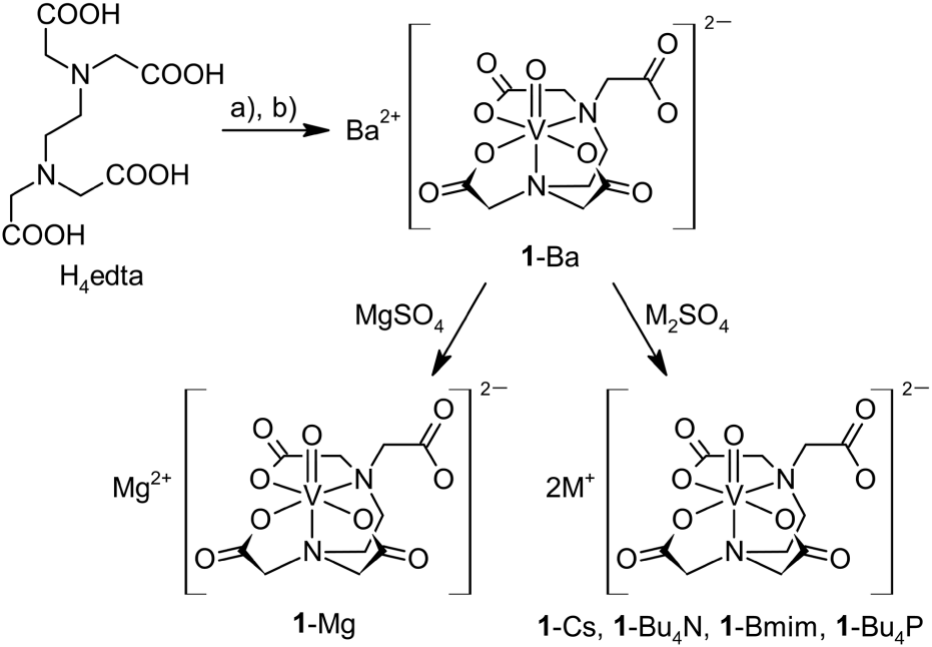
Synthesis of Oxidovanadium(IV) Complexes Stabilized by Edta Reagents: a) VOSO_4_·3H_2_O/H_2_O, b) BaCO_3_.

Anionic oxidovanadium(IV) complex Ba[VO(edta)] (**1**-Ba) was prepared using a modified literature procedure starting from an aqueous solution of oxidovanadium(IV) sulfate and H_4_edta.^38^ The appearing acidic solution was neutralized by barium carbonate, which allows the removal of sulfate anions (Scheme 2). We note that barium carbonate can be used in excess because it is insoluble in neutral solutions and was easily removed by filtration together with the produced barium sulfate. Pure **1**-Ba·6H_2_O was obtained after evaporation of water at elevated temperature. In an aqueous solution, the barium cation of **1**-Ba can be easily exchanged by treating it with an equivalent of the appropriate sulfate, as verified on magnesium (**1**-Mg) and cesium (**1**-Cs) salts. The formed barium sulfate was filtered off, and products of cation exchange were isolated by solvent evaporation. Then, this protocol was successfully used for the preparation of the ionic liquids **1**-NBu_4_, **1**-Bmim, and **1**-PBu_4_ (Scheme 2). As a source of organic cations, freshly prepared sulfates (Bu_4_N)_2_SO_4_, (Bmim)_2_SO_4_, (Bu_4_P)_2_SO_4_ were used.

**Figure 1:**
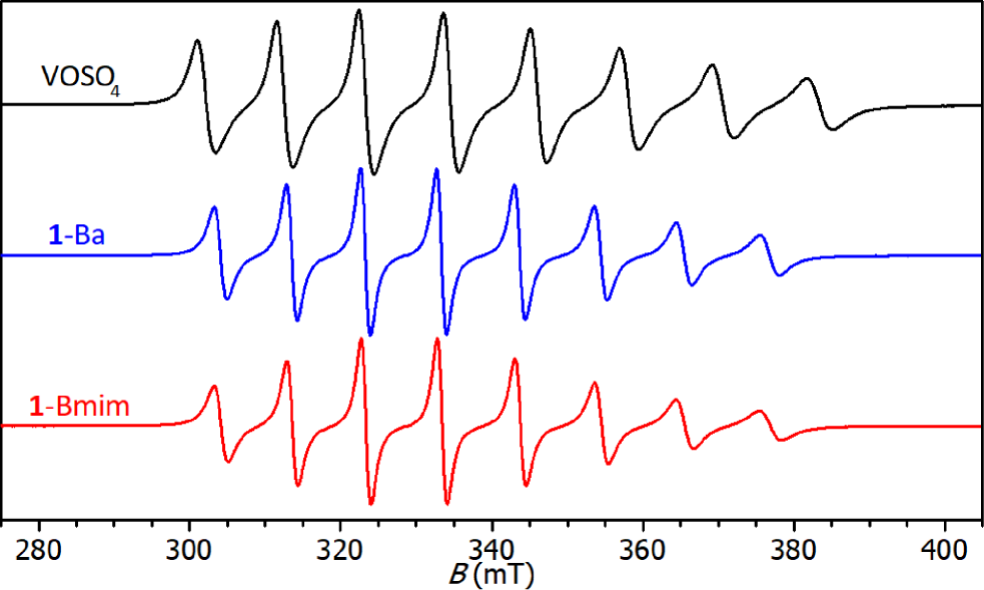
Isotropic EPR spectra of aqueous solutions of VOSO_4_ (top), **1**-Ba (middle) and **1**-Bmim (bottom); ν = 9.4 GHz.

The synthesis of **1-Ba** in aqueous solution was followed by EPR spectroscopy. The starting aqua complex [VO(OH_2_)_5_]SO_4_ gives a typical eight-line spectrum due to the interaction of the unpaired electron with the ^51^V nucleus (*I* = ^7^/_2_, 99.8%), see Figure 1. Coordination of edta leads to a considerable decrease in the isotropic hyperfine coupling constant |*A*_iso_| from 11.60 to 10.39 mT, owing to a larger delocalization of the spin density on the chelating ligand. The following exchange of Ba^2+^ ions has only a negligible effect on the isotropic EPR parameters, as it proceeds in the outer coordination sphere of vanadium.

Complexes **1**-Ba, **1**-Mg, **1**-Cs, **1**-NBu_4_, **1**-Bmim, and **1**-PBu_4_ were characterized by electrospray ionization mass spectrometry (ESI-MS). In the negative-ion mode, they give an intense peak at *m*/*z* of 356, assigned to protonated species [VO(Hedta)]^−^. The compounds **1**-Ba, **1**-Mg, **1**-Cs, and **1**-PBu_4_ further show the molecular peak of dianion [VO(edta)]^2–^ (*m*/*z* = 177.5) and its adduct with the water molecule [VO(edta)(OH_2_)]^2–^ at (*m*/*z* = 186.5). Adducts of [VO(edta)]^2–^ with monocations were observed in the case of **1**-Cs and ionic liquids **1**-NBu_4_, and **1**-PBu_4_ at 488, 597 and 614, respectively. The peaks of the heavy cation Cs^+^ and the large cations [NBu_4_]^+^, [Bmim]^+^ and [PBu_4_]^+^ were observed in positive ion mode for a given compound. In the case of **1**-Cs, the peak at 754 was assigned to the adduct of the complex anion with three cesium ions [Cs_3_{VO(edta)}]^+^.

The vanadium complexes bearing organic cations appear as blue solids (**1**-NBu_4_ and **1**-PBu_4_) or highly hygroscopic viscous liquids (**1**-Bmim). Elemental analysis of the samples revealed water absorption during the necessary handling in the air atmosphere. Therefore, thermogravimetric analysis (TGA) was utilized to quantify the residual water content in our samples. The formation of true vanadium-containing ILs was confirmed by differential scanning calorimetry (DSC). The second heating run, performed after complete drying at 200 °C, provided low *T*_g_ values consistent with the definition of ILs. Compounds **1**-NBu_4_ and **1**-PBu_4_ can be classified as room-temperature ILs.

The crystal structures of **1**-Ba·6H_2_O, **1**-Mg·9H_2_O·0.5(dioxane) and **1**-Cs·2H_2_O were determined by XRD analysis. In these compounds, the coordination sphere of the vanadium atom forms a distorted octahedron, where edta serves as a pentadentate *N,N,O,O,O*-chelating ligand. The nitrogen donor atoms of edta (N1 and N2) are not equivalent. N1 stays *trans* relative to the vanadyl oxygen atom and both acetate groups neighboring N1 are coordinated to V. The second nitrogen donor atom (N2) remains *cis* relative to vanadyl oxygen (O1), and only one of the neighboring acetate groups is bonded to the VO moiety. Note that the bond V–N1 is significantly longer [2.286(1)–2.308(3) Å] than V–N2 [2.141(1)–2.172(4) Å] due to the *trans*-effect of vanadyl oxygen.^39^

**Figure 2:**
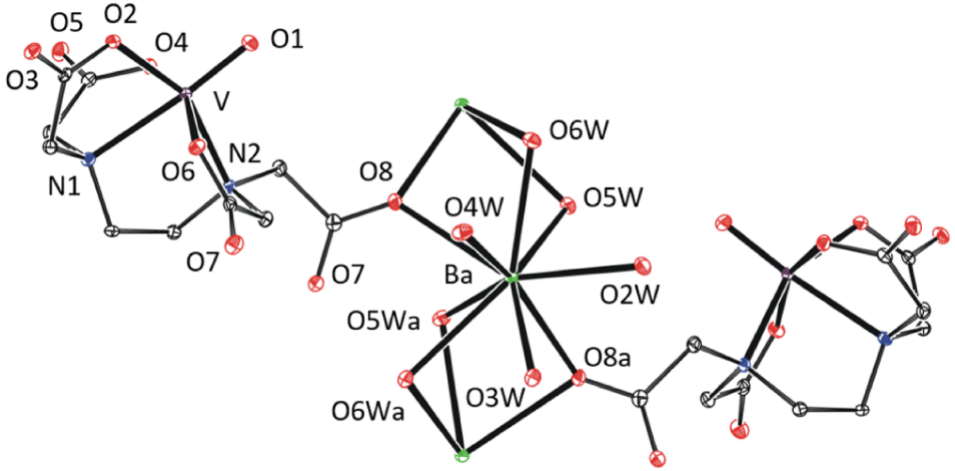
Coordination sphere of barium(II) in the XRD structure of **1**-Ba·6H_2_O. Thermal ellipsoids are set to 30% probability. Hydrogen atoms and free water molecules are omitted for clarity.

In **1**-Ba·6H_2_O, barium ions are nonacoordinated by three terminal aqua ligands, the two bridging carboxylate groups and four bridging aqua ligands forming zigzag chains. Each bridge between two barium atoms consists of one carboxylate of the edta ligand and two bridging aqua ligands (Figure 2). Note that uncoordinated water molecules (one per one Ba^2+^) stay in the channel between the zigzag chains and are stabilized by three hydrogen bonds.

In crystal lattice of **1**-Mg·9H_2_O·0.5(dioxane), no direct interaction is observed between Mg^2+^ and the oxygen atoms of edta, as the Mg^2+^ is fully solvated by six aqua ligands (Figure S1), which is in line with the previously reported crystal structure of similar solvate **1**-Mg·9.5H_2_O.^40^ The structure of **1**-Cs·2H_2_O is burdened by a positional disorder on the cesium atoms. Its molecular structure is shown in Figure S2. It should be noted that two related structures of alkali metal salts have been reported elsewhere. In both cases, vanadium(IV) bears a protonated edta ligand, Na[VO(Hedta)]·4H_2_O,^41^ K[VO(Hedta)]·3H_2_O.^42^

### Synthesis of Complexes from Edta Congeners

**Scheme 3:**
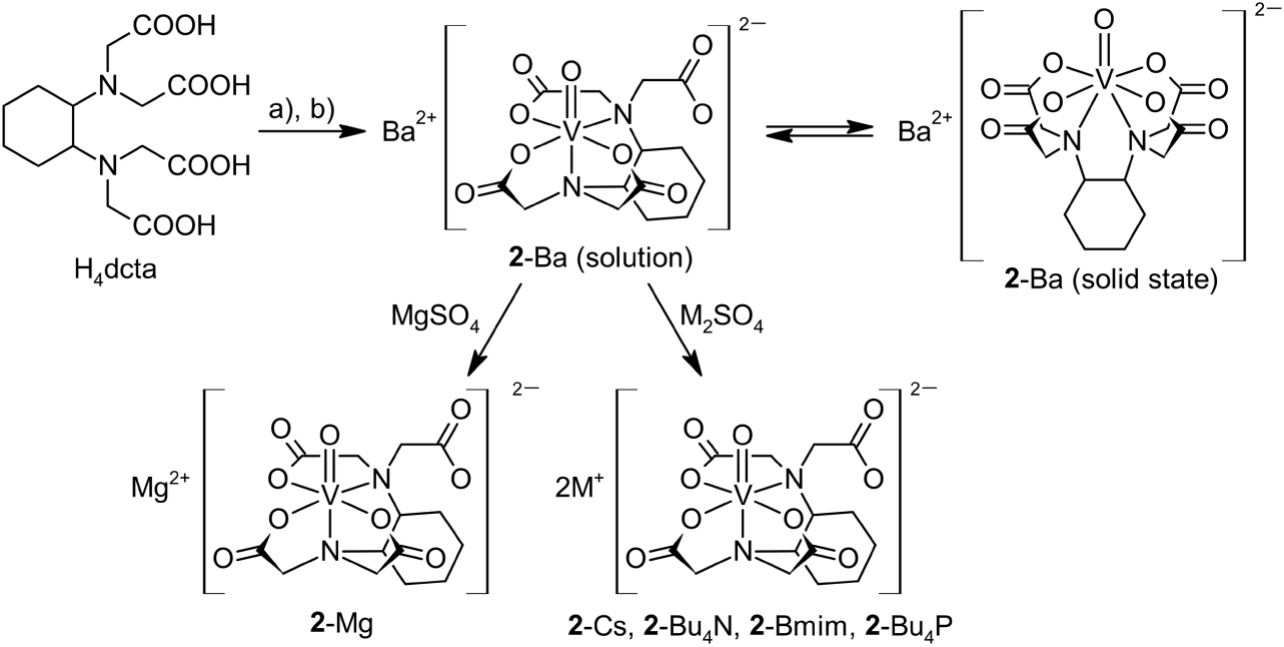
Synthesis of Oxidovanadium(IV) Complexes Stabilized by dcta Reagents: a) VOSO_4_·3H_2_O/H_2_O, b) BaCO_3_.

**Scheme 4:**
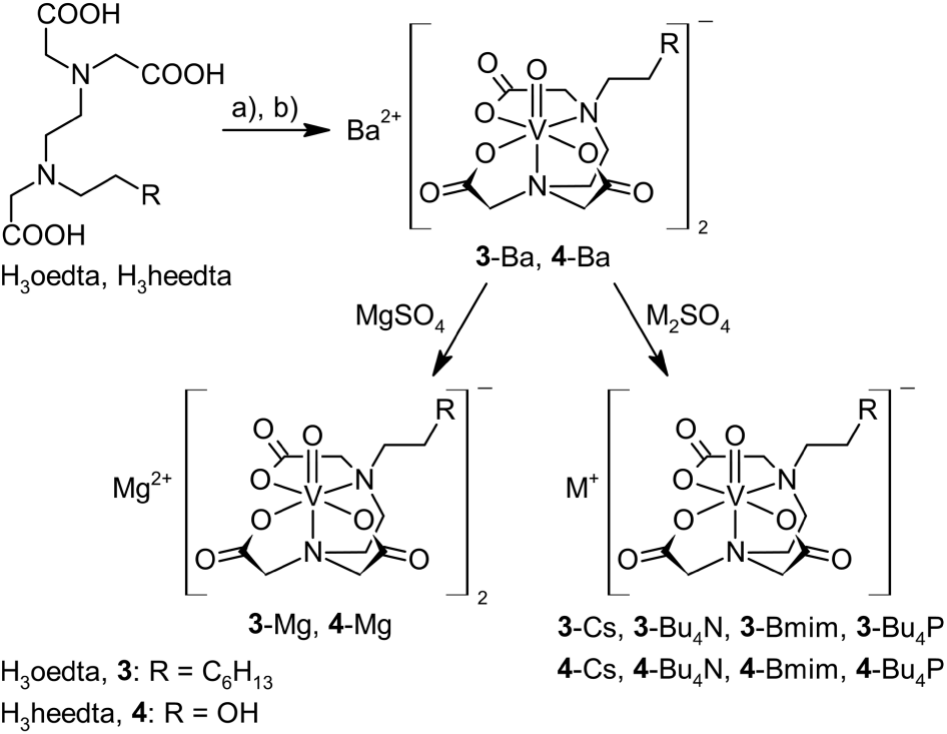
Synthesis of Oxidovanadium(IV) Complexes Stabilized by Oedta, and Heedta Reagents: a) VOSO_4_·3H_2_O/H_2_O, b) BaCO_3_.

A series of complexes bearing dcta, oedta, and heedta were prepared using modified protocols shown in Schemes 3 and 4 and their aqueous solutions characterized by EPR spectroscopy and mass spectrometry. The isotropic EPR spectra of aqueous solutions show a pattern typical for single paramagnetic species. The determined values of the parameters *g*_iso_ and |*A*_iso_|, given in Table S1, are close to those obtained for the edta complexes, revealing a very similar coordination sphere of vanadium(IV).

Positive-ion spectra show peaks of heavy Cs^+^ and large cations [NBu_4_]^+^, [Bmim]^+^ and [PBu_4_]^+^. In the case of **3**-Bmim, the adduct [(Bmim)_2_{VO(oedta)}]^+^, observed at *m*/*z* of 688, suggests a weak interaction of imidazolium with the nonpolar octyl tail. The appearance of several adducts for the cesium salts **2**-Cs ([Cs_2_{VO(Hdcta)}]^+^, [Cs_3_{VO(dcta)}]^+^), **3**-Cs ([Cs{VO(Hoedta)}]^+^, [Cs_2_{VO(oedta)}]^+^) and **4**-Cs ([Cs_2_{VO(heedta)}]^+^), imply appearance of a weak interaction between the periphery of the complex anions and cesium(1+), which is in line with their soft Pearson basicity and complex networks of short contacts documented by XRD analyzes.

Negative-ion spectra of the dcta complexes show a pattern similar to that of the edta analogues with peaks at *m*/*z* = 205, 214, and 410 assigned to [VO(dcta)]^2–^, [VO(edta)(OH_2_)]^2–^, and [VO(Hedta)]^−^, respectively. In the case of **2**-Ba and **2**-Mg, additional peaks were detected at *m*/*z* of 478 and 421, respectively. They were assigned to the dianionic adducts [Ba{VO(dcta)}_2_]^2–^ and [Mg{VO(dcta)}_2_]^2–^, implying a stronger interaction between the dcta complexes and alkali-earth metals than in the case of the edta analogues. Monoanionic complexes bearing oedta and heedta exhibit molecular peaks [VO(oedta)]^−^ and [VO(heedta)]^−^ at *m*/*z* of 410 and 342, respectively.

The residual water content was determined by TGA analysis for vanadium complexes containing organic cations selected for the testing of catalytic activity (Table 1). The compounds have a true IL character, as confirmed by DSC analysis. We note that compounds **2**-Bmim, **3**-Bu_4_P, and **4**-Bmim can be classified as room-temperature ILs.

**Table 1:** Properties of Vanadium-Based ILs

	appearance^a^	water content (wt %)^b^	*T*_g_ (°C)^c^
**1**-Bu_4_N	blue solid	2.5	36
**1**-Bmim	blue viscous liquid, hygroscopic	7.6	17
**1**-Bu_4_P	blue solid	2.0	19
**2**-Bmim	blue viscous liquid, hygroscopic	7.4	18
**3**-Bu_4_N	blue solid	2.7	43
**3**-Bmim	blue viscous liquid, hygroscopic	6.2	24
**3**-Bu_4_P	blue viscous liquid, hygroscopic	4.6	4
**4**-Bmim	blue viscous liquid, hygroscopic	5.7	15

a Neat samples.

b Determined by TGA analysis on neat samples.

c Determined by DSC analysis (second run after evaporation of residual water).

**Figure 3:**
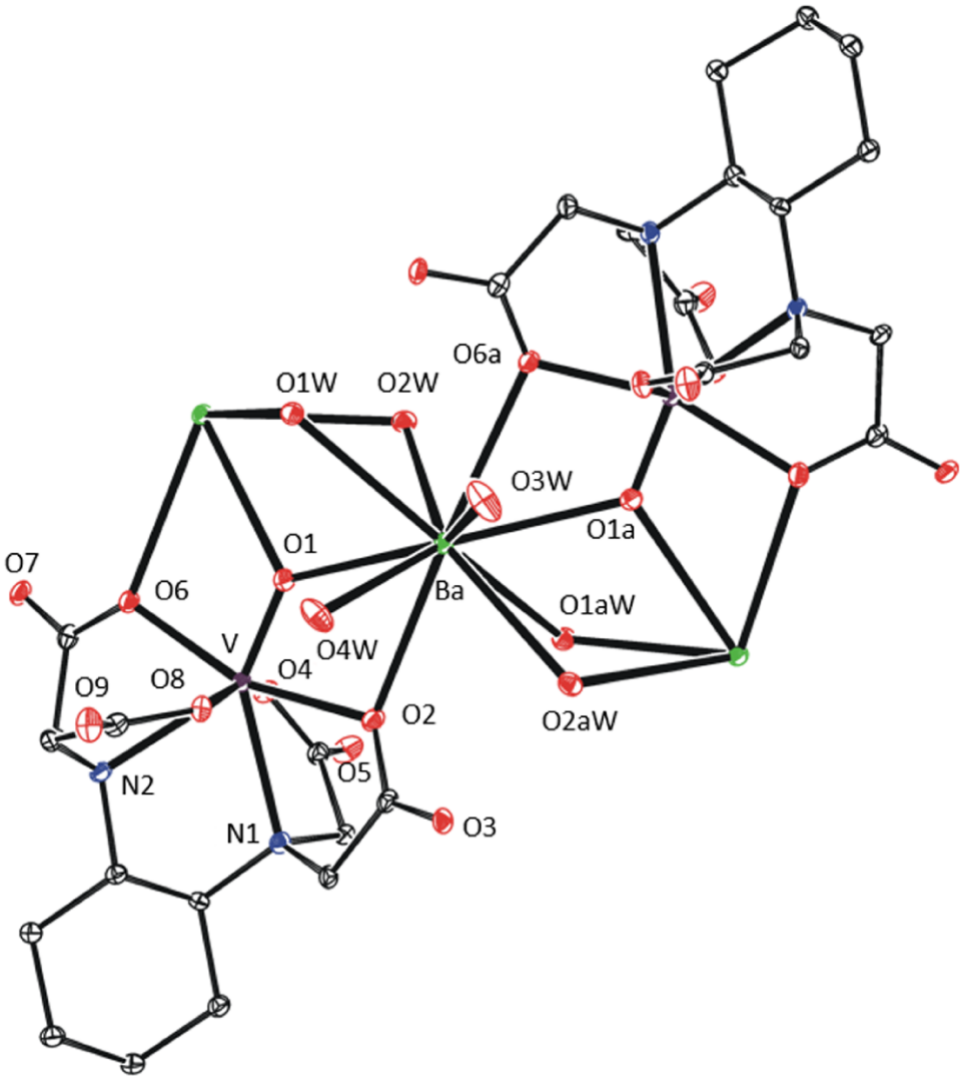
XRD structure of **2**-Ba·6H_2_O. Thermal ellipsoids are set to 30% probability. Hydrogen atoms and free water molecules are omitted for clarity.

The solid-state structure of **2**-Ba·6H_2_O represents a very unusual example of an oxidovanadium(IV) compound with a heptacoordinated central metal, where dcta serves as a hexadentate ligand (Figure 3). Four carboxylate groups of the dcta ligand are close to the equatorial plane perpendicular to the V=O bond while the nitrogen atoms of the dcta stay opposite to vanadyl oxygen with the O1–V–N1 and O1–V–N2 bond angels 144.8(1) Å and 144.3(1) Å, respectively. This arrangement is stabilized by interactions of the barium(II) ion with the vanadyl oxygen atom (O1) and with two acetate groups of dcta but leads to a significant prolongation of the V–N bonds [V–N1 = 2.429(4) Å, V–N2 = 2.413(3) Å]. The barium ions in **2**-Ba·6H_2_O are decacoordinated by two terminal aqua ligands, four bridging aqua ligands, two vanadyl oxygen and two carboxylate oxygen atoms, forming zigzag chains. Each bridge between two barium atoms consists of one vanadyl oxygen atom (V1) and two bridging aqua ligands (Figure 3). We note that the high coordination number of vanadium is not preserved in solution as proposed on the basis of the EPR measurements mentioned above.

The pentadentate coordination mode of the oedta and heedta ligands is documented on the XRD structures of **3**-Ba·7H_2_O·2^i^PrOH, **4**-Ba·3H_2_O, **4**-Mg·8H_2_O, and **4**-Cs·H_2_O. In these crystal structures, the coordination sphere of the vanadium atom is very similar to that of the edta complexes, as documented by the geometric parameters given in Tables S3 and S4. This observation is consistent with the previously reported crystal structure of **4**-K·H_2_O.^43^

**Figure 4:**
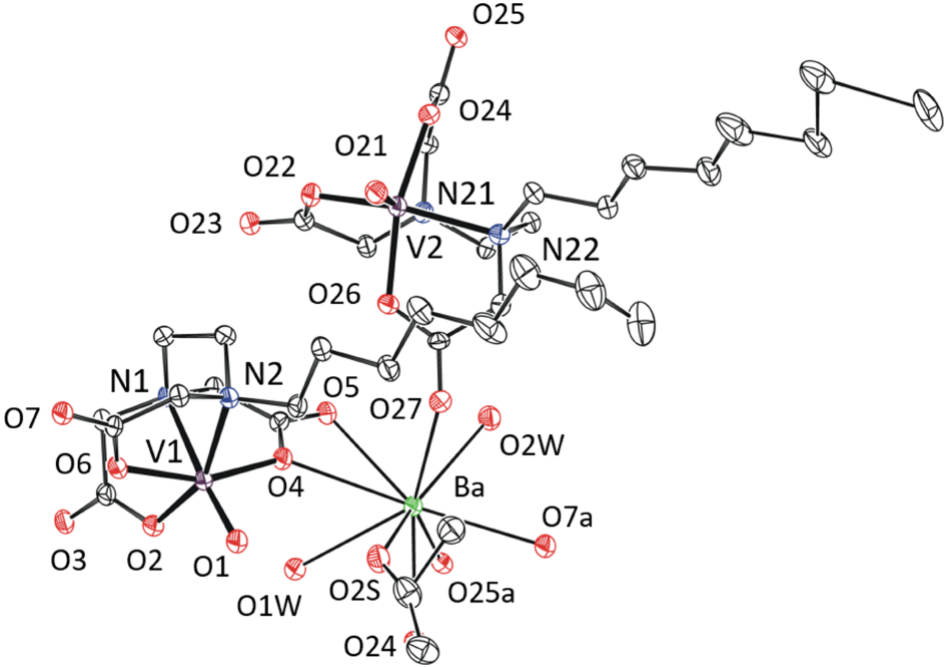
Coordination sphere of barium(II) cation in the XRD structure of **3**-Ba·7H_2_O·2^i^PrOH. Thermal ellipsoids are set to 30% probability. Hydrogen atoms and free solvent molecules are omitted for clarity.

In **3**-Ba·7H_2_O·2^i^PrOH, barium atom is nonacoordinated by two terminal aqua ligands, one ^i^PrOH molecule and six oxygen atoms of the oedta ligand (Figure 4). Two oedta carboxylates, in the barium coordination sphere, are κ^1^-coordinated through C=O oxygen, while the other two are κ^2^-coordinated. Note that disordered solvent molecules are placed in a cavity between the alkyl tails.

**Figure 5:**
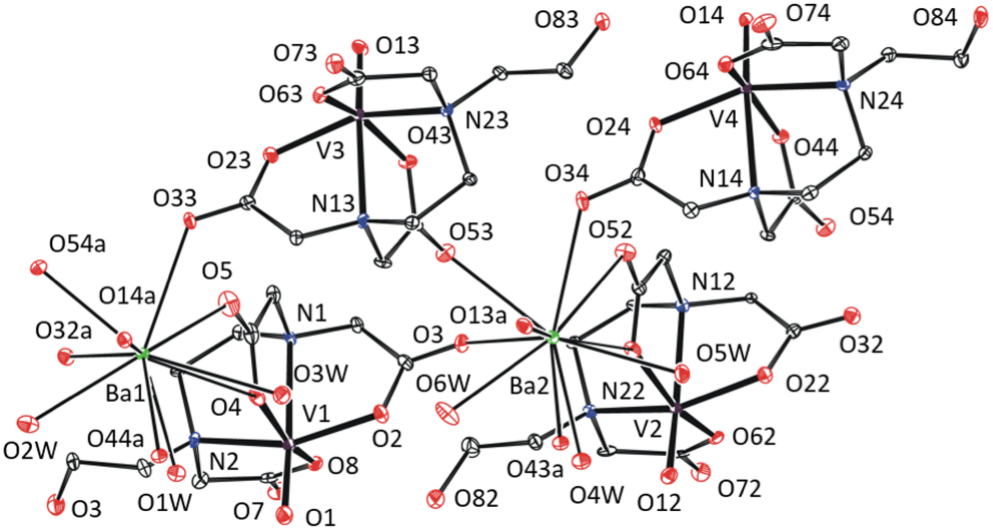
Coordination sphere of barium(II) cations in the XRD structure of **4**-Ba·3H_2_O. Thermal ellipsoids are set to 30% probability. Hydrogen atoms are omitted for clarity.

The crystal structure of **4**-Ba·3H_2_O contains two crystallographically independent barium atoms, both positionally disordered. Nevertheless, it is clear that both contain in the coordination sphere three terminal aqua ligands, three carboxylates bonded through C=O oxygen, one carboxylate bonded through C–O oxygen, one κ^2^-coordinated carboxylate and one vanadyl oxygen atom (Figure 5). Note that the positions of hydroxyethyl tails in the crystal lattice are stabilized by hydrogen bonds.

**Figure 6:**
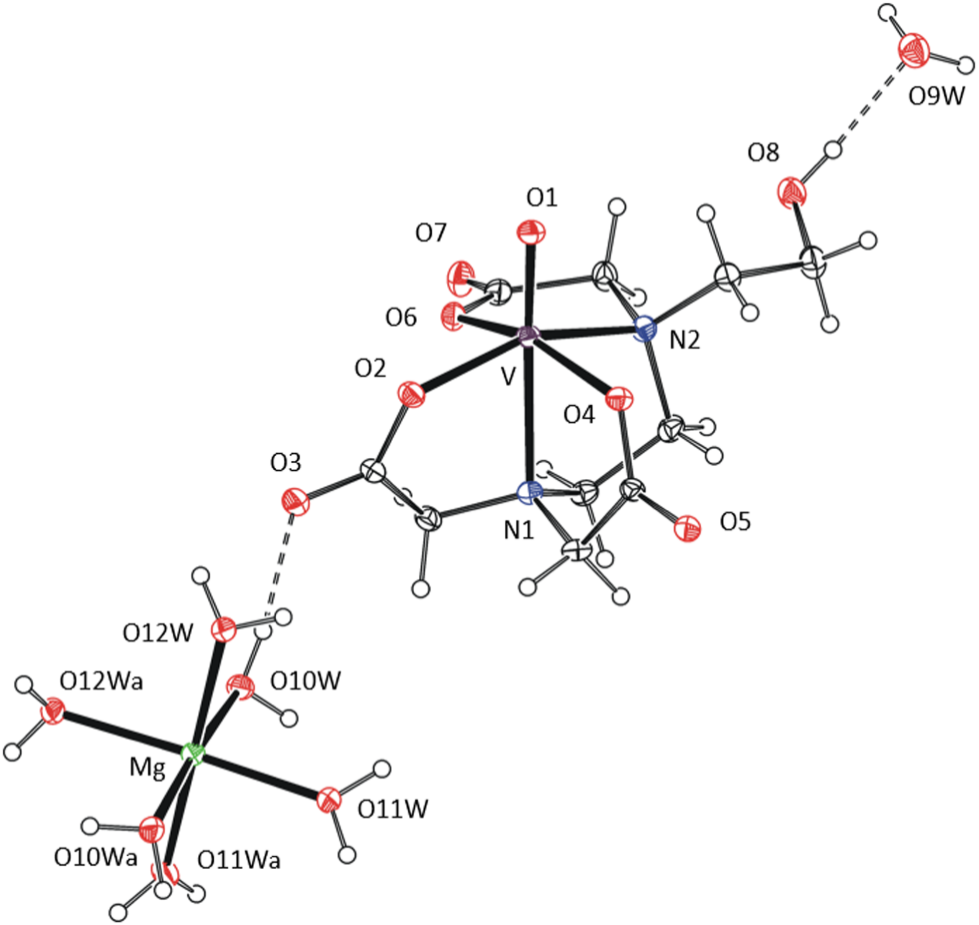
XRD structure of **4**-Mg·8H_2_O. Thermal ellipsoids are set to 30% probability.

In **4**-Mg·8H_2_O, magnesium is fully solvated by six aqua ligands (Figure 6), which fits the Pearson theory of hard and soft acids and bases, since magnesium, as a “hard acid”, prefers aqua ligands as “harder bases” than atoms complex periphery. The “softer” character of Cs^+^ leads to the formation of the monohydrate **4**-Cs·H_2_O. The decacoordinated cesium atom is surrounded by three bridging aqua ligands, bridging carboxylates, and vanadyl oxygen (Figure S3).

### EPR Studies on Ionic Liquids

**Figure 7:**
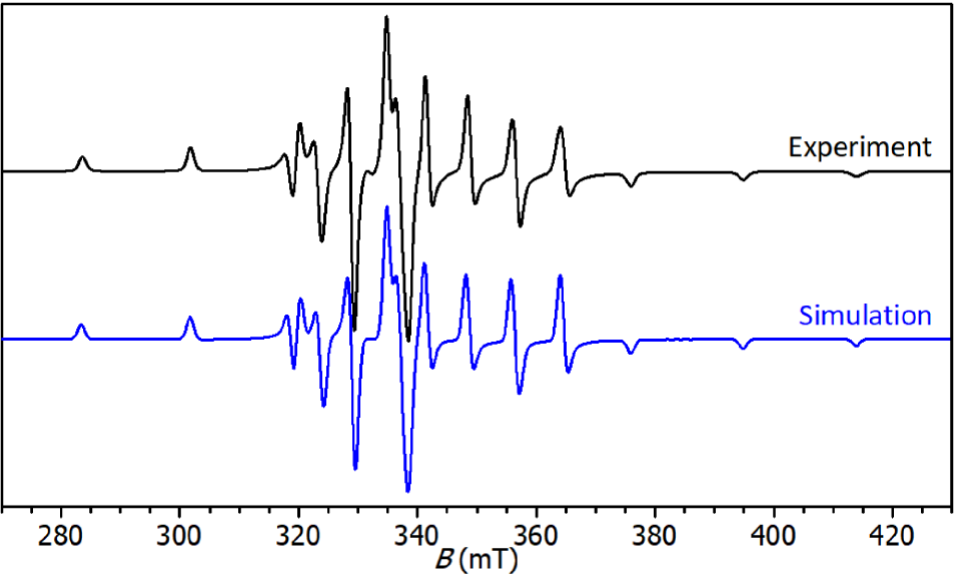
Anisotropic EPR spectrum of **3**-Bu_4_N in frozen methanol/DMSO mixture measured at −150 °C (ν = 9.4914 GHz). Experimental spectrum (top) and its computer simulation (bottom).

The improved solubility of the synthesized ionic liquids in organic solvents allows one to acquire anisotropic EPR spectra of frozen methanol mixtures (Figure 7) and obtain more detailed knowledge of the coordination sphere of the central metal than from the isotropic spectra of fluid solutions. The anisotropic spectra of the ionic liquids, presented here, are axially symmetric with *A*_∥_ > *A*_⊥_ and *g*_∥_ < *g*_⊥_ (Table S2). It implies the *C*_4_ symmetry of the SOMO orbital, which is in line with the pentadentate coordination mode of the edta-based ligands proposed in Schemes 2–4. Frozen solutions of the edta, oedta and heedta complexes show virtually the same values of *A*-tensors (*A*_∥_ ≈ 18.62 mT, *A*_⊥_ ≈ 6.49 mT) and *g*-tensors (*g*_∥_ ≈ 1.944, *g*_⊥_ ≈ 1.978), which proves a negligible effect of the pendant substituent and counterion on the SOMO orbital of the complexes. The slightly lower value of *A*_∥_ (∼18.50 mT), observed for the dcta complexes (**2**-Bu_4_N, **2**-Bu_4_P and **2**-Bmim), is attributed to the more rigid structure of the polydentate ligand that constrains the N–V–N bond angle at a lower value. Note that this variation of the *A*_∥_ parameter was not evidenced previously for complexes of edta and dcta prepared *in situ*, probably owing to broader lines of spectra measured in water/DMSO mixtures.^44^ We further note the lower *A*_∥_ value is not consistent with the higher coordination number observed in the crystal structure of **2**-Ba and further proves the stability of the pentadentate coordination mode of the dcta ligand in oxidovanadium(IV) compounds.

### Electrochemistry on Vanadium-Containing ILs

**Figure 8:**
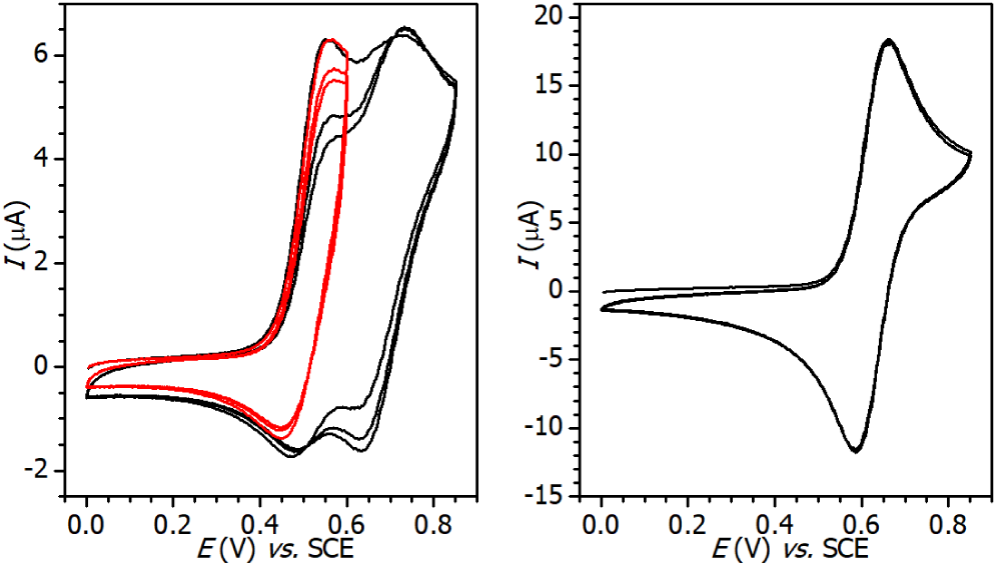
Representative CV curves of oxidation of **1**-Bmim (left) and **2**-Bmim (right) at glassy carbon electrode in MeCN containing 0.1 M Bu_4_NPF_6_; *v* = 100 mV·s^–1^.

The electrochemical behavior of **1**-Bmim, **2**-Bmim, **3**-Bmim, and **4**-Bmim was studied by cyclic voltammetry (CV) at glassy carbon electrode in acetonitrile containing 0.1 M Bu_4_NPF_6_ as the supporting electrolyte. All studied compounds undergo one or two oxidation processes within the potential window. The acquired electrochemical data is summarized in Table 2. Representative voltammograms of **1**-Bmim and **3**-Bmim are shown in Figure 8, the whole series is available in Figures S4–S7. Note that a similar complex of edta, Na[VO(Hedta)]·4H_2_O, has already been examined by cyclic voltammetry but without clarifying the relationship with the complex structure.^41^

**Table 2:** Electrochemical Data of the Studied Oxovanadium(IV) ILs

compound	*E*^oF^ (ox) (V)^a^	*E*^oF^ (V^V^/V^IV^) (V)^a^
**1**-Bmim	0.51	0.68
**2**-Bmim	0.44^b^	0.67
**3**-Bmim		0.63
**4**-Bmim		0.64

a *E*^oF^ = (*E*_p,c_ + *E*_p,a_)/2, for which *E*_p,c_ and *E*_p,a_ correspond to the cathodic and anodic peak potentials, respectively. All potentials vs SCE were obtained by CV.

b The anodic peak potential of the electrochemically irreversible process.

The main reversible one-electron and diffusion-controlled oxidation process ranges from 0.63 to 0.68 V within the series. It can be ascribed to the oxidation of vanadium(IV) to vanadium(V), which was proved experimentally by EPR spectroelectrochemistry. At a constant potential of platinum gauze (+2 V vs SCE), the recorded spectrum exhibited a significant drop in the EPR signal intensity (Figure S8). Higher values of *E*^oF^ (V^V^/V^IV^), observed for **3**-Bmim and **4**-Bmim, are ascribed to the + *I* effect of the alkyl tails of oedta and heedta, respectively. Another oxidation process was observed in the case of the ionic liquids **1**-Bmim and **2**-Bmim. It represents a one-electron oxidation, probably located at the periphery of the edta and dcta ligands. Moreover, in the case of **2**-Bmim, this process is electrochemically irreversible.

### Catalytic Activity of Ionic Liquids

**Scheme 5:**
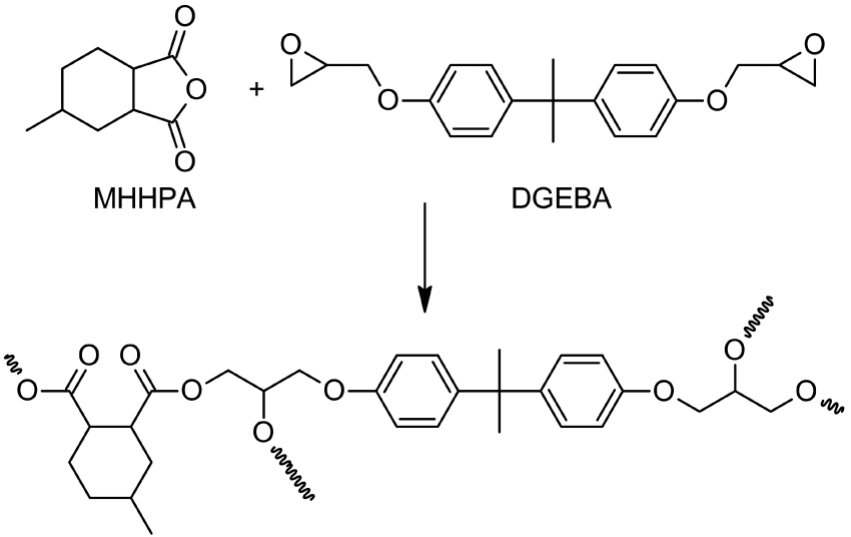
Ring-Opening Copolymerization of Epoxy Resin (DGEBA) with Cyclic Anhydride (MHHPA)

The ability of vanadium-containing ILs to serve as catalysts for epoxy/anhydride copolymerization was investigated on formulations of bisphenol A diglycidyl ether (DGEBA) and hexahydro-4-methylphthalic anhydride (MHHPA) (Scheme 5). For this purpose, a series of ILs containing the complexes with edta and oedta ligands were used to examine the effect of mono/dianionic vanadium species on the catalytic activity. To cover the effect of ligand periphery imidazolium ILs **2**-Bmim and **4**-Bmim were chosen as the representatives of dcta and heedta complexes.

First, the uncatalyzed DGEBA/MHHPA system was tested showing a low reaction enthalpy (Δ*H*_R_ of 192 J/g, Table 3) and a high *T*_onset_ value (192 °C, Table 3), which indicates an incomplete/partial curing reaction (see also the DSC record in Figure S9). In contrast, all tested DGEBA/MHHPA systems containing 2.7 mol % of vanadium-based ILs showed a well-defined reaction exotherm (Figure S9) with the corresponding Δ*H*_R_ values in the range of 295–319 J/g (Table 3), proving the progress of copolymerization reaction between DGEBA and MHHPA. The Δ*H*_R_ values are similar to those of the reference system using a conventional 1-methylmidazole catalyst (Δ*H*_R_ = 297 J/g, Table 3) and to those previously reported for different catalysts (e.g., *N,N*-dimethylbenzylamine, Δ*H*_R_ = 289 J/g),^45^ ILs (e.g., 1-butyl-3-methylimidazolium chloride, Δ*H*_R_ = 334 J/g)^33^ and TM-ILs (e.g., [Bmim][FeCl_4_], Δ*H*_R_ = 343 J/g; [Bmim]_2_[CoCl_4_], Δ*H*_R_ = 327 J/g; [Bmim]_2_[ZnCl_4_], Δ*H*_R_ = 331 J/g).^33^ This fact together with the low *T*_onset_ values (Table 3) and the absence of any exotherms connected to a residual heat during the second DSC heating run (Figure S10) proves an efficient catalysis by vanadium-containing ILs and complete cross-linking. The type of cation (ammonium, imidazolium, or phosphonium) and anion (mono or dianionic vanadium complex) exerted a minor effect on the onset temperature (*T*_onset_) since the values were in the narrow range of 100–115 °C (Table 3). Nevertheless, the DGEBA/MHHPA system containing **1**-Bmim exhibited the highest glass transition temperature (*T*_g_) after curing (130 °C, Table 3) comparable to that for the reference system (137 °C, Table 3), whereas using **1**-Bu_4_P resulted in the lowest *T*_g_ (112 °C, Table 3). Since both **1**-Bmim and **1**-Bu_4_P contain the same anion, it is suggested that the type of IL-anion affects the structure and cross-link density of the produced epoxy networks. These results are in good accordance with previous findings demonstrating the crucial role of anions of the imidazolium-based ILs on the curing epoxy reaction and the resulting network structure.^27,33^

**Table 3:** Dynamic DSC Results of Reactive Mixtures DGEBA/MHHPA = 1/1 and 2.7 mol % of IL at a Heating Rate 5 °C/min^a^

system	*T*_onset_ (°C)^b^	*T*_max_ (°C)^c^	Δ*H*(J/g)^d^	*T*_g_ (°C)^e^
no catalyst	192	237	192	110
reference	109	131	297	137
1-Bu_4_N	106	128	316	125
1-Bmim	104	130	319	130
1-Bu_4_P	103	122	297	112
2-Bmim	100	125	298	120
3-Bu_4_N	115	139	311	120
3-Bmim	114	141	297	125
3-Bu_4_P	108	135	295	123
4-Bmim	105	135	316	122

a The reference system contained 2.7 mol % of 1-methylimidazole.

b Onset temperature.

c Maximal peak temperature.

d Total reaction heat.

e Midpoint determined from the second heating DSC run after curing.

In summary, the newly synthesized vanadium-based ILs showed a catalytic effect on the epoxy/anhydride reaction leading to the production of high *T*_g_ epoxy networks. Thus, these novel ILs can be considered as promising components in epoxy-anhydride formulations.

### Kinetics of Isothermal Cross-Linking of Epoxy-Anhydride with Ionic Liquids

**Figure 9:**
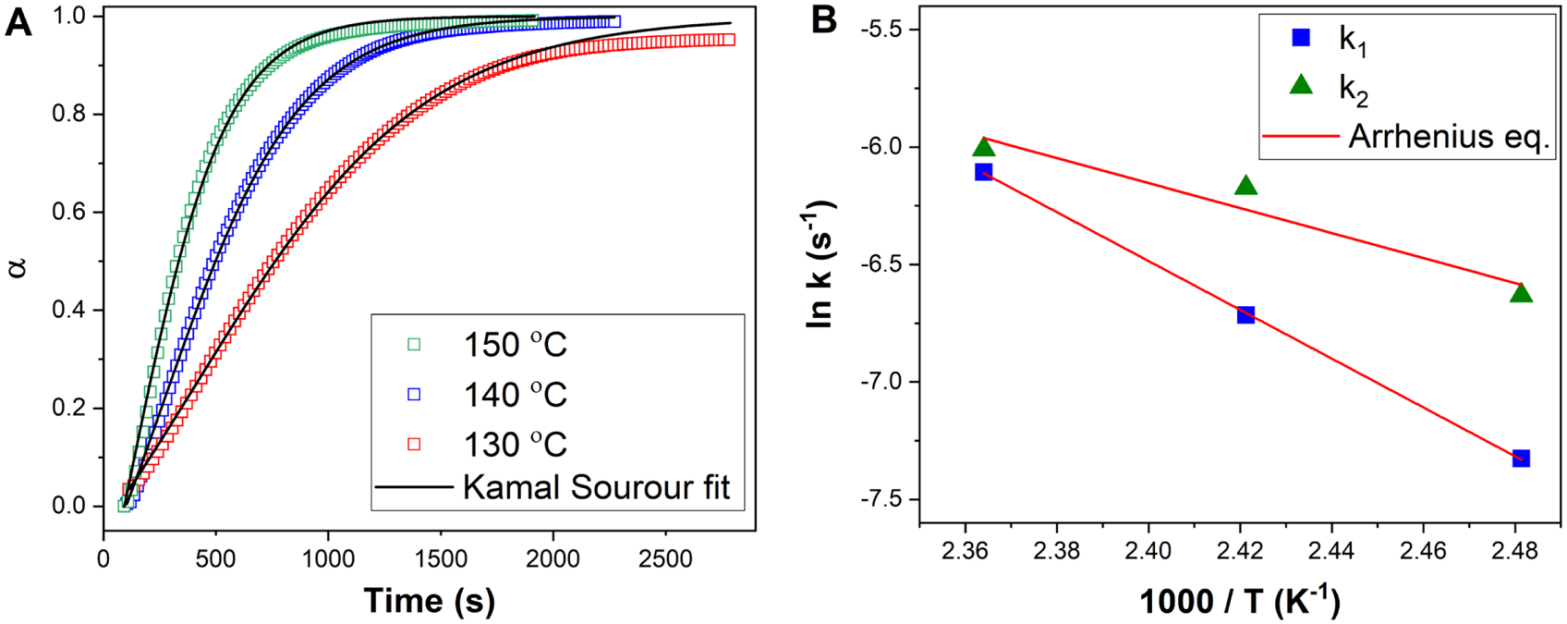
A) Comparison of experimental fitted values of DSC conversion (α) during isothermal cross-linking of DGEBA/MHHPA (1:1) treated with **2**-Bmim (1 wt %). Point: experimental values; Black solid lines: fit by Kamal-Sourour model (*m* = 1, *n* = 1). B) Temperature dependence of reaction rate constants *k*_1_ and *k*_2_ determined from the isothermal polymerization of DGEBA/MHHPA/**2**-Bmim. Red solid lines present Arrhenius plots.

Dynamic DSC measurements showed a high catalytic efficiency of **2**-Bmim for DGEBA-MHHPA polymerization. Therefore, this IL was selected for the study of kinetics, which will allow us to determine the kinetic parameters of epoxy-anhydride cross-linking and to better understand the mechanism of polymerization. For a better comparison with conventional catalytic systems, the content of **2**-Bmim was reduced to 1% wt. Isothermal DSC runs were performed at the temperature range of 120–140 °C, and the conversion values (α) determined from eq S1 are present in Figure 9A.

It is evident from the shape of the conversion curves that there is a significant reduction in the induction curing period (the traditional sigmoidal conversion curve does not appear). Then, the experimentally obtained data were fitted to the Kamal-Sourour model (eqs S2 and S3), traditionally used for description of epoxy kinetics (see Supporting Information for details). Based on the literature^46^ and our previous experiments,^33^ the overall reaction order was initially fixed (*m* + *n* = 2), while the fitting adjustment of the partial reaction orders *m* and *n* was found to be 1 for both (*m* = *n* = 1). The same result of the fitting was previously reported for DGEBA-MHHPA reaction catalyzed by the conventional imidazole catalyst.^33^ For all isothermal conditions, the model simulations correlated well with the experimental data up to a conversion of around 0.95. The system subsequently undergoes vitrification, while the further course of the reaction is controlled by diffusion.^47^ It can therefore be stated that the selected model can be successfully applied to describe the course of the chemically controlled reaction (up to the point of vitrification) of the DGEBA-MHHPA/**2**-Bmim system.

Generally, it is known that due to the low reactivity of epoxy groups toward anhydride, catalysts play a crucial role in epoxy/anhydride cross-linking, as they not only accelerate curing but also significantly change its mechanism.^48^ The main reaction pathway of epoxide/anhydride cross-linking catalyzed by common catalysts (typically imidazoles or tertiary amines) proceeds as an anionic alternating copolymerization, initiated by alkoxide anions that further react with anhydride giving a carboxylate anion. To a minor extent, an uncatalyzed epoxy-anhydride reaction also takes place, namely by a different step-growth (polyaddition) mechanism, usually initiated by OH-containing impurities or traces of water, which react with the anhydride to form a carboxylic acid.^49^ The Kamal-Sourour model is highly suitable to describe the overall epoxy-anhydride cross-link process, because it uses two rate constants, *k*_1_ and *k*_2_, corresponded to the uncatalyzed and catalyzed reaction, respectively. The calculated rate constants describing the course of cross-linking under **2**-Bmim catalysis are shown in Figure 9B. The temperature dependence of both *k*_1_ and *k*_2_ constants can be well fitted by the Arrhenius equation (eq S4), giving values of the activation energy of the uncatalyzed (*E*_a1_) and catalyzed (*E*_a2_) reactions of 86 and 44 kJ/mol, respectively. These results are consistent with literature data showing a higher activation barrier for the initial uncatalyzed reaction compared to the catalyzed one.^50^ Moreover, the value of the activation energy of the uncatalyzed reaction (*E*_a1_) lies in the range of values typical for anhydride-cured epoxies (60–90 kJ/mol).^49,51,52^ In contrast, the *E*_a2_ value was found to be significantly lowered, which indicates the effective catalysis of **2**-Bmim during the propagation step of the anionic chain-growth copolymerization.^52^

Aiming to better clarify the polymerization mechanism of epoxy-anhydride in the presence of **2**-Bmim, the reaction at elevated temperature was monitored by the *in situ* EPR spectroscopy and near-infrared spectroscopy. These methods allowed to follow paramagnetic vanadium(IV) species and the evolution of individual functional groups (epoxide, anhydride, ester, hydroxy and moisture) in the formulation.

**Figure 10:**
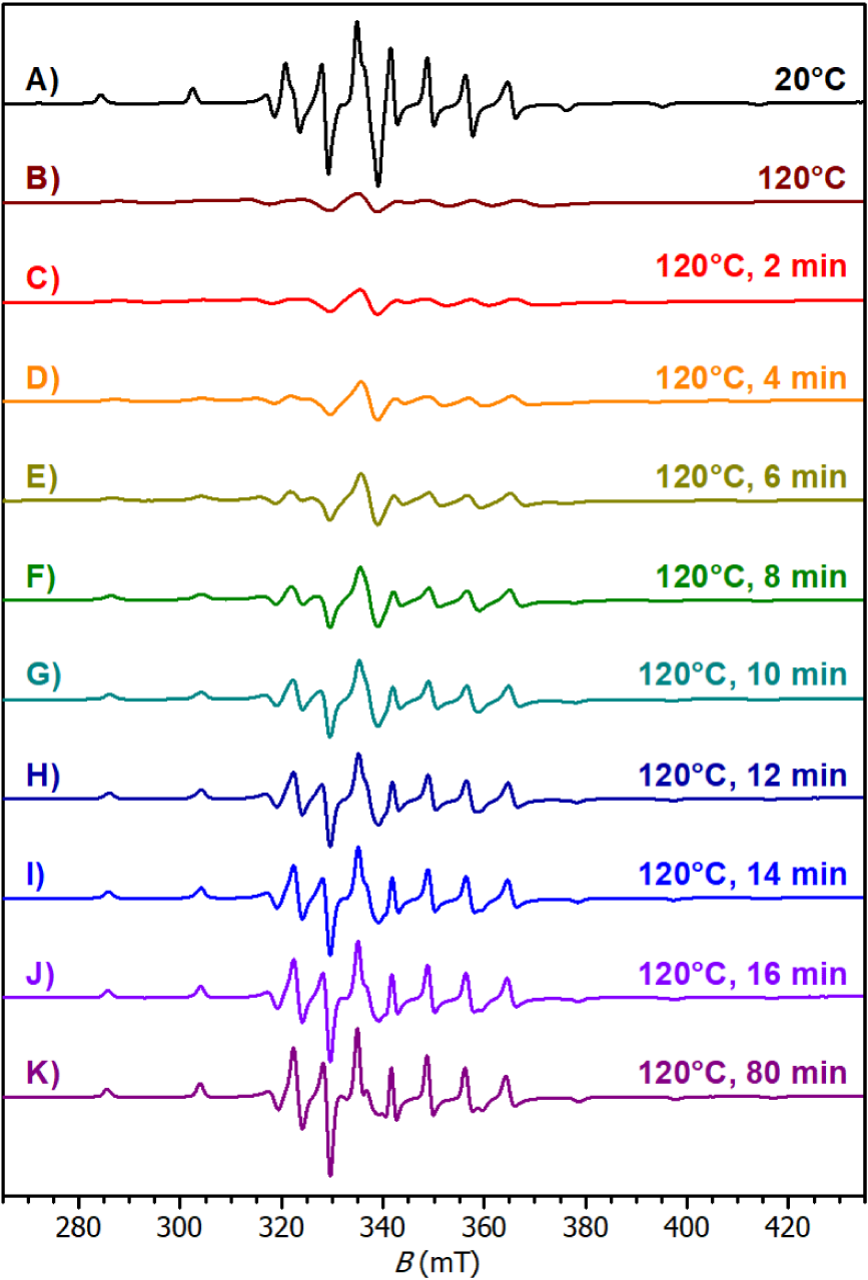
Curing process followed by *in situ* EPR spectroscopy at isothermal conditions (120 °C). Formulation: DGEBA/MHHPA (1:1) treated with **2**-Bmim (1 wt %). A) Reference spectrum of the fresh formulation at 20 °C. B) Fresh formulation at 120 °C. C) Sample cured for 2 min. D) Sample cured for 4 min. E) Sample cured for 6 min. F) Sample cured for 8 min. G) Sample cured for 10 min. H) Sample cured for 12 min. I) Sample cured for 14 min. J) Sample cured for 16 min. K) Sample cured for 80 min.

At room temperature, fresh formulation of **2**-Bmim in DGEBA-MHHPA (1 wt %) gives an anisotropic spectrum with a well-resolved hyperfine structure; *A*_∥_ = 18.70 mT, *A*_⊥_ = 6.50 mT, *g*_∥_ = 1.943, *g*_⊥_ = 1.977 (Figure 10, Spectrum A). The spectrum pattern nears the frozen solution mentioned earlier due to high viscosity of the formulation. It proves full dissolution of the complex vanadium(IV) species without appearance of colloid species. Heating of the formulation at 120 °C results in broadening of line widths and loss of the anisotropic nature due to considerable viscosity decrease (Figure 10, Spectrum B). Spectra C–K in the Figure 10 document the isothermal curing of the epoxy-anhydride formulation at 120 °C. During the first ∼14 min, slow sharpening of line widths is observed, which reflects deceleration of the molecular motion due to increasing viscosity of the formulation and sol–gel transformation. After that, clear anisotropic spectra (*A*_∥_ = 18.73 mT, *A*_⊥_ = 6.50 mT, *g*_∥_ = 1.930, *g*_⊥_ = 1.974) were recorded with minor changes in the pattern and intensity, proving high stability of the complex species under the harsh conditions of the curing process without changes in the coordination sphere of vanadium(IV).

**Figure 11:**
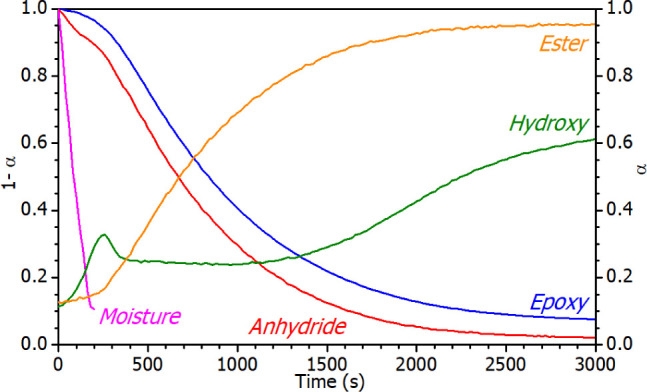
Conversions of function groups according to time-resolved NIR spectroscopy at isothermal conditions (140 °C). Formulation: DGEBA/MHHPA (1:1) treated with **2**-Bmim (1 wt %). Left *y*-axis (1-α) for epoxy, anhydride and moisture; Right *y*-axis (α) for ester and hydroxyl.

As documented in Figure 11, in the initial phase of polymerization, the anhydride groups decreased slightly faster than the epoxy rings, and at the same time, there is a sharp decrease in the water content present in the system due to the hygroscopicity of **2**-Bmim. A similar phenomenon was observed during epoxy-anhydride cross-linking in the presence of TM-ILs bearing MCl_4_ anion where the formed anhydride-MCl_4_-anion complex accelerated the carboxylic acid-epoxy reaction producing a polyester chain.^33^ Herein, the attack of water molecules leading to hydrolysis of the cyclic anhydride is also accelerated by **2**-Bmim, which resulted in the formation of hydroxyesters (the initial hydroxy group increase is visible by NIR, Figure 11). The subsequent propagation step comprised further hydroxyester-anhydride reaction yielding the alternating epoxy-anhydride copolymer. As observed before, this polyester route mainly affects the *k*_1_ rate constant.^33^ However, herein *k*_2_ > *k*_1_ for all tested temperatures, which means that the catalyzed pathway (*k*_2_) is dominant and the overall cross-linking is mainly driven by a catalytic mechanism. This mechanism is probably initiated by the imidazolium cation of **2**-Bmim, similar to other imidazolium ILs.^27,33,53^ It is known that imidazolium ILs initiate the epoxy ring opening via three main routes: carbene formation, imidazolium decomposition (“imidazole” route) and counterion route (anion nucleophilic attack).^53,54^ Herein, the counteranion pathway is less probable due to the steric effects of the **2**-Bmim anion. Therefore, we assume the initiation via either the “carbene route” comprising deprotonation of the imidazolium ring, or the “imidazole route” consisting of dealkylation of the imidazolium ring and subsequent attack of this species on the epoxy carbon, or a combination of both mechanisms. However, the precise determination of the mechanism requires additional experiments, and therefore a more detailed study devoted to the mechanism of epoxy-anhydride copolymerization in the presence of vanadium-containing ILs will be the subject of a separate contribution.

## Conclusions

This study described a new type of vanadium-containing ILs consisting of organic cation and anionic oxidovanadium(IV) complexes stabilized by edta and its congeners. They are accessible from appropriate barium salts by cation exchange. The barium precursors (**1**-Ba, **2**-Ba, **3**-Ba, and **4**-Ba) were characterized by analytical methods including single-crystal XRD analysis. The process of cation exchange was initially examined on inorganic salts and then used for the introduction of organic cations. Our detailed investigation of the vanadium-containing ILs has shown that modification of the edta ligand has only a minor effect on the coordination sphere of vanadium, as documented by the EPR spectroscopy, but the differences in their redox properties are significant. Monoanionic complexes bearing oedta and heedta show only expected one-electron oxidation of vanadium(IV), but the dianionic species bearing edta and dcta further show an oxidation on the ligand periphery, stabilized by an uncoordinated carboxylate function. The catalytic properties of vanadium-containing ILs were exemplified in the ring-opening copolymerization of epoxy resin with cyclic anhydride. The dynamic DSC runs have shown that the ILs described here serve as effective catalysts of epoxy-anhydride copolymerization enabling fast and complete curing. A detailed isothermal kinetic study was performed using isothermal DSC measurements, and the Kamal-Sourour model was adopted. The model fitting enabled the calculation of rate constants and respective activation energies and proved a dominant catalyzed pathway. The isothermal DSC and NIR measurements showed the accelerating effect of vanadium-containing IL on the hydrolysis of cyclic anhydride and the subsequent reaction of the carboxyl groups leading to the formation of a polyester chain. In parallel, the involvement of cationic (imidazolium) part of the vanadium-containing IL in the initiation mechanism of epoxy-anhydride anionic chain-growth copolymerization is assumed, via either the “carbene route” or the “imidazole route”. Nevertheless, detailed investigations of the mechanism using vanadium-containing ILs and the curing process will be the subject of another study. EPR measurements have proved high stability of selected vanadium(IV) complex during the curing process.

## Experimental Section

### Materials

1-Bromooctane ethylenediamine, chloroacetic acid, H_4_edta, H_4_dcta, H_3_heedta, VOSO_4_·3H_2_O, Ag_2_SO_4_, BaCO_3_, Cs_2_SO_4_, MgSO_4_·7H_2_O, (Bu_4_N)Cl and (Bu_4_P)Br were supplied from Acros Organics. Bisphenol A diglycidyl ether (DGEBA, EEW = 179 g/mol) was kindly provided from DOW chemicals. Hexahydro-4-methylphthalic anhydride (MHHPA, 96%) and 1-methylimidazole were purchased by Sigma-Aldrich. A literature procedure was used for the preparation of (Bmim)Cl.^55^

Syntheses of compounds **1**-Ba, **1**-Bu_4_N, **1**-Bmim, **1**-Bu_4_P, **2**-Ba, **2**-Bmim, **3**-Ba, **3**-Bmim, **4**-Ba and **4**-Bmim are outlined in detail as an example of the general methodology for the synthesis of the compounds reported here. Synthetic details of all other new compounds and spectroscopic and analytic data are available in the Supporting Information.

### Methods

^1^H NMR spectra were measured on the Bruker Avance 500 MHz spectrometer. The spectra were calibrated to the residual signal of the solvent relative to Me_4_Si. EPR spectra were measured on a Miniscope MS 3000 spectrometer in the microwave X-band (∼9.5 GHz). The fluid and frozen solution spectra were measured in glass capillaries (*ID* = 0.5 mm) at room temperature (293 K) and at 123 K, respectively. The obtained spectra were computer-simulated using the EPR simulation software SimFonia version 1.2 (Bruker). Second-order perturbation theory was used for a description of the interaction between electronic spin and nuclear spin of vanadium. Anisotropic line widths and mixed Lorentzian/Gaussian line shapes were used for the simulations. Curing process was followed in the glass capillaries (*ID* = 0.5 mm) at 393 K.

Electrochemical measurements were carried out in MeCN containing 0.1 M Bu_4_NPF_6_ in a three-electrode cell by cyclic voltammetry (CV). The working electrode was platinum or glassy carbon disk (*d* = 2 mm) for CV experiments. Saturated calomel electrode (SCE), separated by a bridge filled with a supporting electrolyte, and Pt wire were used as the reference and auxiliary electrodes, respectively. All potentials are given vs SCE. Voltammetric measurements were made using a potentiostat PGSTAT 128N (AUTOLAB, Metrohm Autolab B.V., Utrecht, The Netherlands) operated using NOVA 1.11 software.

Mass spectra were collected on a quadruple mass spectrometer LCMS 2010 (Shimadzu, Japan). The samples were dissolved in water and injected into the mass spectrometer with an infusion mode at a constant flow rate of 10 μL min^–1^. Electrospray ionization mass spectrometry (ESI-MS) was used for the identification of the analyzed samples. The *M* symbol denotes anionic vanadium complexes as defined in Schemes 2–4.

The vanadium, barium, phosphorus, and sulfur contents were determined by inductively coupled plasma-optical emission spectroscopy (ICP). Samples were precisely weighed (∼0.05 g), treated with nitric acid (7 mL) and allowed to react for 20 min in an open vessel before being decomposed in a Speedwave Xpert microwave mineralizer (Berghof, Tübingen, Germany) at 175 °C for 15 min and 220 °C for 25 min. The mineralized sample was then diluted and analyzed on a ICP OES spectrometer INTEGRA 6000 (GBC, Dandenong, Australia), equipped with the concentric nebulizer and the glass cyclonic spray chamber (both Glass Expansion, Australia).

Thermogravimetric analysis (TGA) was performed to quantify a water content and to determine thermal stability of the prepared vanadium-based ILs. TGA measurements were carried out using a thermogravimetric analyzer Pyris 1 TGA (PerkinElmer, USA) under nitrogen flow of 25 cm^3^ min^–1^. A sample of ca. 15 mg was heated from 30 to 600 °C at a heating rate of 10 °C min^–1^.

Differential scanning calorimetry (DSC) analyzes of prepared vanadium-based ILs were carried out on a DSC Q2000 (TA Instruments, USA) with nitrogen purge gas (50 cm^3^ min^–1^). The instrument was calibrated for temperature and heat flow using indium as a standard. Samples of about 5–10 mg were encapsulated into hermetically sealed Tzero aluminum pans with a pinhole. DSC runs were performed with a ramp rate of 10 °C min^–1^ using a heating–cooling–heating cycle from −60 to 200 °C. Fifteen-minute isothermal plateau at 200 °C was inserted after the first heating run to remove moisture from the samples. Glass transition temperature (*T*_g_) was determined as a step change midpoint (half-height) on the second heating DSC curves.

### Tests of Catalytic Activity for Epoxy-Anhydride Copolymerization

Dynamic DSC measurements were performed to determine the catalytic activities of the prepared vanadium-based ILs in the reactive mixture of epoxy monomers using a heat flux DSC calorimeter Q2000 (TA Instruments, USA) calibrated for indium. The stoichiometric amount of epoxy resin (DGEBA) and anhydride (MHHPA) were mixed with 2.70 mol % of vanadium-based ILs (various cations and anions) and homogenized using a magnetic stirrer. Then, the reactive mixture (ca. 10 mg) was immediately introduced into a hermetically sealed Tzero aluminum pan with a pinhole and measured under a nitrogen purge of 50 mL/min from 20 to 300 °C at a heating rate of 5 °C min^–1^. Subsequently, an additional ramp DSC run at 10 °C min^–1^ was performed to determine the glass transition temperature (*T*_g_) of the cured epoxy networks.

Based on the nonisothermal DSC runs, the most promising vanadium-based IL was selected and used for kinetic study of epoxy-anhydride copolymerization using isothermal DSC measurements. Experimental details are given in Supporting Information.

### Crystallography

Data for **1**-Ba·6H_2_O, **1**-Mg·9H_2_O·0.5(dioxane), **1**-Cs·2H_2_O, **2**-Ba·6H_2_O, **3**-Ba·7H_2_O·2^i^PrOH, **4**-Ba·3H_2_O, **4**-Mg·8H_2_O, and **4**-Cs·H_2_O were collected on the Rigaku XtaLAB Synergy S diffractometer equipped with microfocus CuKα/MoKα radiation and a Hybrid Pixel Array Detector (HyPix-6000HE). An Oxford Cryosystems (Cryostream 800) cooling device was used for data collection and crystals were kept at 100 K during data collection. CrysAlisPro software^56^ was used for data collection, cell refinement and data reduction. Data were corrected for absorption effects using empirical absorption correction (spherical harmonics), implemented in SCALE3 ABSPACK scaling algorithm and numerical absorption correction based on Gaussian integration over a multifaceted crystal model. Using Olex2,^57^ the structures were solved with the SHELXT^58^ structure solution program and refined with the SHELXL^59^ refinement package using Least Squares minimization. Most hydrogen atom positions were calculated geometrically and refined using the riding model, but some hydrogen atoms were refined freely.

### Synthesis of (Bu_4_N)_2_SO_4_

A suspension of Ag_2_SO_4_ (1.06 g, 3.40 mmol) in hot distilled water (100 mL; 80 °C) was treated with a solution of (Bu_4_N)Cl (2.20 g, 6.82 mmol) in distilled water (10 mL), stirred for 30 min and filtered. The filtrate was dried in an oven at 50 °C and then vacuum evaporated at 100 °C. The product was stored under an inert atmosphere of argon. Yield: 1.50 g (1.24 mmol, 36.5%). Colorless viscous liquid. Anal. Calc. for (Bu_4_N)_2_SO_4_·35H_2_O (C_32_H_142_N_2_O_39_S): C, 31.72; H, 11.81; N, 2.31; S, 2.65. Found: C, 31.60; H, 11.38; N, 2.53; S, 2.72. ICP Calc.: S, 2.65. Found: S, 2.65.

### Synthesis of *N*-Octylethylenediamine

A solution of freshly distilled ethylenediamine (40 mL, 36 g, 599 mmol) in absolute ethanol (100 mL) was treated with 1-bromooctane (35 mL, 39 g, 202 mmol) and heated under reflux for 17 h. Ethanol was vacuum evaporated and the remaining mixture formed two layers. The top layer was separated and purified by vacuum distillation. Yield: 19.9 g (0.116 mmol; 57.3%). Colorless liquid. Bp: 120 °C (170 Pa). The analytical data were in line with those published elsewhere.^60^

### Synthesis of *N*-Octylethylenediaminetriacetic Acid (H_3_oedta)

A chloroacetic acid solution (9.90 g, 105 mmol) in distilled water (12 mL) was cooled to 0 °C and treated dropwise with a precooled potassium hydroxide solution (11.7 g, 209 mmol) in distilled water (15 mL) to ensure that the temperature of the solution does not exceed 20 °C. The reaction mixture was treated with *N*-octylethylenediamine (2 g, 11.6 mmol) and stirred in closed vessels at room temperature for 7 d. The solution was cooled to 0 °C and slowly acidified with hydrochloric acid (35 wt %). At pH = 2.12 (9 mL of HCl was added), fine power starts to precipitate. The suspension was stored at 10 °C for 15 h. The product was filtered and washed with water and diethyl ether. Finally, it was dried in an oven at 50 °C until a constant mass. Yield: 1.76 g (5.08 mmol, 43.8%). The analytical and spectroscopic data were in line with those published elsewhere.^61^

### Synthesis of Ba[VO(edta)] (1-Ba)

A solution of VOSO_4_·3H_2_O (5.43 g, 25.0 mmol) in distilled water (100 mL) was treated with H_4_edta (7.31 g, 25.0 mmol) and stirred at 80 °C for 30 min. The reaction mixture was treated with BaCO_3_ (12.3 g, 62.5 mmol) in several portions and stirred vigorously. When the evolution of carbon dioxide stopped, the reaction mixture was filtered and the volume of the filtrate was reduced to 50 mL by vacuum evaporation on rotavapor. After storage at 10 °C for 16 h, blue crystals of the product were collected by filtration and dried in an oven at 50 °C. Yield: 11.4 g (19.0 mmol; 76.1%). Blue crystals. Anal. Calc. for **1**-Ba·6H_2_O (C_10_H_24_BaN_2_O_15_V): C, 20.00; H, 4.03; N, 4.66. Found: C, 20.20; H, 3.85; N, 4.48. ICP Calc.: V, 8.48; Ba, 22.87. Found: V, 8.64; Ba, 21.19. ESI-MS (H_2_O), *m*/*z*, negative-ion: 177.5 (100%) [M]^2–^, 186.5 [M + H_2_O]^2–^, 356 [M + H]^−^. EPR (water): *g*_iso_ = 1.970, |*A*_iso_| = 10.41 mT. Single crystals of **1**-Ba·6H_2_O suitable for XRD analysis were prepared by slow evaporation of the aqueous solution of **1**-Ba.

### Synthesis of (Bu_4_N)_2_[VO(edta)] (1-Bu_4_N)

Compound **1**-Ba·6H_2_O (1.20 g, 2.0 mmol) was dissolved in hot distilled water (10 mL; 80 °C), treated with a solution of (Bu_4_N)_2_SO_4_·35H_2_O (2.47 g, 2.04 mmol) in distilled water (5 mL), stirred for 30 min, filtered and the filtrate was vacuum-dried at 100 °C to a constant mass. The product was stored under an inert atmosphere of argon. Upon several days, the blue oily product crystallized. Yield: 1.06 g (1.19 mmol; 59.5%). Blue solid. Anal. Calc. for **1**-Bu_4_N·3H_2_O (C_42_H_90_N_4_O_12_V): C, 56.42; H, 10.15; N, 6.27. Found: C, 56.65; H, 10.30; N, 6.44. ICP Calc.: V, 5.70. Found: V, 5.64. ESI-MS (H_2_O), *m*/*z*, positive-ion: 242 (100%) [Bu_4_N]^+^; negative-ion: 177.5 (100%) [M]^2–^, 186.5 [M + H_2_O]^2–^, 356 [M + H]^−^, 597 [M + Bu_4_N]^−^. EPR (water): *g*_iso_ = 1.969, |*A*_iso_| = 10.43 mT; EPR (MeCN): *g*_iso_ = 1.967, |*A*_iso_| = 10.48 mT.

### Synthesis of (Bmim)_2_[VO(edta)] (1-Bmim)

Compound **1**-Ba·6H_2_O (2.58 g, 4.31 mmol) was dissolved in hot distilled water (25 mL; 80 °C), treated with a solution of (Bmim)_2_SO_4_·12H_2_O (2.54 g, 4.30 mmol) in distilled water (10 mL), stirred for 30 min, filtered and the filtrate was vacuum-dried at 100 °C to a constant mass. The product was stored under an inert atmosphere of argon. Yield: 1.68 g (2.27 mmol, 52.8%). Blue viscous liquid. Anal. Calc. for **1**-Bmim·6H_2_O (C_26_H_54_N_6_O_15_V): C, 42.10; H, 7.34, N: 11.33. Found: C, 42.32; H, 7.13; N, 11.46. ICP Calc.: V, 6.87. Found: V, 6.81. ESI-MS (H_2_O), *m*/*z* (%), positive-ion: 139 (100) [Bmim]^+^; negative-ion: 356 (100) [M + H]^−^. EPR (water): *g*_iso_ = 1.968, |*A*_iso_| = 10.40 mT; EPR (MeCN): *g*_iso_ = 1.968, |*A*_iso_| = 10.47 mT.

### Synthesis (Bu_4_P)_2_[VO(edta)] (1-Bu_4_P)

Compound **1**-Ba·6H_2_O (1.03 g, 1.72 mmol) was dissolved in hot distilled water (10 mL; 80 °C), treated with a solution of (Bu_4_P)_2_SO_4_·5H_2_O (1.21 g, 1.72 mmol) in distilled water (5 mL), stirred for 30 min, filtered and the filtrate was vacuum-dried at 100 °C to a constant mass. The product was stored under an inert atmosphere of argon. Yield: 1.19 g (1.28 mmol; 74.4%). Blue solid. Anal. Calc. for **1**-Bu_4_P·3H_2_O (C_42_H_90_N_2_O_12_P_2_V): C, 54.36; H, 9.77; N, 3.02. Found: C, 54.65; H, 9.76; N, 2.97. ICP Calc.: V, 5.49; P, 6.67. Found: V, 5.60; P, 6.69. ESI-MS (H_2_O), *m*/*z*, positive-ion: 259 (100%) [Bu_4_P]^+^; negative-ion: 177.5 [M]^2–^, 356 (100%) [M + H]^−^, 614 [Bu_4_P + M]^−^. EPR (water): *g*_iso_ = 1.969, |*A*_iso_| = 10.41 mT; EPR (MeCN): *g*_iso_ = 1.969, |*A*_iso_| = 10.48 mT.

### Synthesis of Ba[VO(dcta)] (2-Ba)

A solution of VOSO_4_·3H_2_O (2.00 g, 9.21 mmol) in distilled water (50 mL) was treated with H_4_dcta (2.95 g, 8.52 mmol) and stirred at 80 °C for 30 min. The reaction mixture was treated with BaCO_3_ (3.36 g, 17.0 mmol) in several portions and vigorously stirred. When the evolution of carbon dioxide stopped, the reaction mixture was filtered. When the solution was cooled to room temperature, a blue-green product precipitated. The mother liquor was decanted and the solvent was evaporated at room temperature in a crystallization dish to reach the second crop of the product. Both crops were dried in an oven at 50 °C. Yield: 4.84 g (7.40 mmol; 86.8%). Blue crystals. Anal. Calc. for **2**-Ba·6H_2_O (C_14_H_30_BaN_2_O_15_V): C, 25.69; H, 4.62; N, 4.28. Found: C, 25.84; H, 4.56; N, 4.18. ICP Calc.: V, 7.78; Ba, 20.98. Found: V, 7.64; Ba, 21.24. ESI-MS (H_2_O), *m*/*z*, negative-ion: 205 [M]^2–^, 214 [M + H_2_O]^2–^, 410 (100%) [M + H]^−^, 478 [2 M + Ba]^2–^. EPR (water): *g*_iso_ = 1.968, |*A*_iso_| = 10.29 mT. Single crystals of **2**-Ba·6H_2_O suitable for XRD analysis were prepared by slow evaporation of the aqueous solution of **2**-Ba.

### Synthesis of (Bmim)_2_[VO(dcta)] (2-Bmim)

Compound **2**-Ba·6H_2_O (664 mg, 1.01 mmol) was dissolved in hot distilled water (20 mL; 80 °C), treated with a solution of (Bmim)_2_SO_4_·12H_2_O (594 mg, 1.01 mmol) in distilled water (5 mL), stirred for 30 min and filtered. The filtrate was dried in a drying over at 60 °C. The solid residue was dissolved in MeCN (20 mL), filtered and the solvent was vacuum evaporated at 100 °C to a constant mass. The product was stored under an inert atmosphere of argon. Yield: 813 mg (0.85 mmol; 84.2%). Blue viscous liquid. Anal. Calc. for **2**-Bmim·15H_2_O (C_30_H_78_N_6_O_24_V): C, 37.62; H, 8.21; N, 8.77. Found: C, 37.47; H, 8.08; N, 8.61. ICP Calc.: V, 5.32. Found: V, 5.27. ESI-MS (H_2_O), *m*/*z*, positive-ion: 139 (100%) [Bmim]^+^; negative-ion: 205 [M]^2–^, 410 (100%) [M + H]^−^. EPR (water): *g*_iso_ = 1.969, |*A*_iso_| = 10.32 mT; EPR (MeCN): *g*_iso_ = 1.969, |*A*_iso_| = 10.36 mT.

### Synthesis of Ba[VO(oedta)]_2_ (3-Ba)

A solution of VOSO_4_·3H_2_O (659 mg, 3.03 mmol) in distilled water (25 mL) was treated with H_3_oedta (1.05 g, 3.03 mmol) and stirred at 80 °C for 30 min. The reaction mixture was treated with BaCO_3_ (1.50 g, 7.60 mmol) in several portions and vigorously stirred. When the evolution of carbon dioxide stopped, the reaction mixture was filtered and the volume of the filtrate was reduced to half by vacuum evaporation on rotavapor. The solution was treated with acetone (10 mL) and stored at 10 °C for 16 h. The blue crystals of the product were collected by filtration and dried in an oven at 50 °C. Yield: 613 mg (0.57 mmol; 38.0%). Blue crystals. Anal. Calc. for **3**-Ba·6H_2_O (C_32_H_66_BaN_4_O_20_V_2_): C, 36.05; H, 6.24; N, 5.26. Found: C, 35.77; H, 6.44; N, 5.43. ICP Calc.: V, 9.56; Ba, 12.88. Found: V, 9.47; Ba, 13.07. ESI-MS (H_2_O), *m*/*z*, negative-ion: 410 (100%) [M]^−^. EPR (water): *g*_iso_ = 1.970, |*A*_iso_| = 10.34 mT. Crystals of **3**-Ba·7H_2_O·2^i^PrOH suitable for XRD analysis were prepared by slow diffusion of isopropanol vapors into an aqueous solution of **3**-Ba.

### Synthesis of (Bmim)[VO(oedta)] (3-Bmim)

Compound **3**-Ba·6H_2_O (906 mg, 0.85 mmol) was dissolved in hot distilled water (10 mL; 80 °C) and treated with a solution of (Bmim)_2_SO_4_·12H_2_O (502.0 mg, 0.85 mmol) in distilled water (6 mL), stirred for 30 min and filtered. The filtrate was vacuum-dried at 100 °C to a constant mass. The product was stored under an inert atmosphere of argon. Yield: 1.01 g (1.46 mmol; 86.9%). Blue viscous liquid. Anal. Calc. for **3**-Bmim·8H_2_O (C_24_H_58_N_4_O_15_V): C, 41.56; H, 8.43, N: 8.08. Found: C, 41.80; H, 8.18; N, 8.31. ICP Calc.: V, 7.34. Found: V, 7.13. ESI-MS (H_2_O), *m*/*z*, positive-ion: 139 (100%) [Bmim]^+^, 688 [2 Bmim + M]^+^; negative-ion: 410 (100%) [M]^−^. EPR (water): *g*_iso_ = 1.969, |*A*_iso_| = 10.34 mT; EPR (MeCN): *g*_iso_ = 1.968, |*A*_iso_| = 10.53 mT.

### Synthesis of Ba[VO(heedta)]_2_ (4-Ba)

A solution of VOSO_4_·3H_2_O (4.00 g, 18.4 mmol) in distilled water (25 mL) was treated with H_3_heedta (4.35 g, 15.6 mmol) and stirred at 80 °C for 30 min. The reaction mixture was treated with BaCO_3_ (7.27 g, 36.8 mmol) in several portions and stirred vigorously. When the evolution of carbon dioxide stopped, the reaction mixture was filtered. To the filtrate was added 25 mL of BuOH and to the upper layer was added 100 mL of acetone. After several days blue crystals formed from the blue lower layer. The blue crystals were separated from the mother liquor and dried in a drying oven at 50 °C. Yield: 6.81 g (7.78 mmol, 49.8%). Blue crystals. Anal. Calc. for **4**-Ba·3H_2_O (C_20_H_36_BaN_4_O_19_V_2_): C, 27.43; H, 4.14; N, 6.40. Found: C, 27.22; H, 4.26; N, 6.66. ICP Calc.: V, 11.63; Ba, 15.68. Found: V, 11.49; Ba, 15.47. ESI-MS (H_2_O), *m*/*z*, positive-ion: 480 (100%) [M + Ba]^+^; negative-ion: 342 (100%) [M]^−^. EPR (water): *g*_iso_ = 1.969, |*A*_iso_| = 10.41 mT. Large crystals of **4**-Ba·3H_2_O suitable for XRD analysis were prepared by slow diffusion of acetone vapors into an aqueous solution of **4**-Ba overlayered by BuOH.

### Synthesis of (Bmim)[VO(heedta)] (4-Bmim)

Compound **4**-Ba·3H_2_O (779 mg, 0.89 mmol) was dissolved in hot distilled water (20 mL; 80 °C), treated with a solution of (Bmim)_2_SO_4_·12H_2_O (519 mg, 0.88 mmol) in distilled water (5 mL), stirred for 30 min and filtered. The filtrate was dried in an over at 60 °C. The solid residue was dissolved in MeCN (20 mL), filtered and the solvent was vacuum evaporated at 100 °C. The product was stored under an inert atmosphere of argon. Yield: 954 mg (1.53 mmol; 86.0%). Blue viscous liquid. Anal. Calc. for **4**-Bmim·8H_2_O (C_18_H_46_N_4_O_16_V): C, 34.56; H, 7.41; N, 8.96. Found: C, 34.27; H, 7.27; N, 8.74. ICP Calc.: V, 8.14. Found: V, 8.05. ESI-MS (H_2_O), *m*/*z*, positive-ion: 139 (100%) [Bmim]^+^; negative-ion: 342 (100%) [M]^−^. EPR (water): *g*_iso_ = 1.969, |*A*_iso_| = 10.41 mT; EPR (MeCN): *g*_iso_ = 1.968, |*A*_iso_| = 10.51 mT.

## Supporting Information Available

Syntheses, EPR data, XRD data, CV curves, TGA curves, DSC curves, and ESI-MS data (PDF)
