# Supplementary material for: Vanadium-Containing Ionic Liquids Derived from Complexes of Modified Edta as Catalysts of Epoxy-Anhydride Ring-Opening Copolymerization
Source: Inorg Chem. 2024 Aug 29;63(36):16631–44. doi: 10.1021/acs.inorgchem.4c01663 (PMC11388465; doi:10.1021/acs.inorgchem.4c01663)
Supplement: Supplementary file 1 — ic4c01663_si_001.pdf [file ic4c01663_si_001.pdf]

Supporting Information for:

Vanadium-containing ionic liquids derived from complexes of modified edta as catalysts of epoxy-anhydride ring-opening copolymerization

Lukáš Hanzl,[a] Jaromír Vinklárek,[a] Miroslava Litecká,[b] Marwa Rebei,[c] Hynek Beneš,[c] Aleš Eisner,[d] Tomáš Mikysek,[d] Anna Krejčová,[e] and Jan Honzíček *[f]

[a] Department of General and Inorganic Chemistry, Faculty of Chemical Technology, University of Pardubice, Studentská 573, 532 10 Pardubice, Czech Republic.

[b] Department of Materials Chemistry, Institute of Inorganic Chemistry of the CAS, Husinec-Řež 1001, 25068 Řež, Czech Republic.

[c] Institute of Macromolecular Chemistry, Czech Academy of Sciences, Heyrovského nám. 2, Prague 6, 162 00, Czech Republic.

[d] Department of Analytical Chemistry, Faculty of Chemical Technology, University of Pardubice, Studentská 573, 532 10 Pardubice, Czech Republic.

[e] Institute of Environmental and Chemical Engineering, Faculty of Chemical Technology, University of Pardubice, Studentská 573, 532 10 Pardubice, Czech Republic.

[f] Institute of Chemistry and Technology of Macromolecular Materials, Faculty of Chemical Technology, University of Pardubice, Studentská 573, 532 10 Pardubice, Czech Republic.

*Corresponding author: jan.honzicek@upce.cz

Synthesis of $(Bmim)_2SO_4$. A suspension of Ag_2SO_4 (3.62 g, 11.6 mmol) in hot distilled water (200 mL; 80 °C) was treated with a solution of (Bmim)Cl (4.05 g, 23.2 mmol) in distilled water (10 mL), stirred for 30 min and filtered. The filtrate was dried in an oven at 50 °C and residual water was vacuum evaporated at 100 °C. The product was stored under an inert atmosphere of argon. Yield: 4.08 g (6.91 mmol, 59.6%). Colorless solid. Anal. Calc. for $(Bmim)_2SO_4{\cdot}12H_2O$. ($C_{16}H_{54}N_4O_{16}S$): C, 32.53; H, 9.21; N, 9.48; S, 5.43. Found: C, 32.78; H, 8.98; N, 9.61; S, 5.65. ICP Calc.: S, 5.43. Found: S, 5.53.

Synthesis of $(Bu_4P)_2SO_4$. A suspension of Ag_2SO_4 (3.99 g, 12.8 mmol) in hot distilled water (200 mL; 80 °C) was treated with a solution of $(Bu_4P)Br$ (8.65 g, 25.5 mmol) in distilled water (10 mL), stirred for 30 min and filtered. The filtrate was dried in an oven at 50 °C. Residual water was vacuum evaporated at 100 °C and stored under an inert atmosphere of argon. Yield: 7.48 g (10.6 mmol, 82.8%). Colorless solid. Anal. Calc. for $(Bu_4P)_2SO_4{\cdot}5H_2O$ ($C_{32}H_{82}O_9P_2S$): C, 54.52; H, 11.72; S, 4.55. Found: C, 54.33; H, 11.47; S, 4.77. ICP Calc.: S, 4.55; P, 8.79. Found: S, 4.54; P, 8.89.

Synthesis of Mg[VO(edta)] (1-Mg). Compound **1**-Ba·6H_2O (1.20 g, 2.0 mmol) was dissolved in hot distilled water (10 mL; 80 °C) and treated with a solution of $MgSO_4{\cdot}7H_2O$ (429 mg, 2.00 mmol) in distilled water (5 mL), stirred for 30 min and filtered. The filtrate was overlayed with dioxane (5 mL) and stored at room temperature for 16 h to give blue crystals of the product. They were collected by filtration and dried in an oven at 50 °C. Yield: 956 mg (1.63 mmol, 81.6%). Blue crystals. Anal. Calc. for **1**-Mg·0.5(dioxane)·9H_2O ($C_{12}H_{34}MgN_2O_{19}V$): C, 24.61; H, 5.85; N, 4.78. Found: C, 24.52; H: 5.61; N, 4.53. ICP Calc.: V, 8.70. Found: V, 8.71. ESI-MS (H_2O), *m/z*, negative-ion: 177.5 (100%) $[M]^{2-}$, 186.5 $[M + H_2O]^{2-}$, 356 $[M + H]^-$. EPR (water): g_{iso} = 1.969, $|A_{iso}|$ = 10.40 mT. Single crystals of **1**-Mg·0.5(dioxane)·9H_2O suitable for XRD analysis were prepared by vapor diffusion of dioxane into the aqueous solution of **1**-Mg.

Synthesis of Cs_2[VO(edta)] (1-Cs). Compound **1-Ba**·6H_2O (0.60 g, 1.0 mmol) was dissolved in hot distilled water (10 mL; 80 °C) and treated with a solution of Cs_2SO_4 (362 mg, 1.00 mmol) in distilled water (5 mL), stirred for 30 min and filtered. The filtrate was left at room temperature in opened vessels for several days to give blue crystals of the product. They were collected by filtration and dried in an oven at 50 °C. Yield: 0.46 g (0.70 mmol, 70.0%). Blue crystals. Anal. Calc. for **1**-Cs·2H_2O ($C_{10}H_{16}Cs_2N_2O_{11}V$): C, 18.28; H, 2.45; N, 4.26. Found: C, 18.14; H, 2.23; N, 4.07. ICP Calc.: V, 7.75. Found: V, 7.69. ESI-MS (H_2O), *m/z*, positive-ion: 133 (100%) $[Cs]^+$, 754 $[M + 3 Cs]^+$; negative-ion: 177.5 $[M]^{2-}$, 186.5 $[M + H_2O]^{2-}$, 356 (100%) $[M + H]^-$, 488 $[Cs + M]^-$. EPR (water): g_{iso} = 1.969, $|A_{iso}|$ = 10.42 mT. Single crystals of **1**-Cs·2H_2O suitable for XRD analysis were prepared by slow evaporation of the aqueous solution of **1**-Cs.

Synthesis of Mg[VO(dcta)] (2-Mg). Compound **2**-Ba·6H_2O (1.00 g, 1.52 mmol) was dissolved in hot distilled water (40 mL; 80 °C), treated with a solution of $MgSO_4{\cdot}7H_2O$ (376 mg, 1.52 mmol) in distilled water (2 mL), stirred for 30 min and filtered. The filtrate was dried in a drying over at 60°C to a constant mass. Yield: 701 mg (1.29 mmol; 84.9%). Blue powder. Anal. Calc. for **2**-Mg·6H_2O ($C_{14}H_{30}MgN_2O_{15}V$): C, 24.63; H, 4.96; N, 5.75. Found: C, 24.48; H, 4.87; N, 5.51. ICP Calc.: V, 9.41. Found: V, 9.09. ESI-MS (H_2O), *m/z*, negative-ion: 205 $[M]^{2-}$, 214 $[M + H_2O]^{2-}$, 410 (100%) $[M + H]^-$, 421 $[2M + Mg]^{2-}$. EPR (water): g_{iso} = 1.969, $|A_{iso}|$ = 10.32 mT.

Synthesis of Cs_2[VO(dcta)] (2-Cs). Compound **2**-Ba·6H_2O (1.00 g, 1.52 mmol) was dissolved in hot distilled water (40 mL; 80 °C), treated with a solution of Cs_2SO_4 (552 mg, 1.52 mmol) in distilled water (2 mL), stirred for 30 min and filtered. The filtrate was dried in an over at 60°C to a constant mass. Yield: 871 mg (1.17 mmol, 77.0%). Blue powder. Anal. Calc. for **2**-Cs·4H_2O ($C_{14}H_{26}Cs_2N_2O_{13}V$): C, 22.51; H, 3.51; N, 3.75. Found: C, 22.38; H, 3.45; N, 3.52. ICP Calc.: V, 6.82. Found: V, 6.86. ESI-MS (H_2O), *m/z*, positive-ion: 133 (100%) $[Cs]^+$, 676 $[M + 2 Cs + H]^+$, 808 $[M + 3 Cs]^+$; negative-ion: 205 $[M]^{2-}$, 214 $[M + H_2O]^{2-}$, 410 (100%) $[M + H]^-$, 542 $[M + Cs]^-$. EPR (water): g_{iso} = 1.969, $|A_{iso}|$ = 10.31 mT.

Synthesis of $(Bu_4N)_2$[VO(dcta)] (2-Bu_4N). Compound **2**-Ba·6H_2O (520 mg, 0.79 mmol) was dissolved in hot distilled water (40 mL; 80 °C) and treated with a solution of $(Bu_4N)_2SO_4$·35H_2O (957 mg, 0.79 mmol) in distilled water (5 mL), stirred for 30 min and filtered. The filtrate was dried in a drying over at 60°C. The solid residue was dissolved in MeCN (20 mL), filtered and solvent was vacuum evaporated at 100 °C to a constant mass. The product was stored under an inert atmosphere of argon. Yield: 551 mg (0.41 mmol; 51.9%). Blue viscous liquid. Anal. Calc. for **2**-Bu_4N·25H_2O ($C_{46}H_{140}N_4O_{34}V$): C, 41.09; H, 10.50; N, 4.17. Found: C, 40.78; H, 10.63; N, 4.33. ICP Calc.: V, 3.79. Found: V, 3.67. ESI-MS (H_2O), *m/z*, positive-ion: 242 (100%) $[Bu_4N]^+$; negative-ion: 205 $[M]^{2-}$, 214 $[M + H_2O]^{2-}$, 410 (100%) $[M + H]^-$, 651 $[M + Bu_4N]^-$. EPR (water): g_{iso} = 1.968, $|A_{iso}|$ = 10.28 mT; EPR (MeCN): g_{iso} = 1.969, $|A_{iso}|$ = 10.41 mT.

Synthesis of $(Bu_4P)_2$[VO(dcta)] (2-Bu_4P). Compound **2**-Ba·6H_2O (510 mg, 0.78 mmol) was dissolved in hot distilled water (20 mL; 80 °C), treated with a solution of $(Bu_4P)_2SO_4$·5H_2O (550 mg, 0.78 mmol) in distilled water (5 mL), stirred for 30 min and filtered. The filtrate was dried in a drying over at 60°C. The solid residue was dissolved in MeCN (20 mL), filtered and the solvent was vacuum evaporated to a constant mass. The product was stored under an inert atmosphere of argon. Yield: 593 mg (0.63 mmol; 80.4%). Blue viscous liquid. Anal. Calc. for 2-Bu_4P·H_2O ($C_{46}H_{92}N_2O_{10}P_2V$): C, 58.40; H, 9.80; N, 2.96. Found: C, 58.77; H, 9.71; N, 2.67. ICP Calc.: V, 5.38; P, 6.55. Found: V, 5.53; P, 6.55. ESI-MS (H_2O), *m/z*, positive-ion: 259 (100%) $[Bu_4P]^+$; negative-ion: 205 $[M]^{2-}$, 410 (100%) $[M + H]^-$, 668 $[M + Bu_4P]^-$. EPR (water): g_{iso} = 1.969, $|A_{iso}|$ = 10.29 mT; EPR (MeCN): g_{iso} = 1.968, $|A_{iso}|$ = 10.40 mT.

Synthesis of Mg[VO(oedta)]$_2$ (3-Mg). Compound **3**-Ba·6H_2O (235 mg, 0.22 mmol) was dissolved in hot distilled water (3 mL; 80 °C) and treated with a solution of $MgSO_4$·7H_2O (54.5 mg, 0.22 mmol) in distilled water (1 mL), stirred for 30 min and filtered. The filtrate was treated with propan-2-ol (4 mL) and stored at 10 °C for 16 h to give blue crystals of the product. The crystals were collected by filtration and dried in an oven at 50 °C. Yield: 155 mg (0.16 mmol; 73.9 %). Blue powder. Anal. Calc. for **3**-Mg·6H_2O ($C_{32}H_{66}MgN_4O_{20}V_2$): C, 40.33; H, 6.98; N, 5.88. Found: C, 39.99; H, 7.19; N, 5.63. ICP Calc.: V, 10.69. Found: V, 10.55. ESI-MS (H_2O), *m/z*, negative-ion: 205 $[M]^{2-}$, 214 $[M + H_2O]^{2-}$, 410 (100%) $[M + H]^-$, 421 $[2M + Mg]^{2-}$. EPR (water): g_{iso} = 1.969, $|A_{iso}|$ = 10.32 mT. ESI-MS (H_2O), *m/z*, negative-ion: 410 (100%) $[M]^-$. EPR (water): g_{iso} = 1.970, $|A_{iso}|$ = 10.35 mT.

Synthesis of Cs[VO(oedta)] (3-Cs). Compound **3**-Ba·6H_2O (245 mg, 0.23 mmol) was dissolved in hot distilled water (3 mL; 80 °C) and treated with a solution of Cs_2SO_4 (84.9 mg, 0.23 mmol) in distilled water (2 mL), stirred for 30 min. Slow diffusion of acetone vapors in the closed vessels led to crystallization of the product. The product was collected by filtration and dried in an oven at 50 °C. Yield: 199 mg (0.31 mmol; 67.4%). Blue crystals. Anal. Calc. for **3**-Cs·3H_2O·acetone ($C_{19}H_{39}CsN_2O_{11}V$): C, 34.82; H, 6.00; N, 4.27. Found: C, 34.54; H, 5.82; N, 4.02. ICP Calc.: V, 7.82. Found: V, 7.68. ESI-MS (H_2O), *m/z*, positive-

ion: 133 $[Cs]^+$, 544 $[M + Cs + H]^+$, 676 (100%) $[M + 2 Cs]^+$; negative-ion: 410 (100%) $[M]^-$. EPR (water): g_{iso} = 1.969, $|A_{iso}|$ = 10.35 mT.

Synthesis of $(Bu_4N)[VO(oedta)]$ (3-Bu_4N). Compound **3**-Ba·$6H_2O$ (2.00 g, 1.88 mmol) was dissolved in hot distilled water (20 mL; 80 °C) and treated with a solution of $(Bu_4N)_2SO_4 \cdot 35H_2O$ (2.28 g, 1.88 mmol) in distilled water (6 mL), stirred for 30 min and filtered. The filtrate was vacuum dried at 100 °C to a constant mass. The product was stored under an inert atmosphere of argon. Yield: 2.42 g (3.34 mmol; 88.8%). Blue solid. Anal. Calc. for **3**-$Bu_4N \cdot 4H_2O$ ($C_{32}H_{71}N_3O_{11}V$): C, 53.02; H, 9.87, N: 5.80. Found: C, 53.39; H, 9.58; N, 5.87. ICP Calc.: V, 7.03. Found: V, 6.92. ESI-MS (H_2O), *m/z*, positive-ion: 242 (100%) $[Bu_4N]^+$; negative-ion: 410 (100%) $[M]^-$. EPR (water): g_{iso} = 1.969, $|A_{iso}|$ = 10.39 mT; EPR (MeCN): g_{iso} = 1.969, $|A_{iso}|$ = 10.55 mT.

Synthesis of $(Bu_4P)[VO(oedta)]$ (3-Bu_4P). Compound **3**-Ba·$6H_2O$ (1.01 g, 0.95 mmol) was dissolved in warm distilled water (10 mL; 60 °C) and treated with a solution of $(Bu_4P)_2SO_4 \cdot 5H_2O$ (670 mg, 0.95 mmol) in distilled water (5 mL), stirred for 30 min and filtered. The filtrate was vacuum dried at 100 °C to a constant mass. The product was stored under an inert atmosphere of argon. Yield: 1.40 g (1.52 mmol; 80.0%). Blue viscous liquid. Anal. Calc. for **3**-$Bu_4P \cdot 14H_2O$ ($C_{32}H_{91}PN_2O_{21}V$): C, 41.69; H, 9.95; N, 3.04. Found: C, 41.36; H, 9.64; N, 2.83. ICP Calc.: V, 5.53; P, 3.36. Found: V, 5.60; P, 3.50. ESI-MS (H_2O), *m/z*, positive-ion: 259 (100%) $[Bu_4P]^+$; negative-ion: 410 (100%) $[M]^-$. EPR (water): g_{iso} = 1.969, $|A_{iso}|$ = 10.37 mT; EPR (MeCN): g_{iso} = 1.969, $|A_{iso}|$ = 10.53 mT.

Synthesis of $Mg[VO(heedta)]_2$ (4-Mg). Compound **4**-Ba·$3H_2O$ (534 mg, 0.61 mmol) was dissolved in hot distilled water (20 mL; 80 °C), treated with a solution of $MgSO_4 \cdot 7H_2O$ (150 mg, 0.61 mmol) in distilled water (3 mL), stirred for 30 min and filtered. The filtrate was concentrated in vacuum to a volume of 3 mL and treated with BuOH (2 mL). Slow diffusion of acetone vapors in closed vessels led to crystallization of the product. Yield: 474 mg (0.56 mmol; 91.8%). Blue crystals. Anal. Calc. for **4**-Mg·$8H_2O$ ($C_{20}H_{46}Mg\ N_4O_{24}V_2$): C, 28.17; H, 5.44; N, 6.57. Found: C, 28.51; H, 5.54; N, 6.43. ICP Calc.: V, 11.95. Found: V, 11.86. ESI-MS (H_2O), *m/z*, negative-ion: 342 (100%) $[M]^-$. EPR (water): g_{iso} = 1.970, $|A_{iso}|$ = 10.42 mT. Large crystals of **4**-Mg·$8H_2O$ suitable for XRD analysis were prepared by slow diffusion of acetone vapors into an aqueous solution of **4**-Mg overlayered by BuOH.

Synthesis of Cs[VO(heedta)]·H_2O (4-Cs·H_2O). Compound **4**-Ba·$3H_2O$ (534 mg, 0.61 mmol) was dissolved in hot distilled water (20 mL; 80 °C), treated with a solution of Cs_2SO_4 (220 mg, 0.61 mmol) in distilled water (3 mL), stirred for 30 min and filtered. The filtrate was concentrated in vacuum to a volume of 3 mL and treated with BuOH (2 mL). Slow diffusion of acetone vapors in the closed vessels led to crystallization of the product. Yield: 523 mg (1.06 mmol; 86.9%). Anal. Calc. for **4**-Cs·H_2O ($C_{10}H_{17}CsN_2O_9V$): C, 24.36; H, 3.47; N, 5.68. Found: C, 24.12; H, 3.21; N, 5.43. ICP Calc.: V, 10.33. Found: V, 10.06. ESI-MS (H_2O), *m/z*, positive-ion: 133 $[Cs]^+$, 608 (100%) $[M + 2 Cs]^+$; negative-ion: 342 (100%) $[M]^-$. EPR (water): g_{iso} = 1.969, $|A_{iso}|$ = 10.41 mT. Large crystals of **4**-Cs·H_2O suitable for XRD analysis were prepared by slow diffusion of acetone vapors into an aqueous solution of **4**-Cs overlayered by BuOH.

Synthesis of $(Bu_4N)[VO(heedta)]$ (4-Bu_4N). Compound **4**-Ba·$3H_2O$ (517 mg, 0.59 mmol) was dissolved in hot distilled water (20 mL; 80 °C), treated with a solution of $(Bu_4N)_2SO_4 \cdot 4H_2O$ (382 mg, 0.59 mmol) in distilled water (5 mL), stirred for 30 min and filtered. The filtrate was dried in an over at 60°C. The solid

residue was dissolved MeCN (20 mL), filtered and the solvent was vacuum evaporated at 100 °C to a constant mass. The product was stored under an inert atmosphere of argon. Yield: 289 mg (0.34 mmol; 57.3%). Blue viscous liquid. Anal. Calc. for **4**-$Bu_4N \cdot 15H_2O$ ($C_{26}H_{81}N_3O_{23}V$): C, 36.52; H, 9.55; N, 4.92. Found: C, 36.41; H, 9.21; N, 4.63. ICP Calc.: V, 5.96. Found: V, 5.77. ESI-MS (H_2O), *m/z*, positive-ion: 242 (100%) $[Bu_4N]^+$; negative-ion: 342 (100%) $[M]^-$. EPR (water): g_{iso} = 1.968, $|A_{iso}|$ = 10.43 mT; EPR (MeCN): g_{iso} = 1.968, $|A_{iso}|$ = 10.51 mT.

Synthesis of (Bu_4P)[VO(heedta)] (4-Bu_4P). Compound **4**-Ba·$3H_2O$ (578 mg, 0.66 mmol) was dissolved in hot distilled water (20 mL; 80 °C), treated with a solution of $(Bu_4P)_2SO_4 \cdot 5H_2O$ (468 mg, 0.66 mmol) in distilled water (5 mL), stirred for 30 min and filtered. The filtrate was dried in an over at 60°C. The solid residue was dissolved MeCN (20 mL), filtered and the solvent was vacuum evaporated at 100 °C. The product was stored under an inert atmosphere of argon. Yield: 328 mg (0.53 mmol; 80.2%). Blue viscous liquid. Anal. Calc. for **4**-$Bu_4P \cdot H_2O$ ($C_{26}H_{53}N_2O_9PV$): C, 50.40; H, 8.62; N, 4.52. Found: C, 50.15; H, 8.46; N, 4.38. ICP Calc.: V, 8.22; P, 5.00. Found: V, 8.18; P, 5.33. ESI-MS (H_2O), *m/z*, positive-ion: 259 (100%) $[Bu_4P]^+$; negative-ion: 342 (100%) $[M]^-$. EPR (water): g_{iso} = 1.969, $|A_{iso}|$ = 10.43 mT; EPR (MeCN): g_{iso} = 1.968, $|A_{iso}|$ = 10.52 mT.

Table S1: EPR parameters of fluid solution spectra measured at room temperature.

	Solvent	$\lvert A_{iso}\rvert$ (mT)	g_{iso}
$[VO(H_2O)_5]SO_4$	H_2O	11.61	1.956
**1**-Ba	H_2O	10.41	1.970
**1**-Mg	H_2O	10.40	1.969
**1**-Cs	H_2O	10.42	1.969
**1**-Bu_4N	H_2O	10.43	1.969
**1**-Bmim	H_2O	10.40	1.968
**1**-Bu_4P	H_2O	10.41	1.969
**1**-Bu_4N	MeCN	10.48	1.967
**1**-Bmim	MeCN	10.47	1.968
**1**-Bu_4P	MeCN	10.48	1.969
**2**-Ba	H_2O	10.29	1.968
**2**-Mg	H_2O	10.32	1.969
**2**-Cs	H_2O	10.31	1.969
**2**-Bu_4N	H_2O	10.28	1.968
**2**-Bmim	H_2O	10.32	1.969
**2**-Bu_4P	H_2O	10.29	1.969
**2**-Bu_4N	MeCN	10.41	1.969
**2**-Bmim	MeCN	10.36	1.969
**2**-Bu_4P	MeCN	10.40	1.968
**3**-Ba	H_2O	10.34	1.970
**3**-Mg	H_2O	10.35	1.970
**3**-Cs	H_2O	10.35	1.969
**3**-Bu_4N	H_2O	10.39	1.969
**3**-Bmim	H_2O	10.34	1.969
**3**-Bu_4P	H_2O	10.37	1.969
**3**-Bu_4N	MeCN	10.55	1.969
**3**-Bmim	MeCN	10.53	1.968
**3**-Bu_4P	MeCN	10.53	1.969
**4**-Ba	H_2O	10.41	1.969
**4**-Mg	H_2O	10.42	1.970
**4**-Cs	H_2O	10.41	1.969
**4**-Bu_4N	H_2O	10.43	1.968
**4**-Bmim	H_2O	10.41	1.969
**4**-Bu_4P	H_2O	10.43	1.969
**4**-Bu_4N	MeCN	10.51	1.968
**4**-Bmim	MeCN	10.51	1.968
**4**-Bu_4P	MeCN	10.52	1.968

Table S2: EPR parameters of frozen methanol/DMSO measured at –150 °C.

	$A_{\parallel}$ (mT)	$g_{\parallel}$	$A_{\perp}$ (mT)	$g_{\perp}$	$\|A_{iso}\|$ [mT] calculated	g_{iso} calculated
**1**-Bu_4N	18.65	1.944	6.49	1.978	10.55	1.967
**1**-Bmim	18.62	1.944	6.49	1.978	10.53	1.967
**1**-Bu_4P	18.64	1.944	6.49	1.978	10.54	1.967
**2**-Bu_4N	18.53	1.944	6.48	1.977	10.50	1.966
**2**-Bmim	18.46	1.944	6.45	1.977	10.45	1.966
**2**-Bu_4P	18.51	1.944	6.47	1.976	10.48	1.966
**3**-Bu_4N	18.62	1.944	6.49	1.979	10.53	1.967
**3**-Bmim	18.61	1.944	6.48	1.978	10.53	1.967
**3**-Bu_4P	18.59	1.945	6.46	1.978	10.51	1.967
**4**-Bu_4N	18.63	1.944	6.50	1.978	10.54	1.967
**4**-Bmim	18.61	1.944	6.50	1.977	10.54	1.966
**4**-Bu_4P	18.63	1.944	6.49	1.978	10.53	1.967

Table S3: Selected bond lengths (Å) and angles (°) of vanadyl complexes.

	**1**-Ba·6H_2O	**1**-Mg ·9H_2O ·0.5($C_4H_8O_2$)	**1**-Cs·2H_2O	**2**-Ba·6H_2O	**3**-Ba ·5H_2O ·2^{i}PrOH (a)	**3**-Ba ·5H_2O ·2^{i}PrOH (b)
V-O1	1.612(2)	1.607(1)	1.617(4)	1.622(3)	1.620(6)	1.626(5)
V-O2	1.998(2)	2.022(1)	1.997(3)	2.128(3)	1.996(4)	2.008(4)
V-O4	2.018(2)	2.020(1)	2.005(3)	2.004(3)	2.016(4)	2.007(4)
V-O6	2.008(2)	1.998(1)	1.978(3)	2.153(4)	1.985(5)	1.990(5)
V-O8	-	-	-	2.015(3)	-	-
V-N1	2.308(3)	2.286(1)	2.293(4)	2.429(4)	2.282(7)	2.280(5)
V-N2	2.161(3)	2.141(1)	2.172(4)	2.413(3)	2.145(5)	2.148(6)
O1-V-O2	104.3(1)	102.86(6)	103.0(1)	81.5(1)	103.0(2)	102.0(2)
O1-V-O4	95.3(1)	97.77(6)	94.8(1)	97.3(2)	96.6(2)	96.7(2)
O1-V-O6	104.1(1)	101.21(6)	102.5(1)	79.9(1)	103.7(2)	103.3(2)
O1-V-O8	-	-	-	99.1(2)	-	-
O1-V-N1	172.2(1)	173.85(6)	171.0(1)	144.8(1)	172.5(2)	172.6(2)
O1-V-N2	101.5(1)	100.91(6)	103.1(1)	144.3(1)	101.8(2)	103(2)
N1-V-N2	80.26(9)	81.84(5)	80.1(1)	70.9(1)	88.8(2)	80.4(2)

Table S4: Selected bond lengths (Å) and angles (°) of vanadyl complexes.

	**4**-Ba ·3H_2O (a)	**4**-Ba ·3H_2O (b)	**4**-Ba ·3H_2O (c)	**4**-Ba ·3H_2O (d)	**4**-Mg ·8H_2O	**4**-Cs ·H_2O (a)	**4**-Cs ·H_2O (b)
V-O1	1.606(7)	1.610(6)	1.608(6)	1.623(6)	1.617(3)	1.613(1)	1.614(1)
V-O2	2.010(6)	2.009(6)	2.027(6)	1.999(6)	2.009(2)	2.012(1)	2.019(1)
V-O4	2.005(6)	2.044(6)	2.025(6)	2.045(6)	2.029(3)	2.012(1)	2.018(1)
V-O6	1.990(6)	1.977(6)	1.959(6)	1.964(6)	1.981(3)	1.983(1)	1.974(1)
V-O8	-	-	-	-	-	-	-
V-N1	2.290(7)	2.304(7)	2.284(8)	2.263(7)	2.289(3)	2.311(2)	2.315(2)
V-N2	2.154(7)	2.139(7)	2.156(6)	2.147(7)	2.153(3)	2.153(1)	2.154(1)
O1-V-O2	104.9(3)	104.2(3)	104.1(3)	106.7(3)	101.8(1)	104.14(6)	104.58(6)
O1-V-O4	93.7(3)	96.1(3)	91.0(3)	89.2(3)	95.2(1)	96.26(6)	94.41(6)
O1-V-O6	104.5(3)	103.4(3)	106.5(3)	106.9(3)	104.7(1)	103.03(6)	104.73(6)
O1-V-O8	-	-	-	-	-	-	-
O1-V-N1	171.0(3)	171.7(3)	168.0(3)	166.4(3)	171.1(1)	173.01(6)	170.80(6)
O1-V-N2	100.2(3)	102.2(3)	103.3(3)	99.7(3)	103.0(1)	101.15(6)	102.66(6)
N1-V-N2	80.7(2)	80.7(2)	80.4(3)	80.6(3)	81.0(1)	80.34(5)	79.72(5)

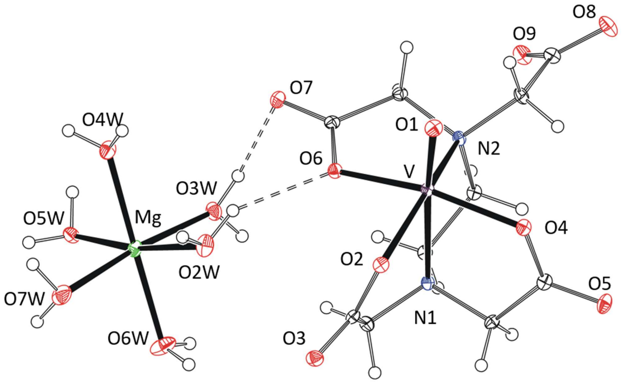

Figure S1: XRD structure of **1**-Mg·9H_2O·0.5(dioxane). Thermal ellipsoids are set to 30% probability. Free solvent molecules are omitted for clarity.

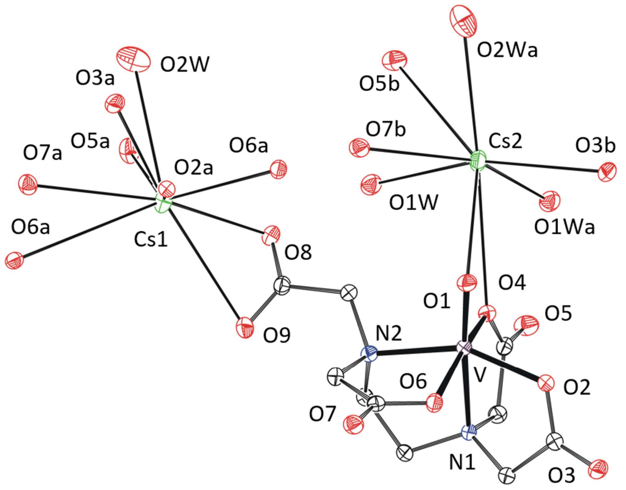

Figure S2. XRD structure of **1**-Cs·2H_2O. Thermal ellipsoids are set to 30% probability. Hydrogen atoms positional disorders on cesium atoms are omitted for clarity.

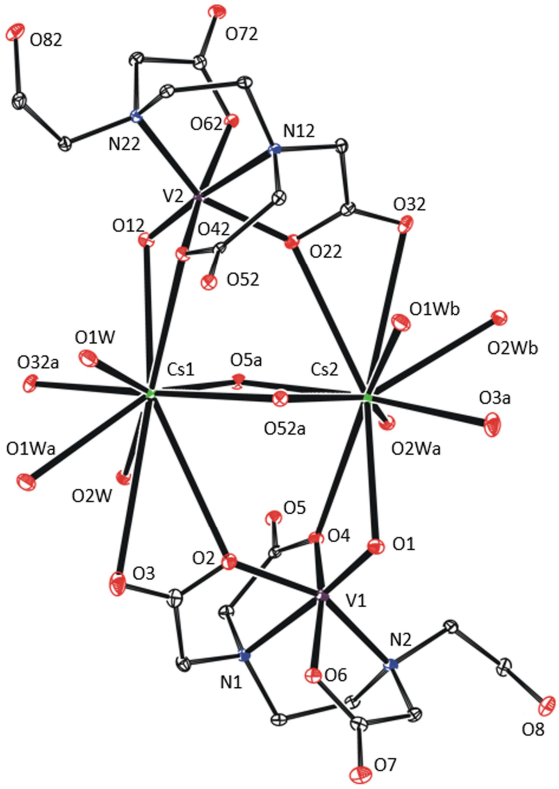

Figure S3. XRD structure of **4**-Cs·H_2O. Thermal ellipsoids are set to 30% probability.

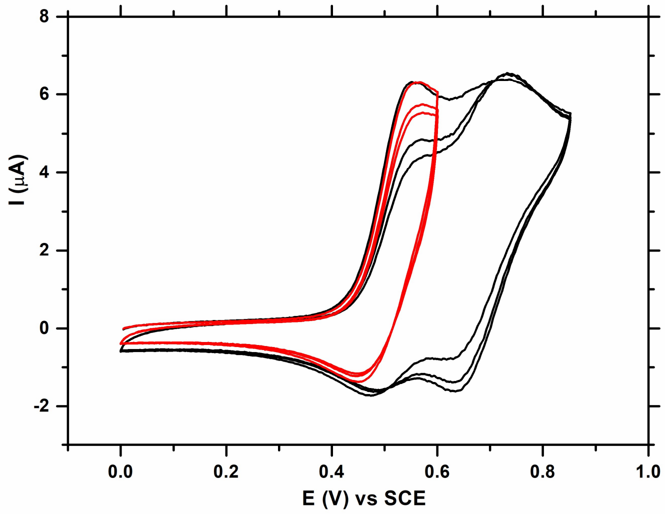

Figure S4: CV curve of oxidation of **1**-Bmim (left) at glassy carbon electrode in MeCN solution containing 0.1 M Bu_4NPF_6; v = 100 mV·s^{-1}.

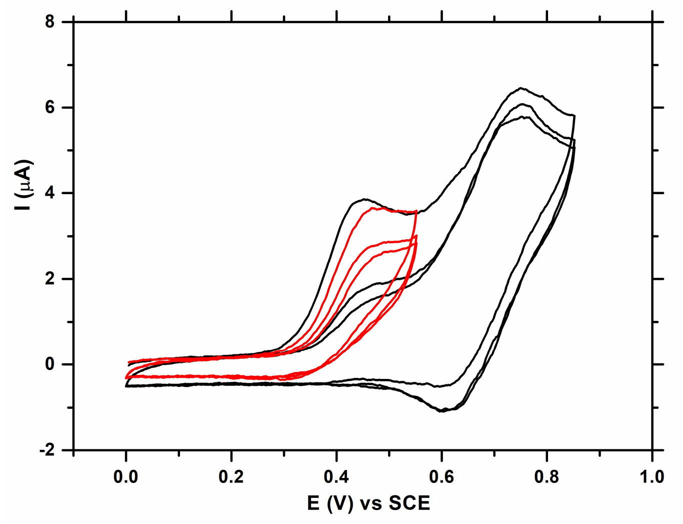

Figure S5: CV curve of oxidation of **2**-Bmim (left) at glassy carbon electrode in MeCN solution containing 0.1 M Bu_4NPF_6; v = 100 mV·s^{-1}.

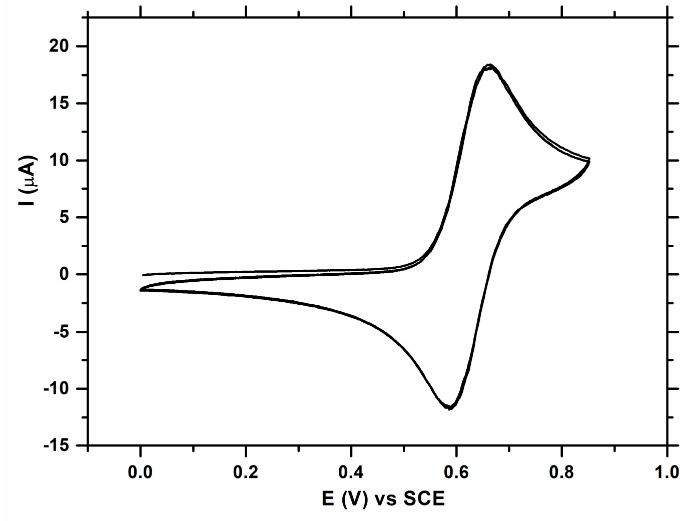

Figure S6: CV curve of oxidation of **3**-Bmim (left) at glassy carbon electrode in MeCN solution containing 0.1 M Bu_4NPF_6; v = 100 mV·s^{-1}.

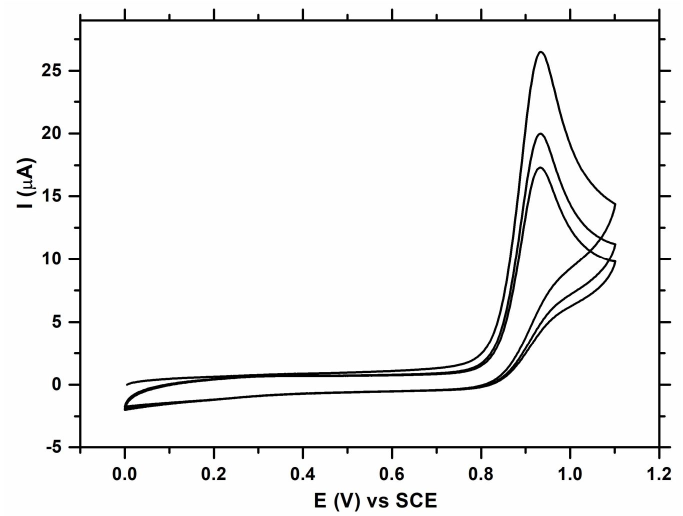

Figure S7: CV curve of oxidation of **4**-Bmim (left) at glassy carbon electrode in MeCN solution containing 0.1 M Bu_4NPF_6; v = 100 mV·s^{-1}.

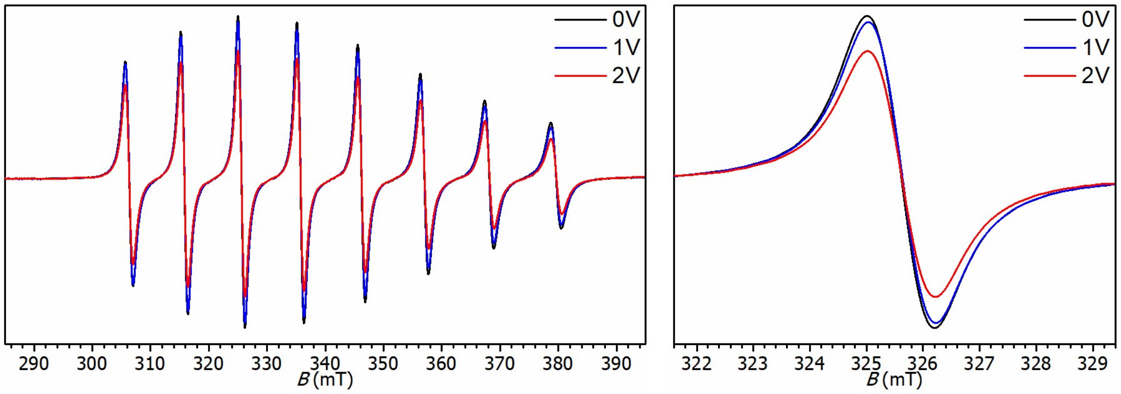

Figure S8: EPR spectra of the MeCN solution of **1**-Bmim (left) at a constant potential of platinum gauze and magnification of their third bands (right). Black line: reference spectrum without a potential collected prior to a voltametric experiment. Blue line: spectrum collected at +1 V vs. SCE (96% of original intensity). Red line: spectrum collected at +2 V vs. SCE (79% of original intensity).

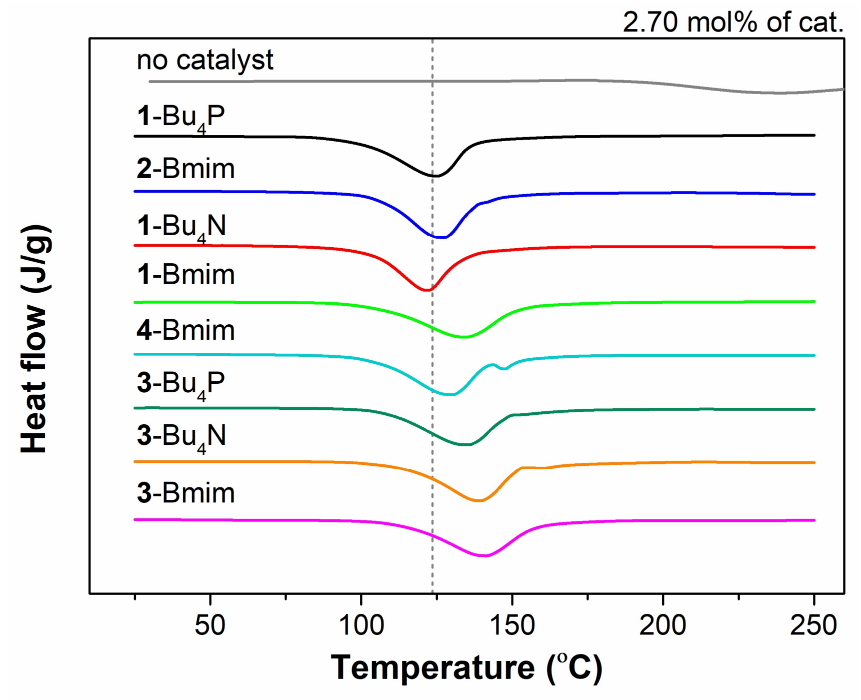

Figure S9: Dynamic DSC curves of reactive mixtures of DGEBA/MHHPA with 2.70 mol% of catalyst.

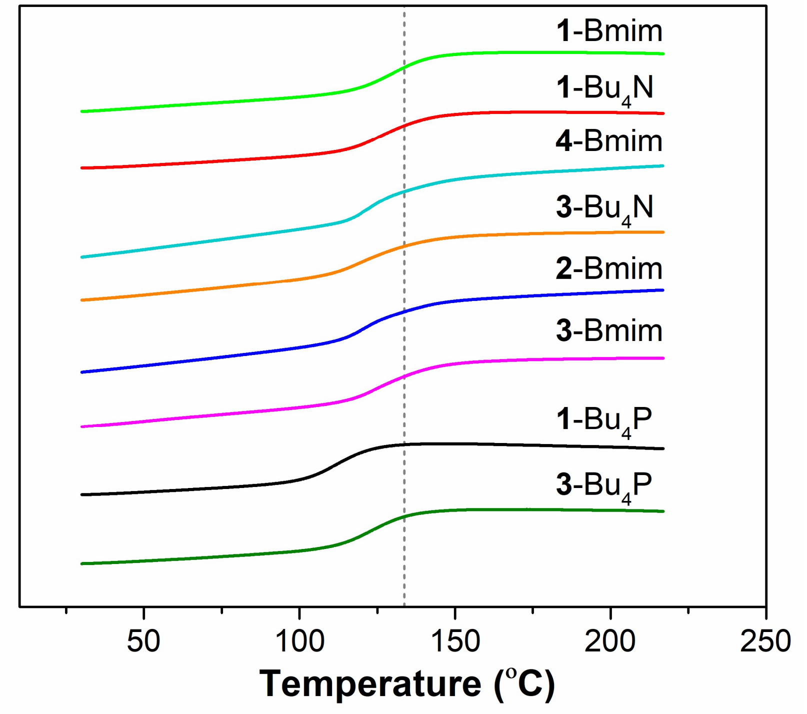

Figure S10: The second DSC heating run (10 °C/min) after the dynamic DSC curing showing the glass transition temperature (T_g) of the cured DGEBA/MHHPA networks with 2.70 mol% of catalyst.

Monitoring of the epoxy-anhydride copolymerization.

Isothermal differential scanning calorimetry (DSC) measurements of the reactive mixtures were performed using a DSC calorimeter (Q 2000, TA Instruments) calibrated for indium. Samples (5−15 mg) were hermetically sealed in Tzero aluminum pans with a pinhole and measured under a nitrogen flow of 50 mL/min. The conversion curves (Conversion vs time) were obtained by measuring the reactive mixture in DSC using dynamic and isothermal runs. Additional ramp DSC run of 10 °C /min was performed to measure the residual reaction heat (ΔH_{res}) after isothermal curing. The conversion of epoxy groups (α) was calculated from the DSC data according to equation (S1):

$$\alpha(t)_{\mathrm{DSC}} = \frac{\int_0^t \frac{dH}{dt} dt}{\Delta H_{total}} + \left(1 - \frac{\Delta H_{res} + \Delta H_{iso}}{\Delta H_{total}}\right) \tag{S1}$$

where dH/dt is the instantaneous time derivative of the heat during the dynamic or isothermal run, ΔH_{total} is the total enthalpy of the reaction determined from the dynamic DSC runs and ΔH_{iso} is the heat of reaction measured from the isothermal DSC scans. Then, the epoxy-anhydride cross-linking was fitted to a Kamal-Sourour model (equation S2):

$$\frac{d\alpha}{dt} = (k_1 + k_2\alpha^m)(1 - \alpha)^n \tag{S2}$$

where k_1 and k_2 are rate constants for the non-catalytic and the autocatalytic reactions respectively. Parameters m and n are partial reaction orders. A numerical integration using the 4th order Runge-Kutta method was used to calculate model predicted α_{sim} values for each tested temperature. These values were then compared to experimentally found α_{exp} (from isothermal DSC) using the ordinary least squares (OLS) error:

$$OLS = \sum_i [\alpha_{exp} - \alpha_{sim}]_i^2 \tag{S3}$$

This OLS criterion was then minimized using the GRG algorithm (Microsoft Excel). By this procedure, the reaction rates of non-catalyzed (k_1) and autocatalyzed (k_2) reactions were determined for each temperature. Arrhenius equation was used for the determination of the activation energy of the reactions:

$$k = A \exp\left(\frac{E_a}{RT}\right) \tag{S4}$$

where A is the pre-exponential factor, E_a is the activation energy, and R is the universal gas constant.

Near-infrared spectra (NIR) were acquired using a Nicolet iS50 FTIR spectrometer (Thermo Fisher Scientific) in transmission mode using a HT-32 heating cell and single-use homemade cuvettes made of two glass slides and acrylate tape as a spacer to adjust inner diameter (ID = 1 mm). The isothermal runs were carried out at 140 °C for 2 h and then dynamically heated (5 °C/ min) to 300 °C to reach full conversion. The spectra were collected every 20 s (32 scans per spectrum, a data spacing of 1 cm^{-1}). The collected spectra were integrated using a fixed two-point baseline in the regions of 4502−4568, 4803−4867, 5100−5190, 5210−5272, and 6800−7132 cm^{-1} to monitor epoxy, anhydride, ester, moisture, and hydroxy groups, respectively. The integral intensities obtained were calibrated based on

the intensity of the integrated reference band in the region of 4592–4652 cm^{-1}. Conversions of epoxy, anhydride, ester, moisture, and hydroxy groups are defined as follows (equations S1–S5):

$$\alpha(t)_{Epoxy} = 1 - \frac{A_{4530}(t)}{A_{5430}(t=0)} / \frac{A_{4622}(t)}{A_{4622}(t=0)} \tag{S5}$$

$$\alpha(t)_{Anhydride} = 1 - \frac{A_{4828}(t)}{A_{4828}(t=0)} / \frac{A_{4622}(t)}{A_{4622}(t=0)} \tag{S6}$$

$$\alpha(t)_{Ester} = \frac{A_{5158}(t)}{A_{5158}(max)} / \frac{A_{4622}(t)}{A_{4622}(t=0)} \tag{S7}$$

$$\alpha(t)_{Moisture} = 1 - \frac{A_{5252}(t)}{A_{5252}(t=0)} / \frac{A_{4622}(t)}{A_{4622}(t=0)} \tag{S8}$$

$$\alpha(t)_{Hydroxy} = \frac{A_{6985;7030}(t)}{A_{6985;7030}(max)} / \frac{A_{4622}(t)}{A_{4622}(t=0)} \tag{S9}$$

Additional characterizations

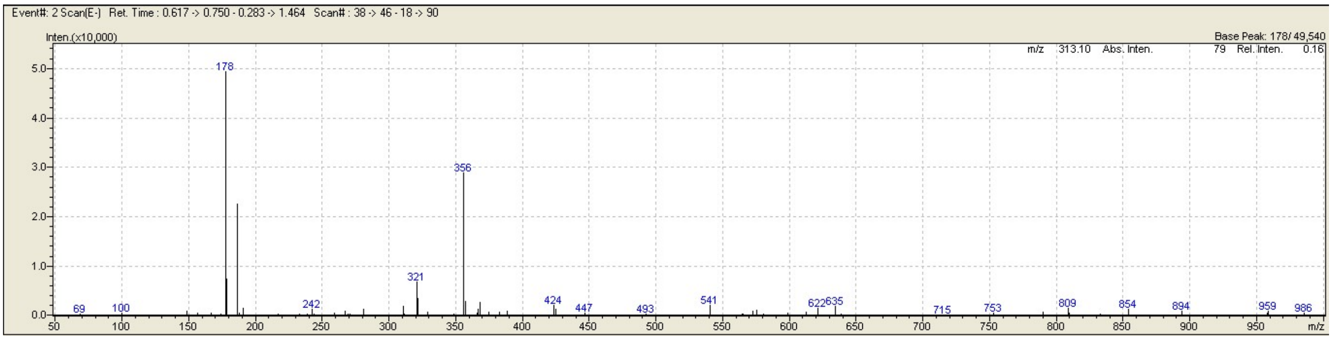

Figure S11: Negative-ion ESI-MS spectrum of **1**-Ba in water.

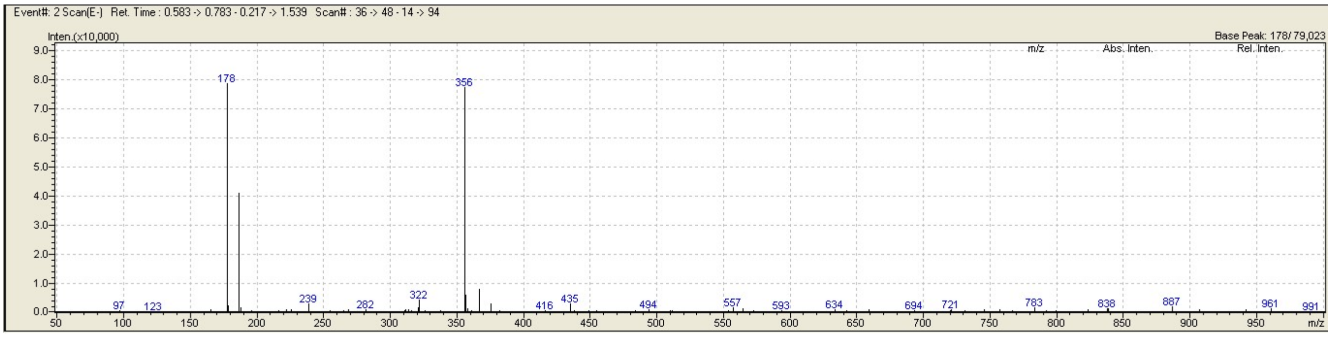

Figure S12: Negative-ion ESI-MS spectrum of **1**-Mg in water.

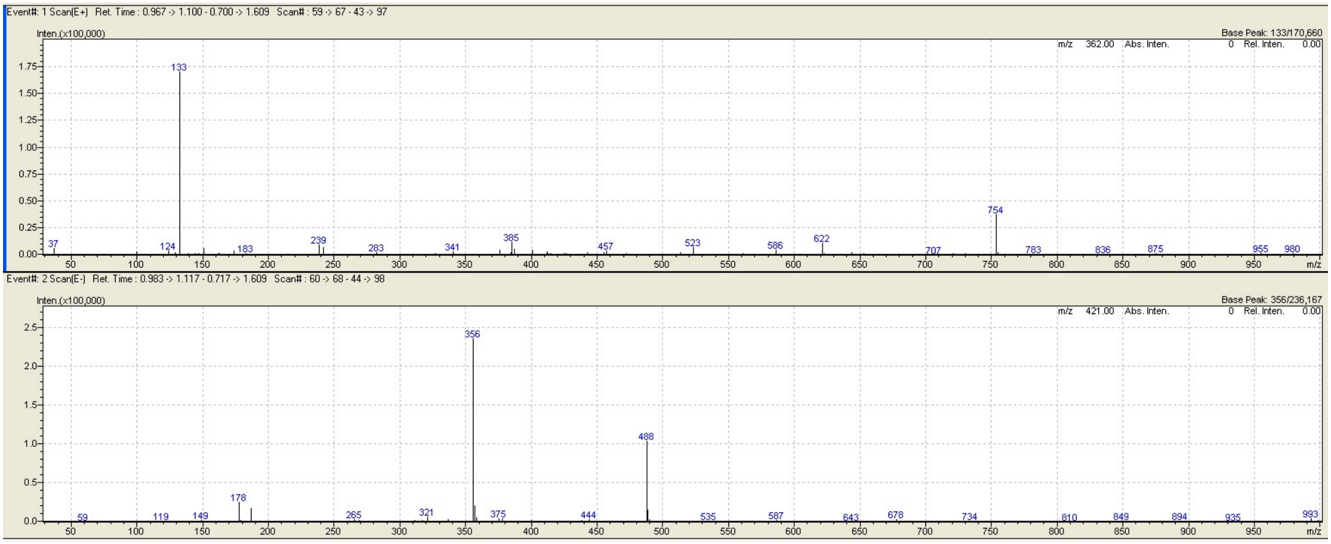

Figure S13: ESI-MS spectra of **1**-Cs in water. Positive-ion mode (top), negative-ion mode (bottom).

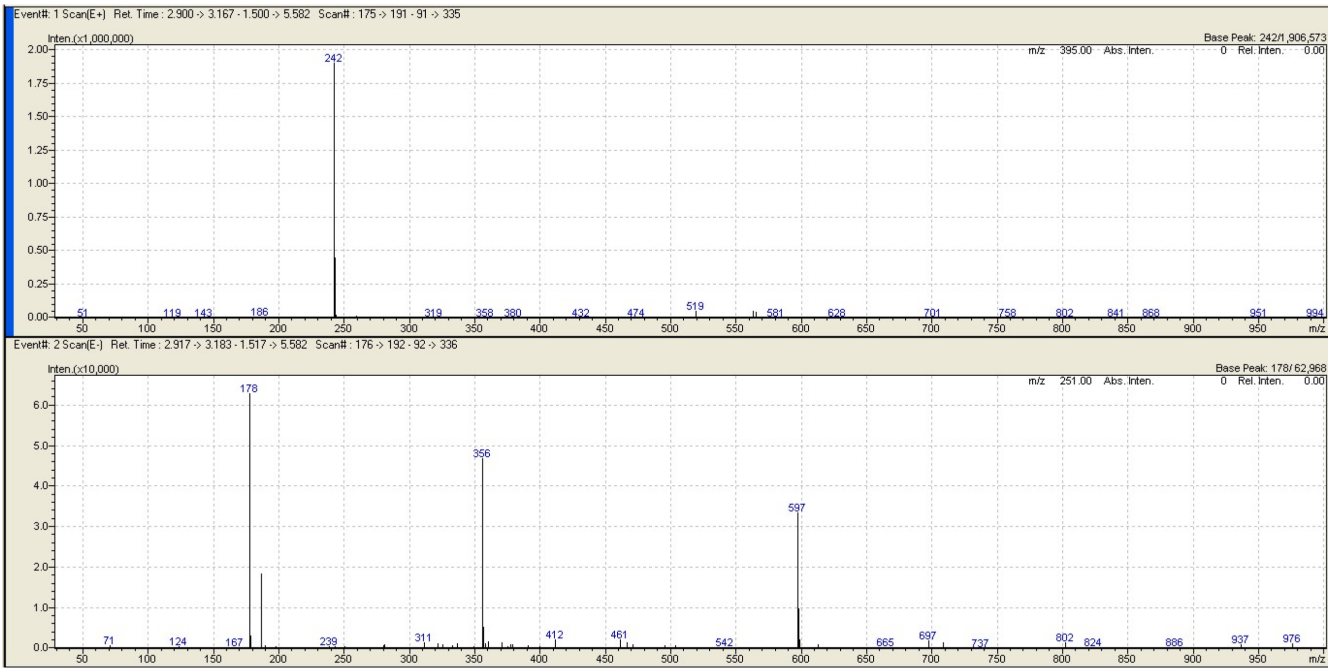

Figure S14: ESI-MS spectra of **1**-Bu_4N in water. Positive-ion mode (top), negative-ion mode (bottom).

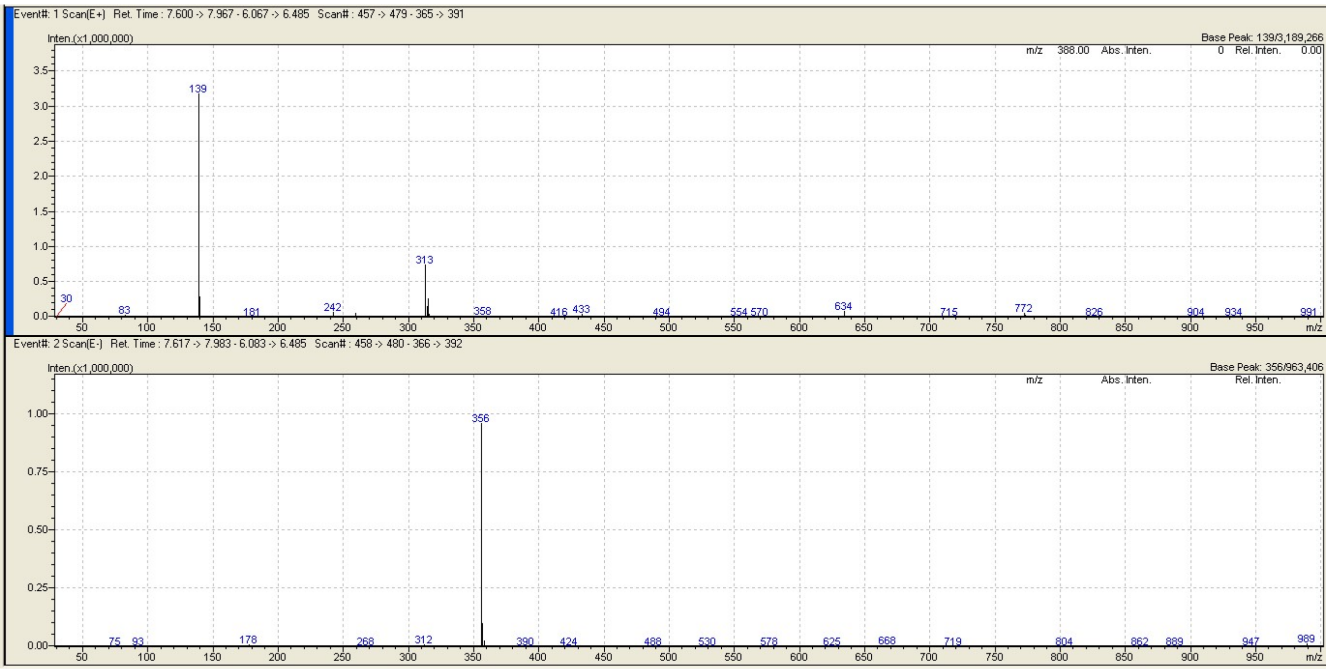

Figure S15: ESI-MS spectra of **1**-Bmim in water. Positive-ion mode (top), negative-ion mode (bottom).

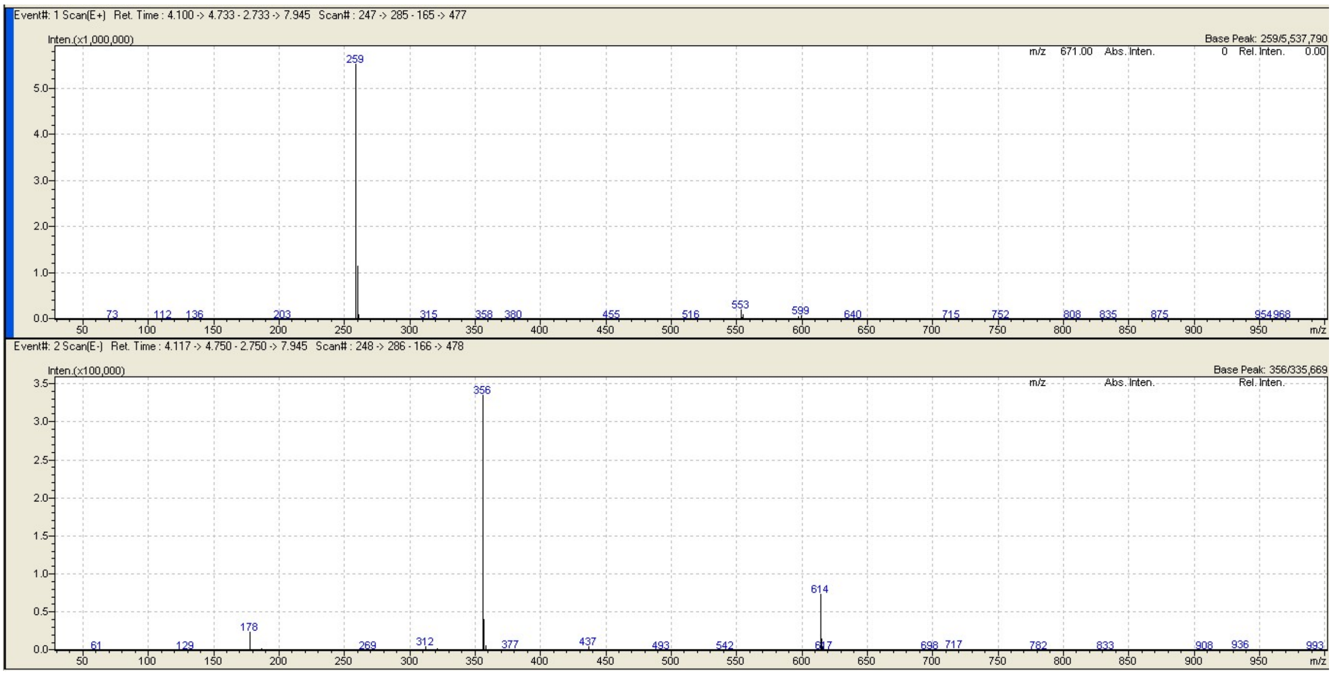

Figure S16: ESI-MS spectra of **1**-Bu_4P in water. Positive-ion mode (top), negative-ion mode (bottom).

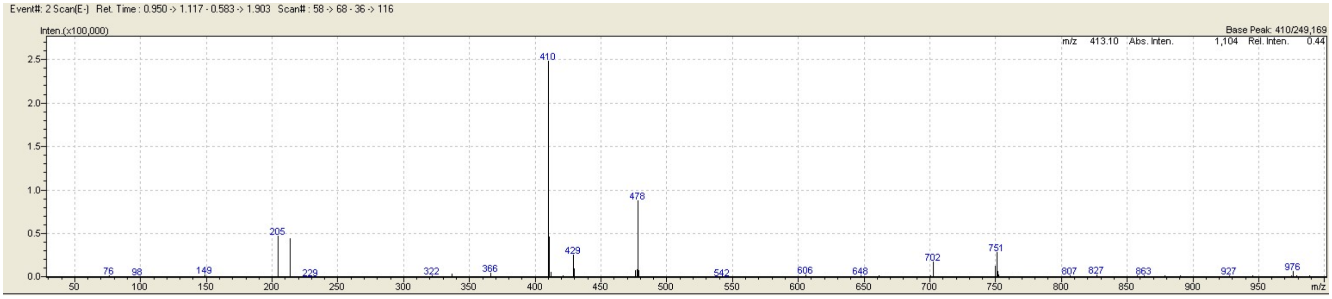

Figure S17: Negative-ion ESI-MS spectrum of **2**-Ba in water.

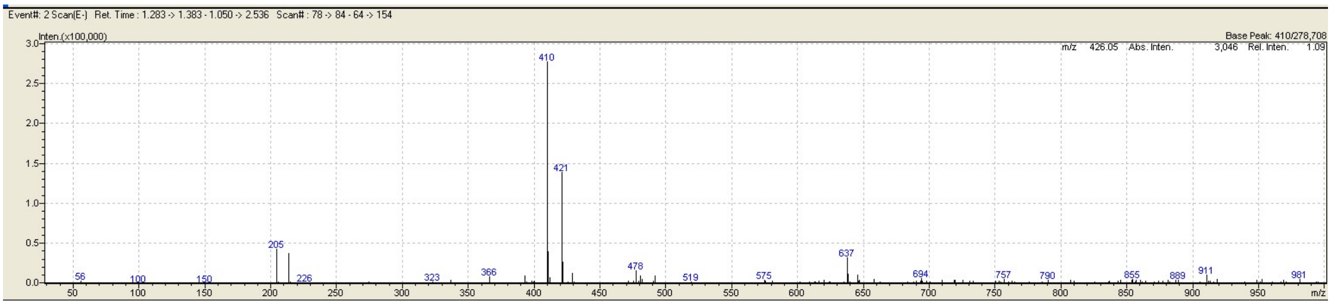

Figure S18: Negative-ion ESI-MS spectrum of **2**-Mg in water.

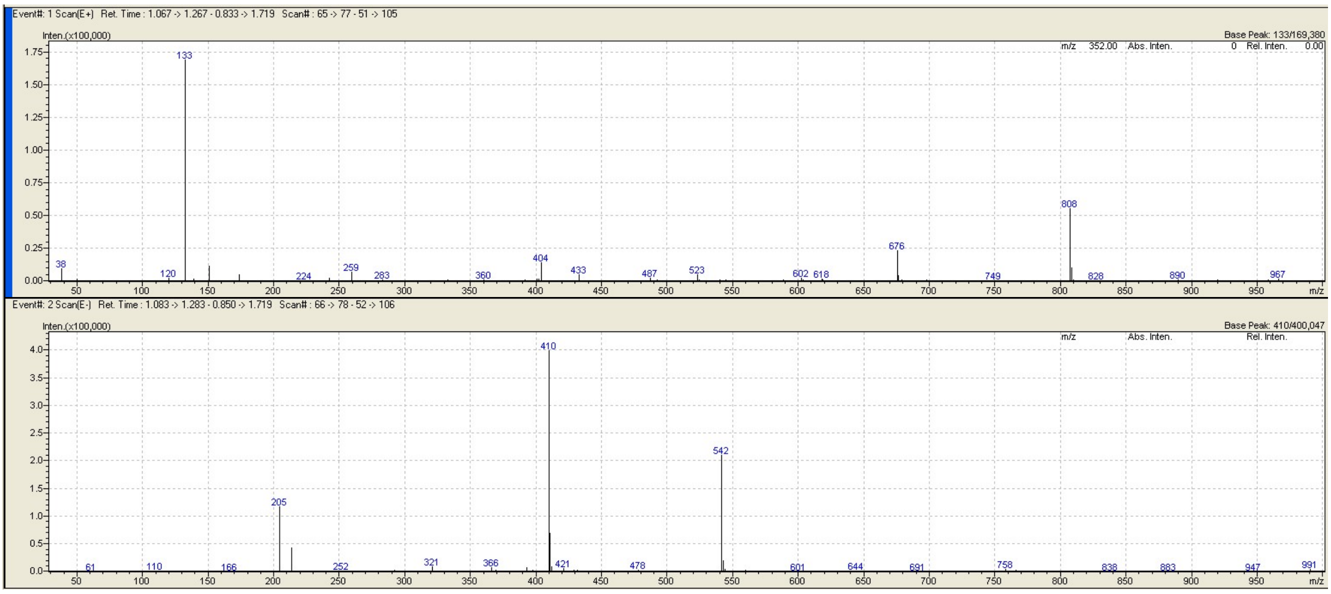

Figure S19: ESI-MS spectra of **2**-Cs in water. Positive-ion mode (top), negative-ion mode (bottom).

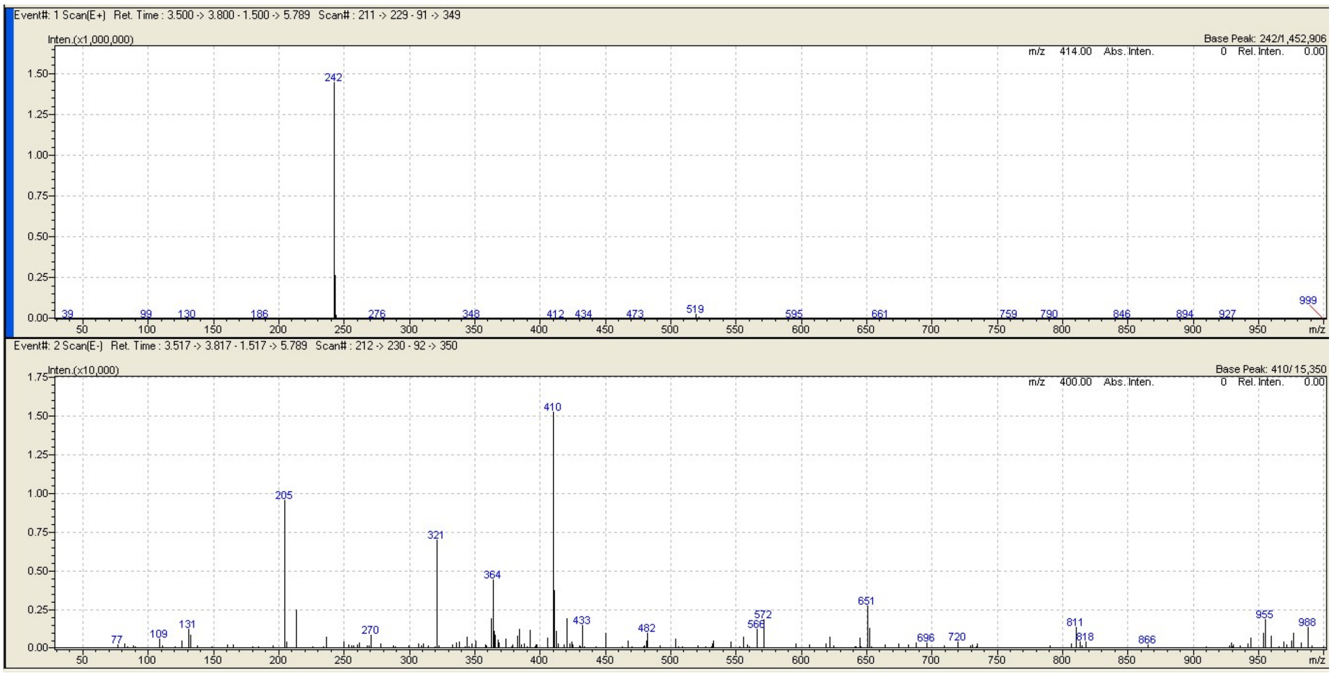

Figure S20: ESI-MS spectra of **2**-Bu_4N in water. Positive-ion mode (top), negative-ion mode (bottom).

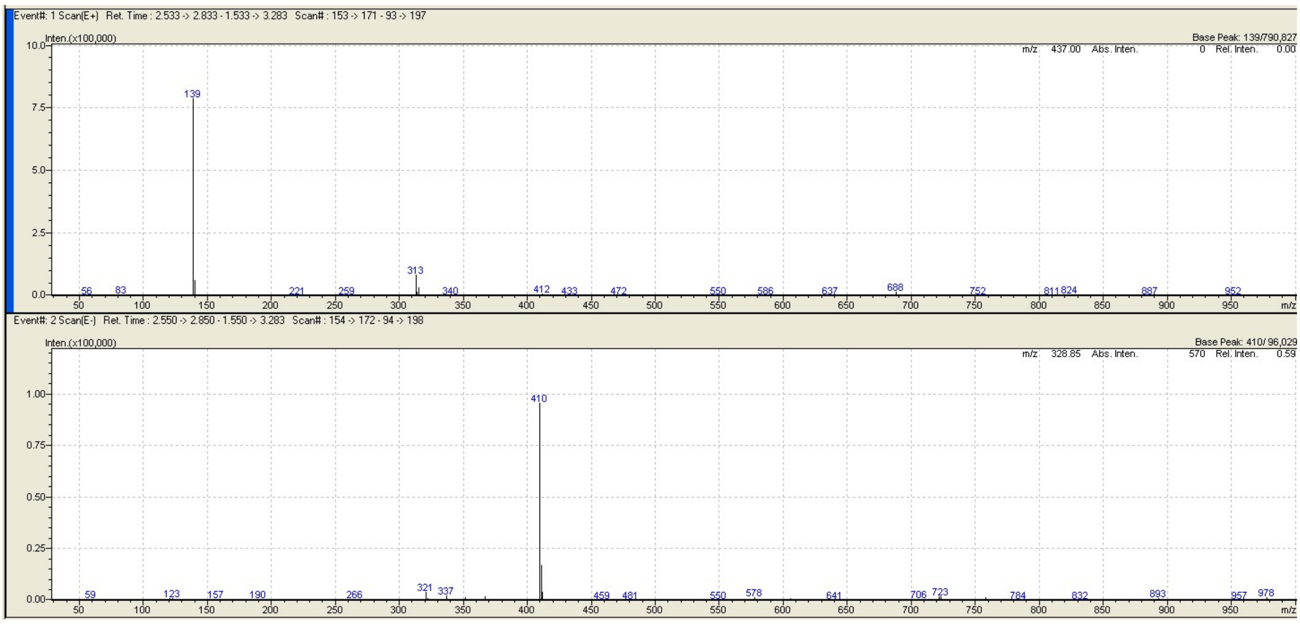

Figure S21: ESI-MS spectra of **2**-Bmim in water. Positive-ion mode (top), negative-ion mode (bottom).

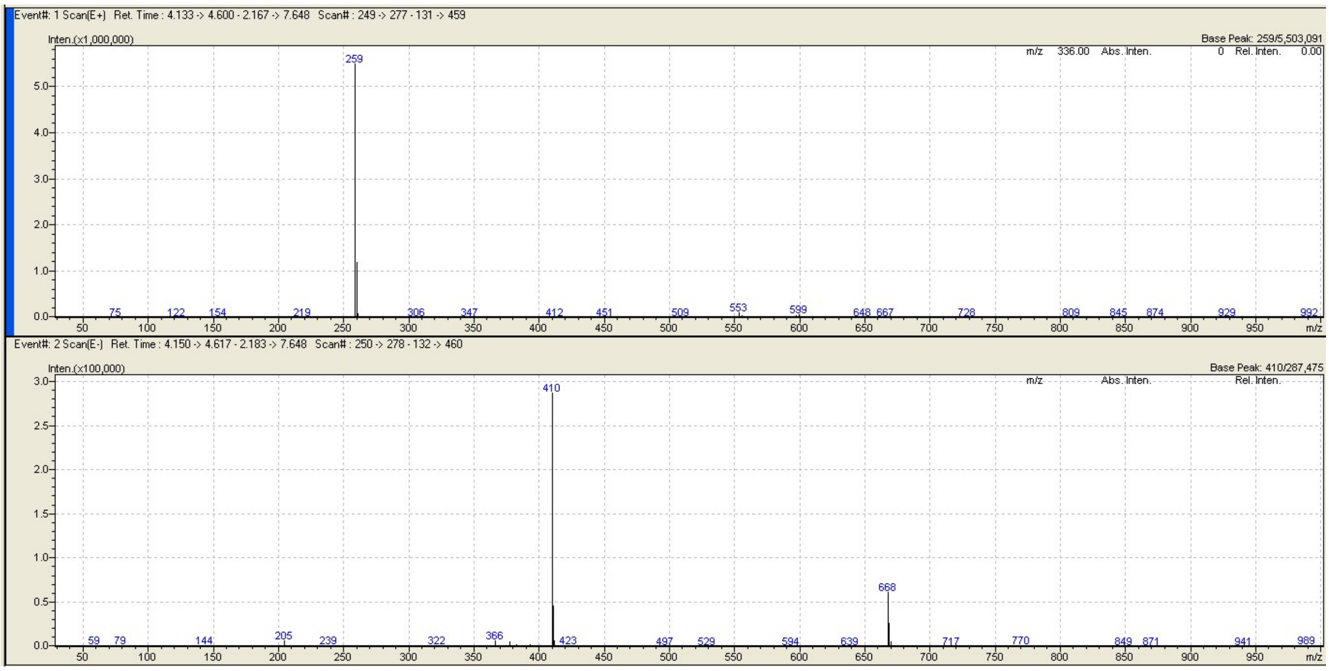

Figure S22: ESI-MS spectra of **2**-Bu_4P in water. Positive-ion mode (top), negative-ion mode (bottom).

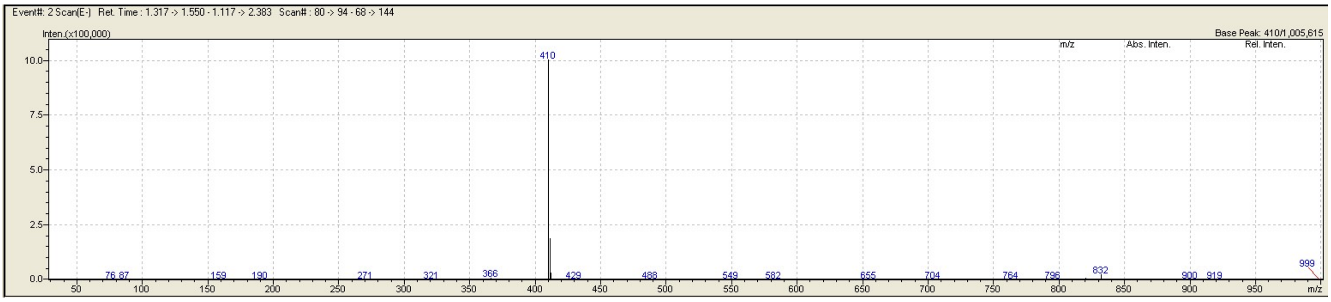

Figure S23: Negative-ion ESI-MS spectrum of **3**-Ba in water.

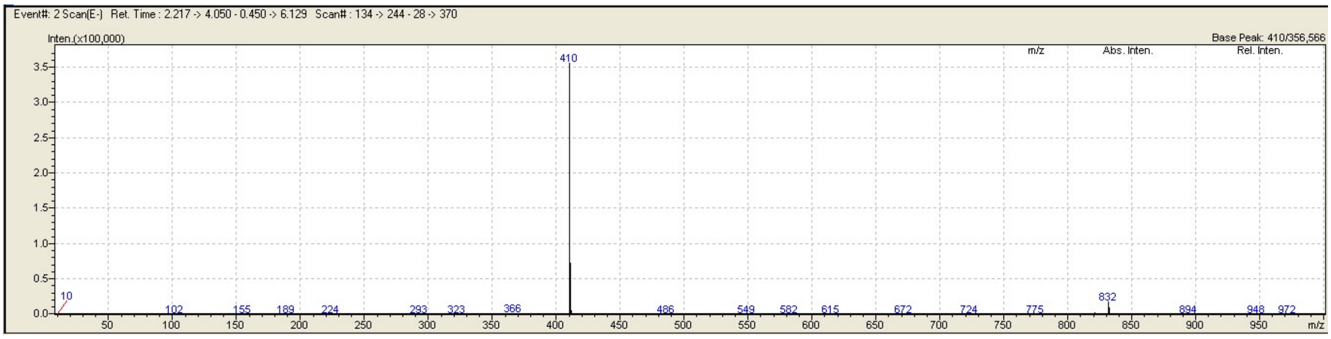

Figure S24: Negative-ion ESI-MS spectrum of **3**-Mg in water.

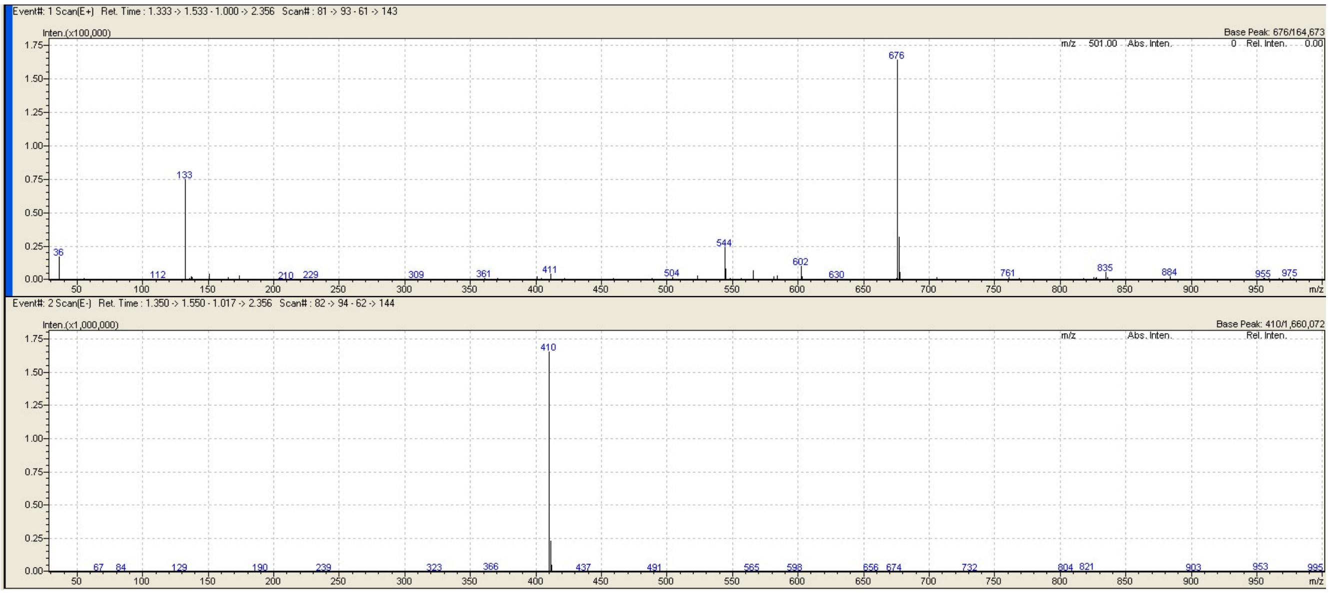

Figure S25: ESI-MS spectra of **3**-Cs in water. Positive-ion mode (top), negative-ion mode (bottom).

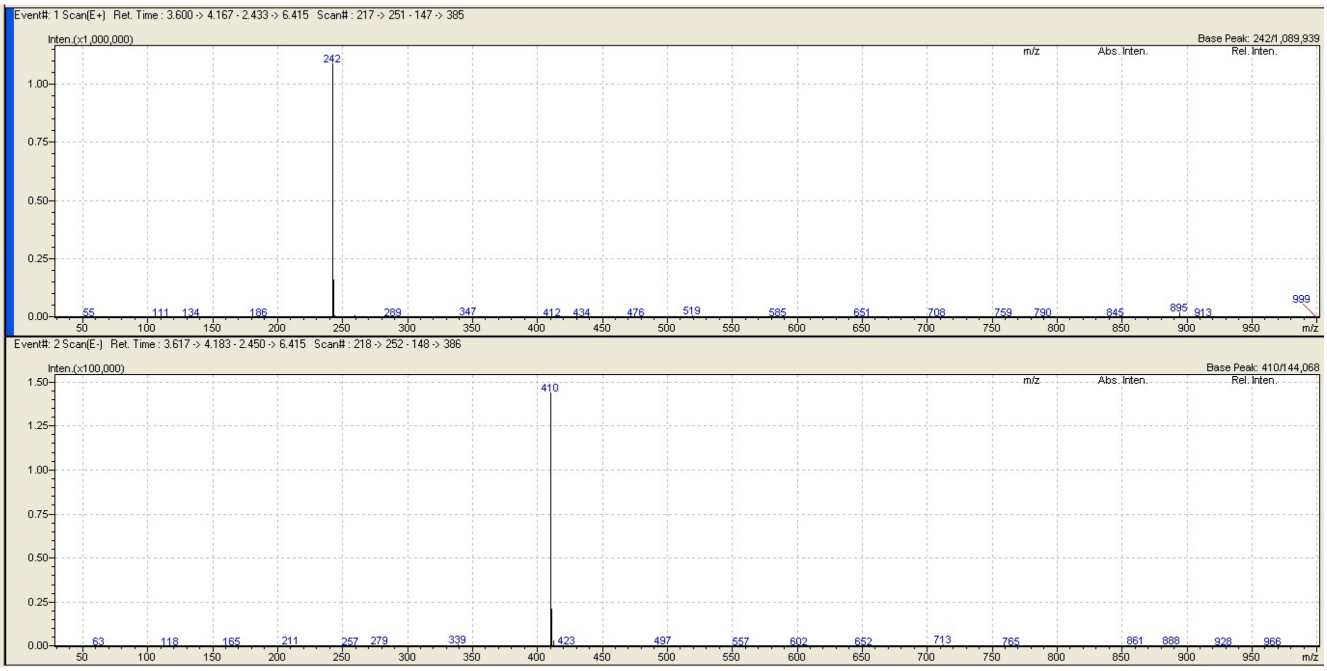

Figure S26: ESI-MS spectra of **3**-Bu_4N in water. Positive-ion mode (top), negative-ion mode (bottom).

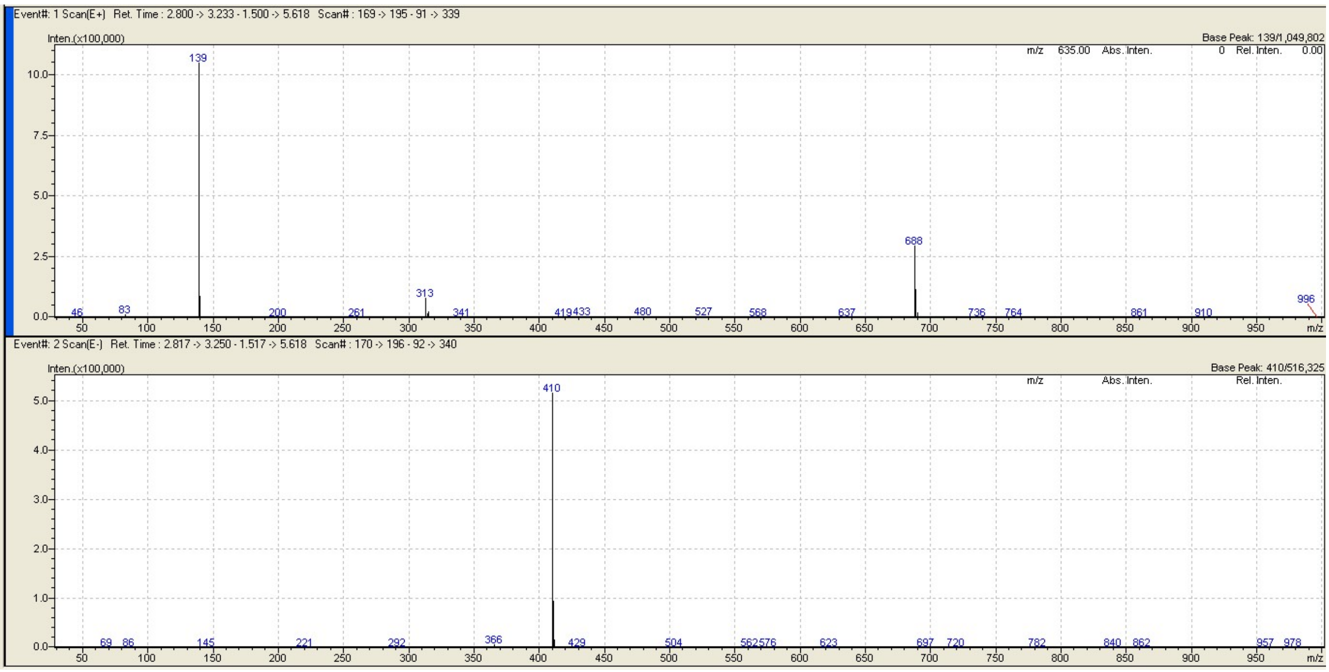

Figure S27: ESI-MS spectra of **3**-Bmim in water. Positive-ion mode (top), negative-ion mode (bottom).

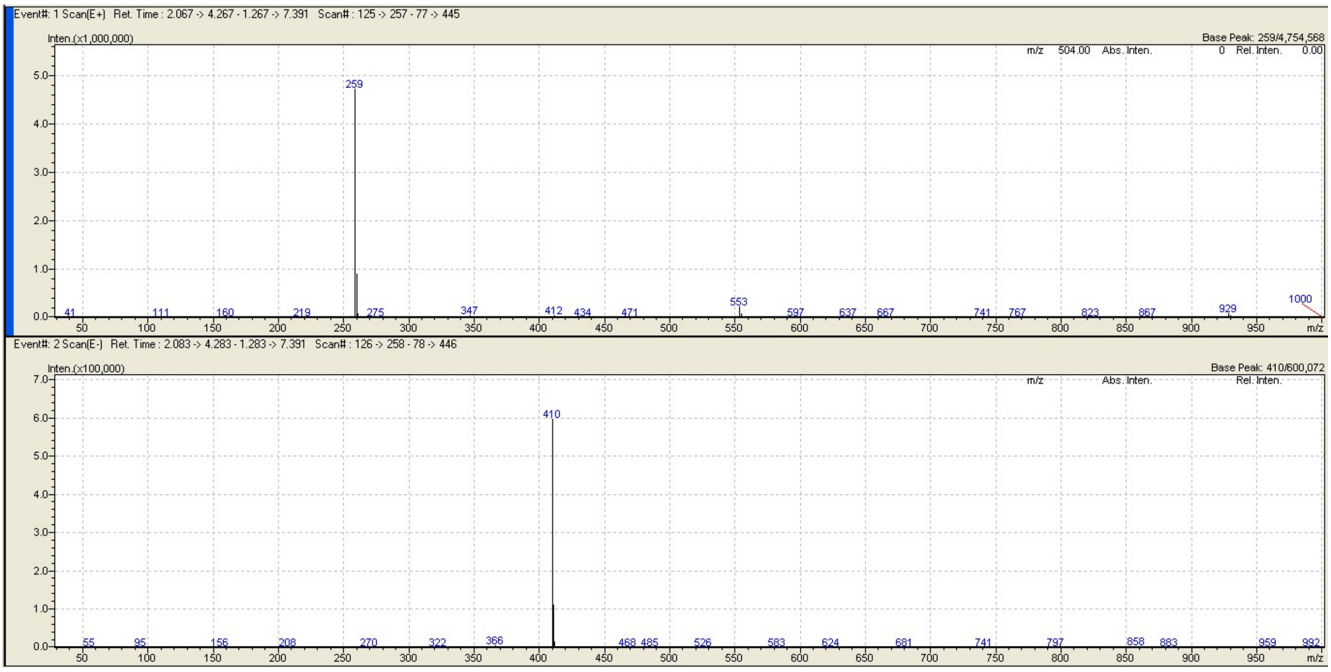

Figure S28: ESI-MS spectra of **3**-Bu_4P in water. Positive-ion mode (top), negative-ion mode (bottom).

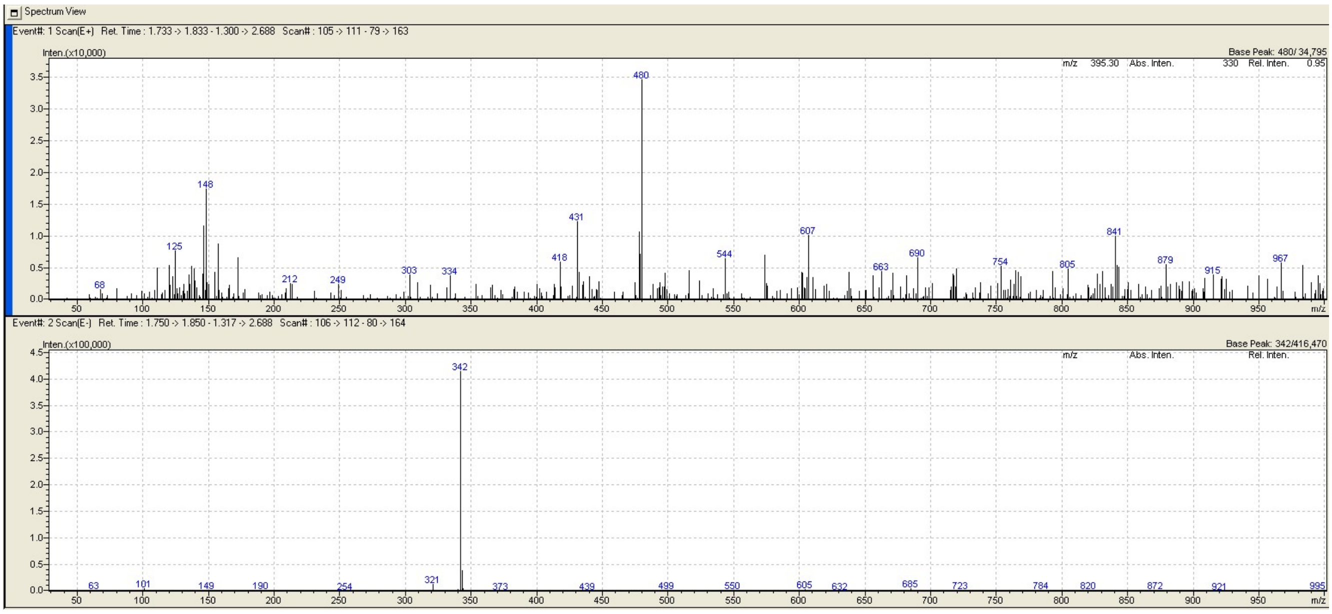

Figure S29: Negative-ion ESI-MS spectrum of **4**-Ba in water.

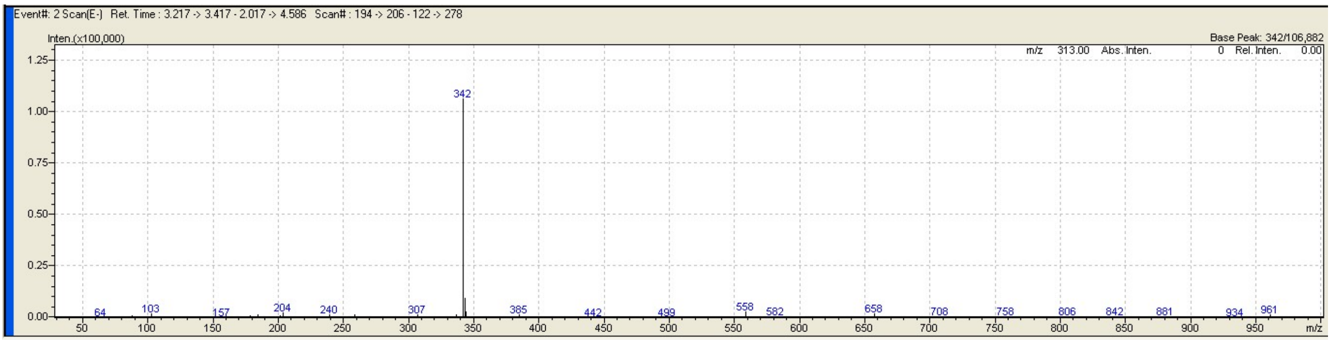

Figure S30: Negative-ion ESI-MS spectrum of **4**-Mg in water.

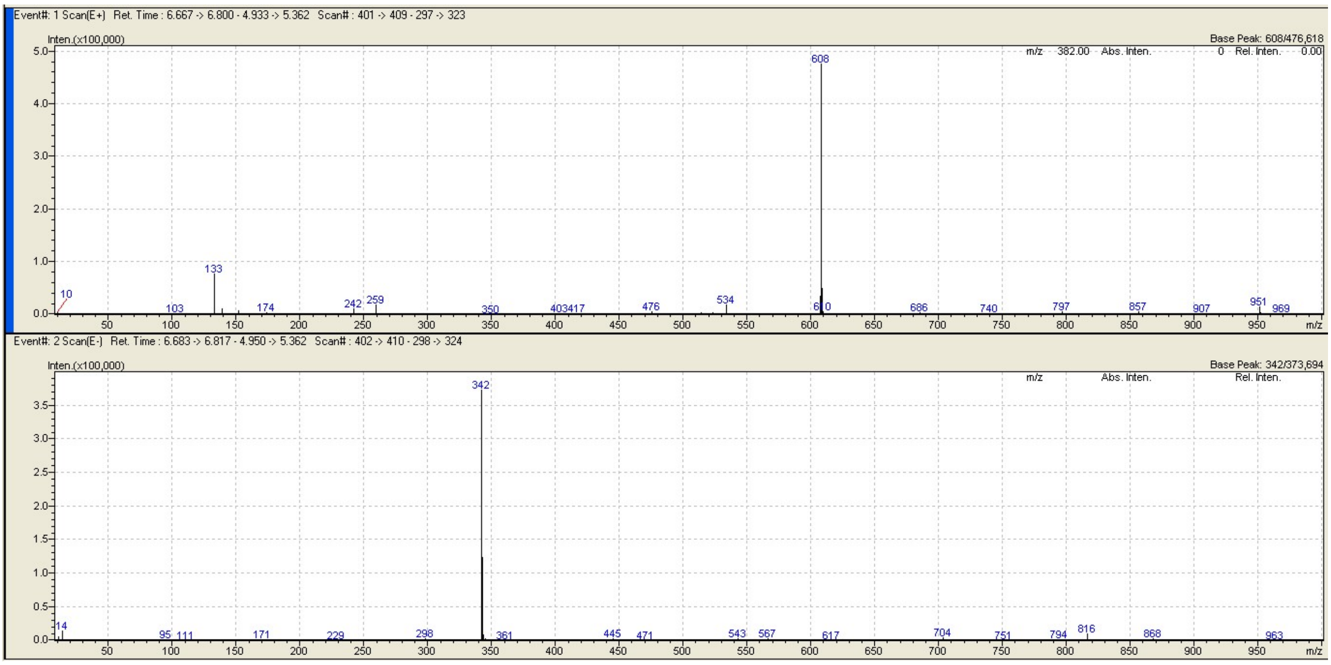

Figure S31: ESI-MS spectra of **4**-Cs in water. Positive-ion mode (top), negative-ion mode (bottom).

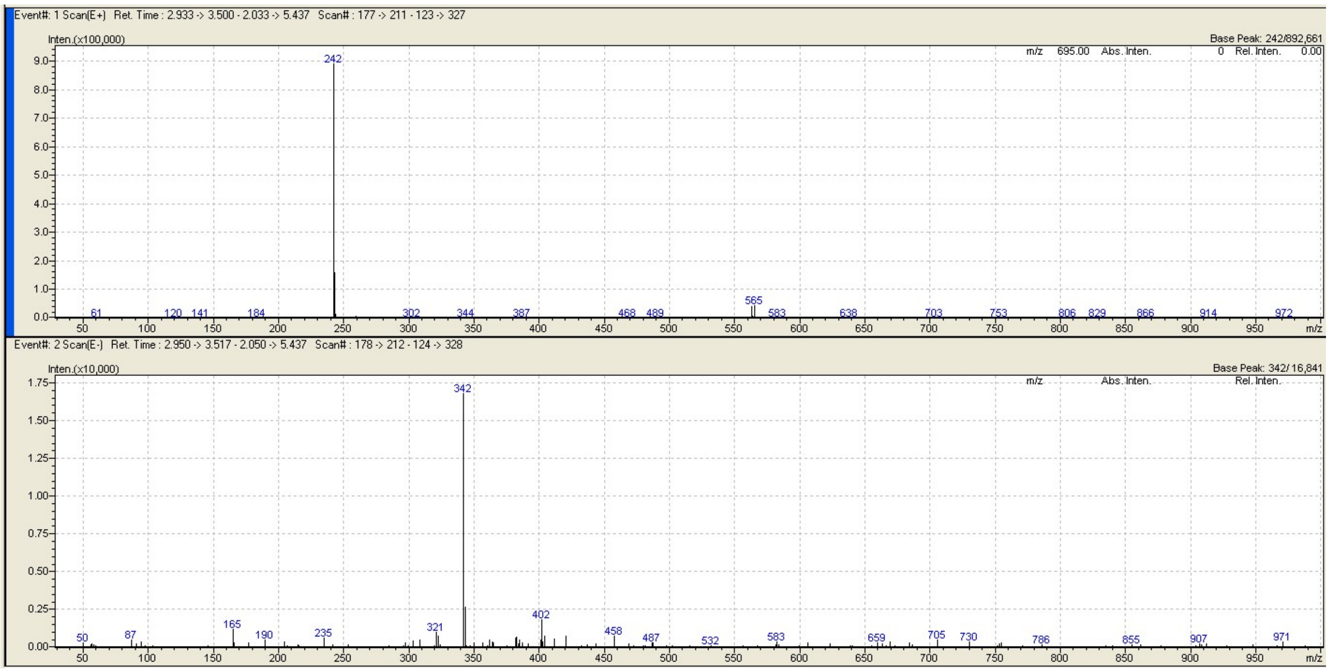

Figure S32: ESI-MS spectra of **4**-Bu_4N in water. Positive-ion mode (top), negative-ion mode (bottom).

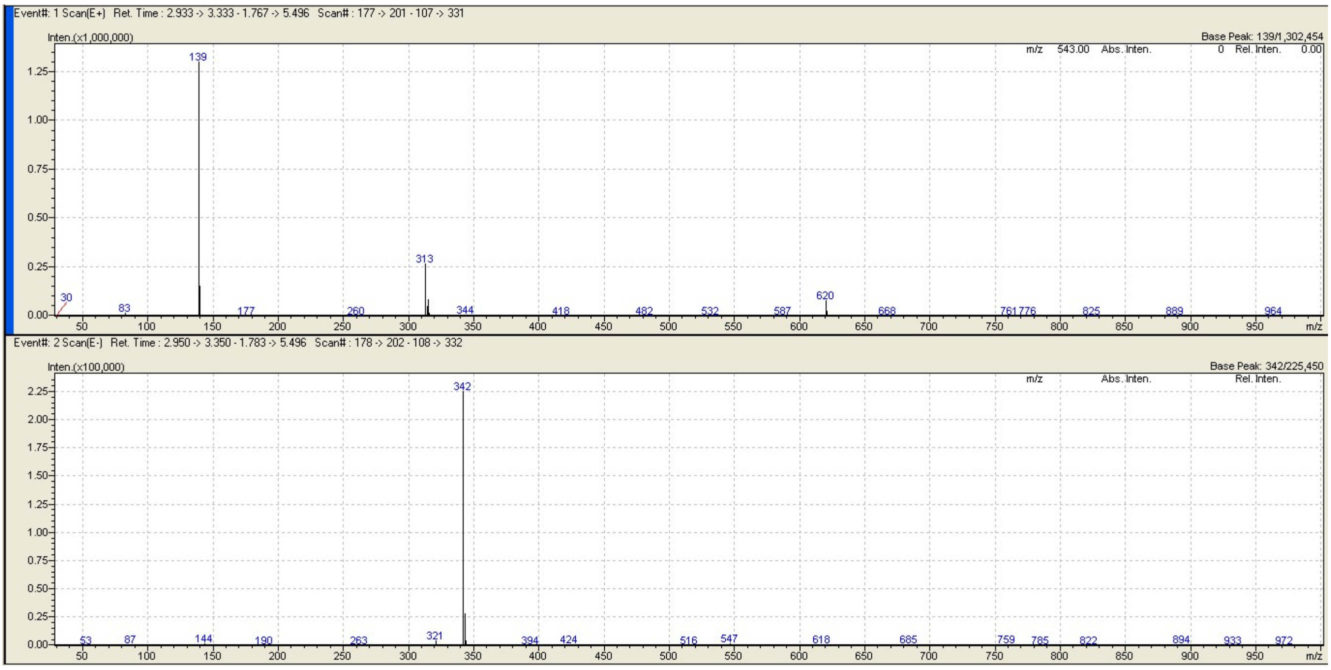

Figure S33: ESI-MS spectra of **4**-Bmim in water. Positive-ion mode (top), negative-ion mode (bottom).

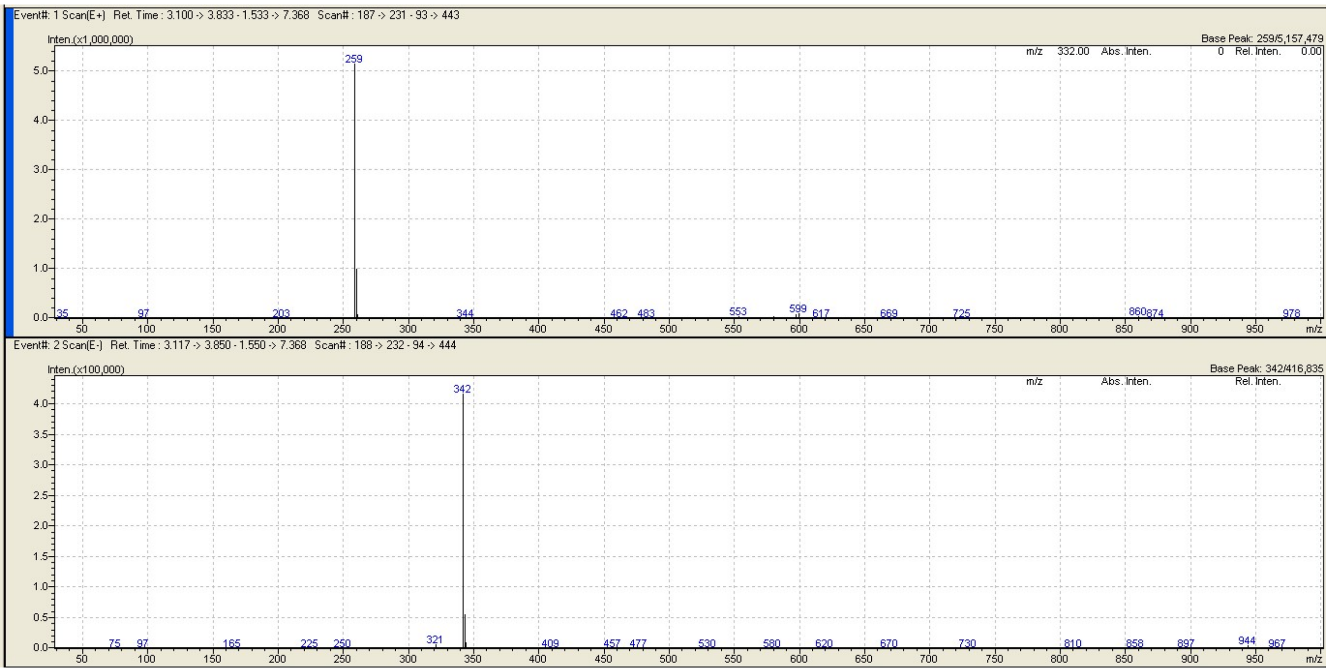

Figure S34: ESI-MS spectra of **4**-Bu_4P in water. Positive-ion mode (top), negative-ion mode (bottom).

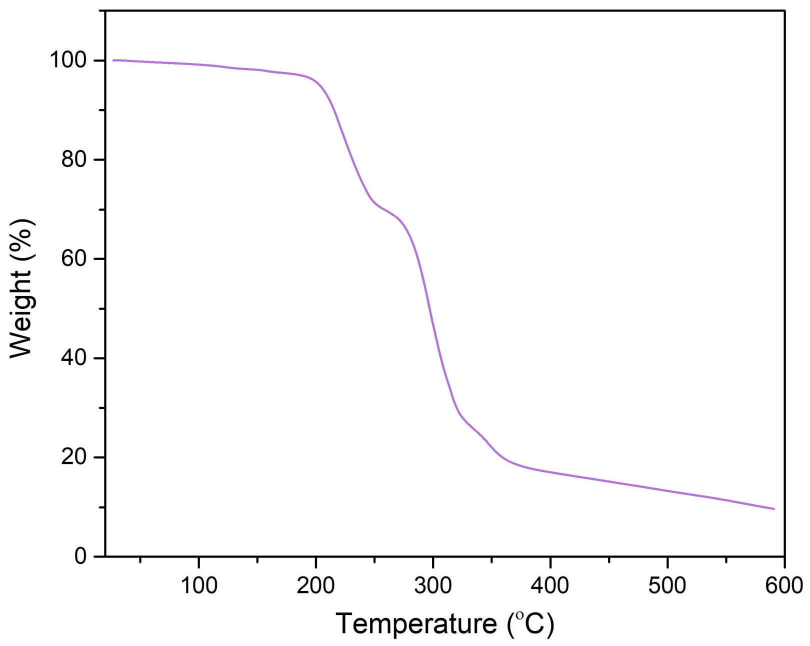

Figure S35: TGA results of **1**-Bu_4N.

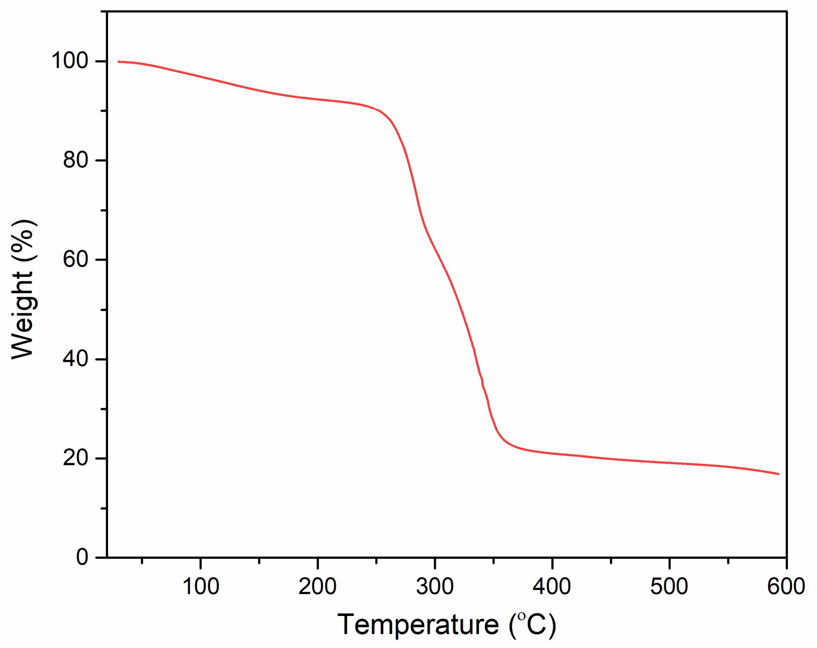

Figure S36: TGA results of **1**-Bmim.

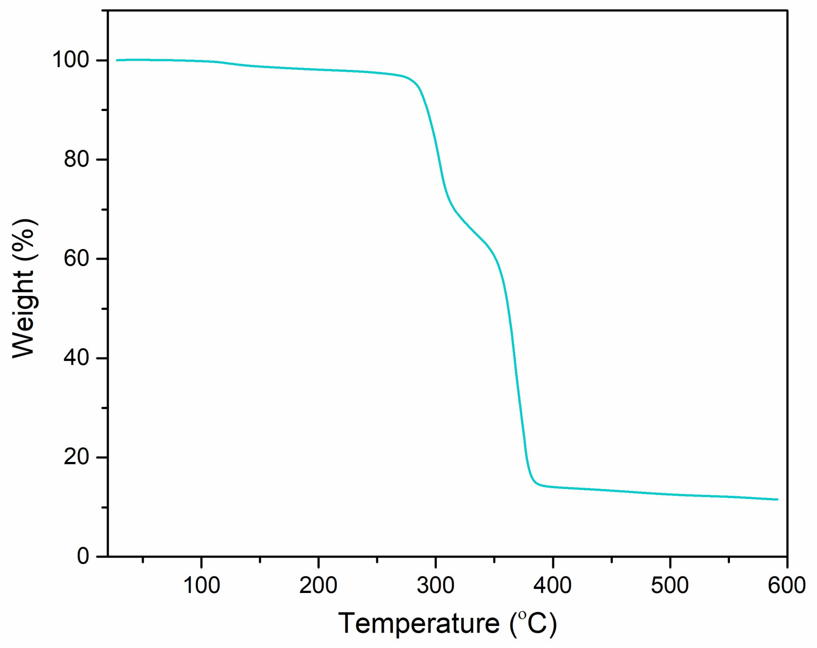

Figure S37: TGA results of **1**-Bu_4P.

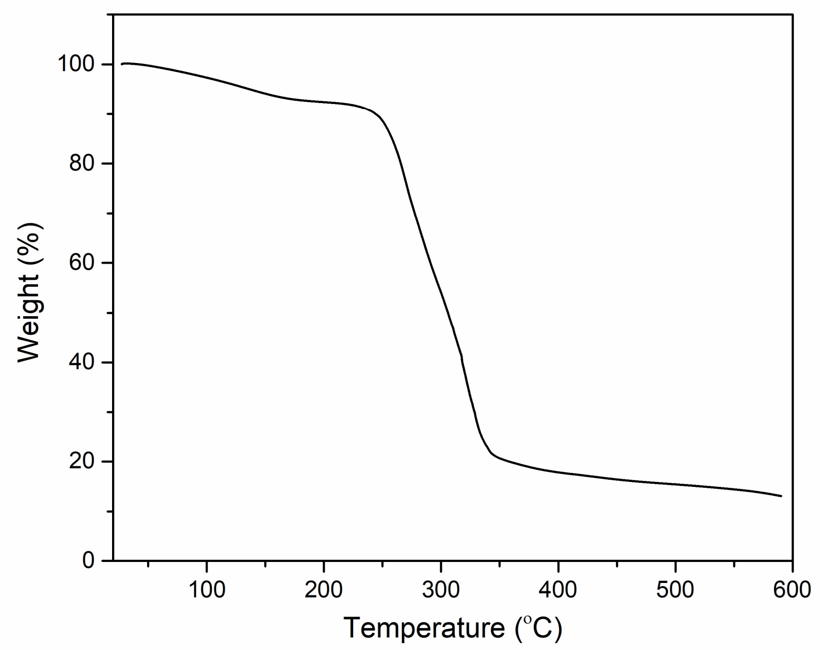

Figure S38: TGA results of **2**-Bmim.

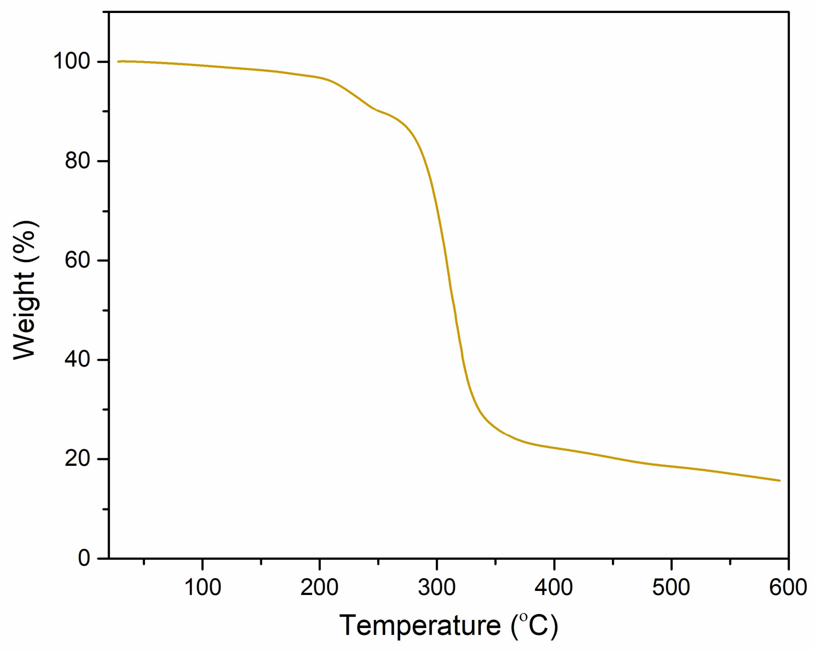

Figure S39: TGA results of **3**-Bu_4N.

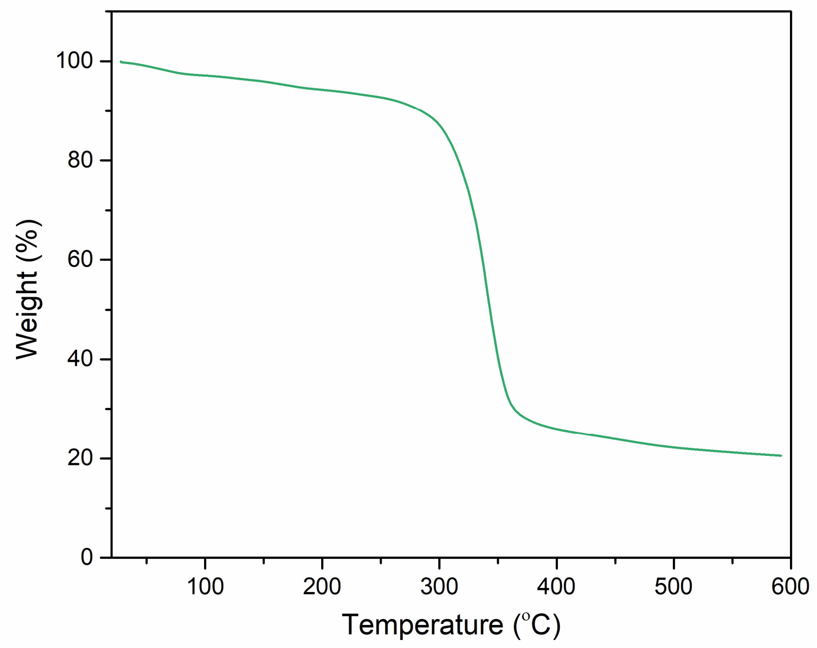

Figure S40: TGA results of **3**-Bmim.

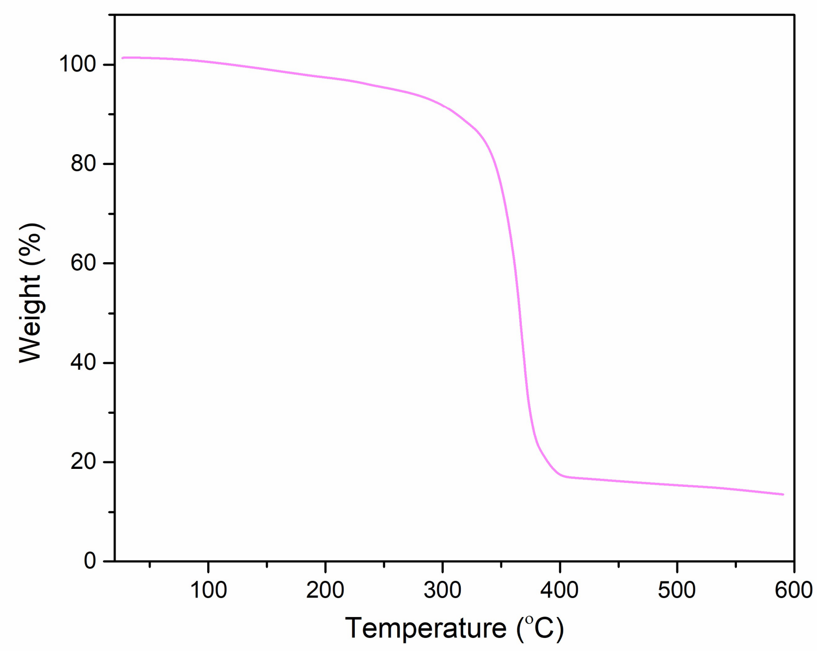

Figure S41: TGA results of **3**-Bu_4P.

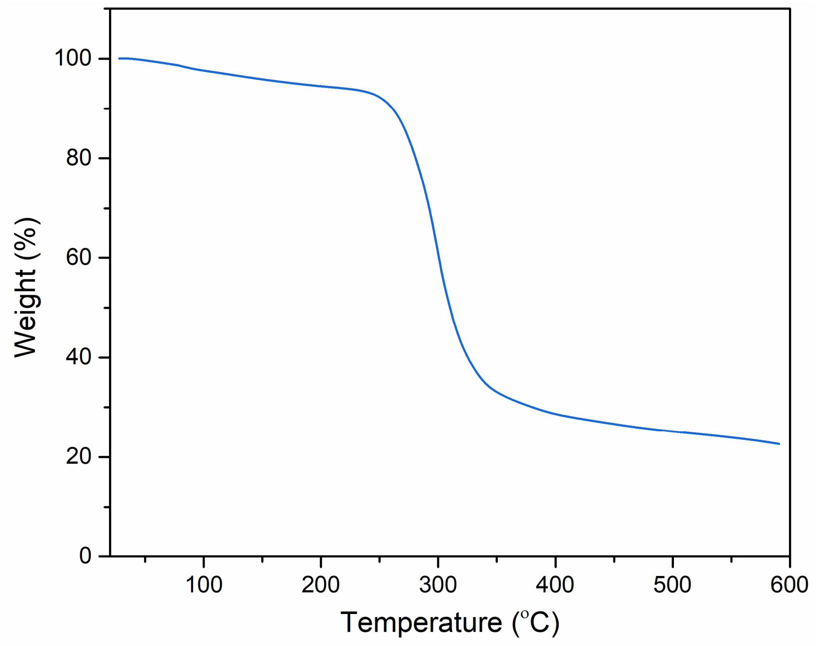

Figure S42: TGA results of **4**-Bmim.

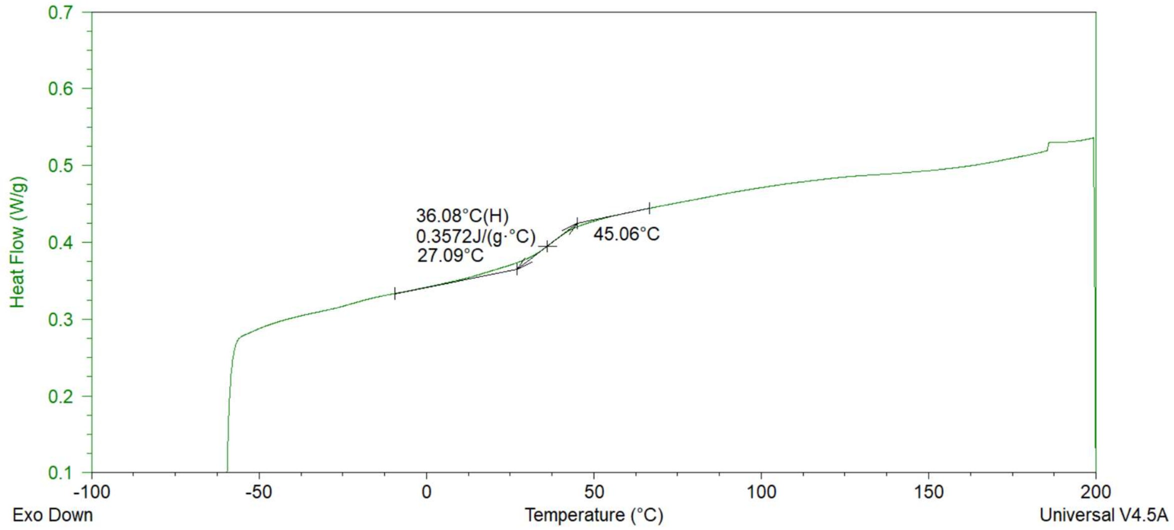

Figure S43: DSC analysis. Second heating run of **1**-Bu_4N.

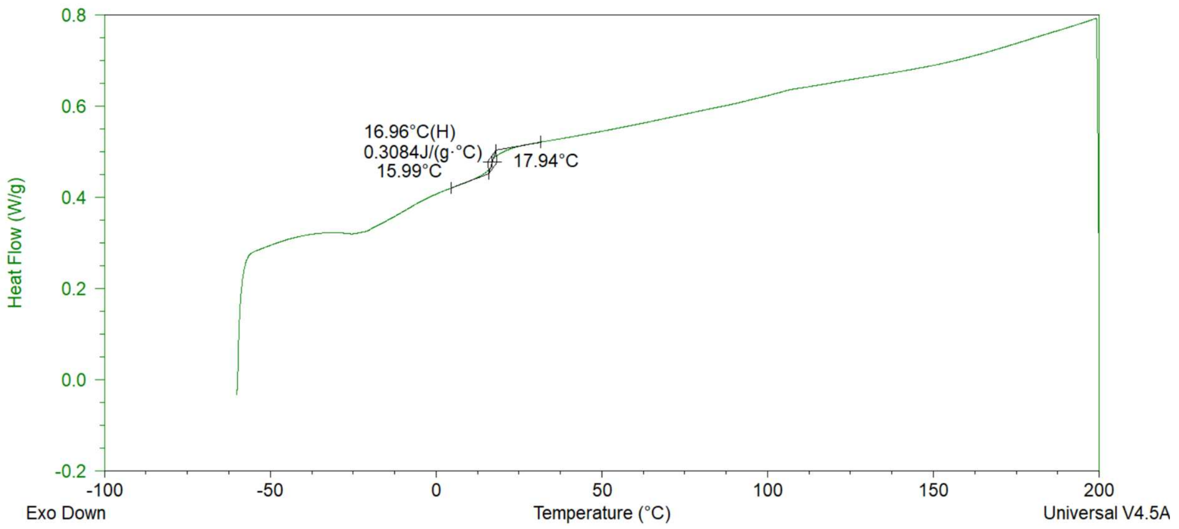

Figure S44: DSC analysis. Second heating run of **1**-Bmim.

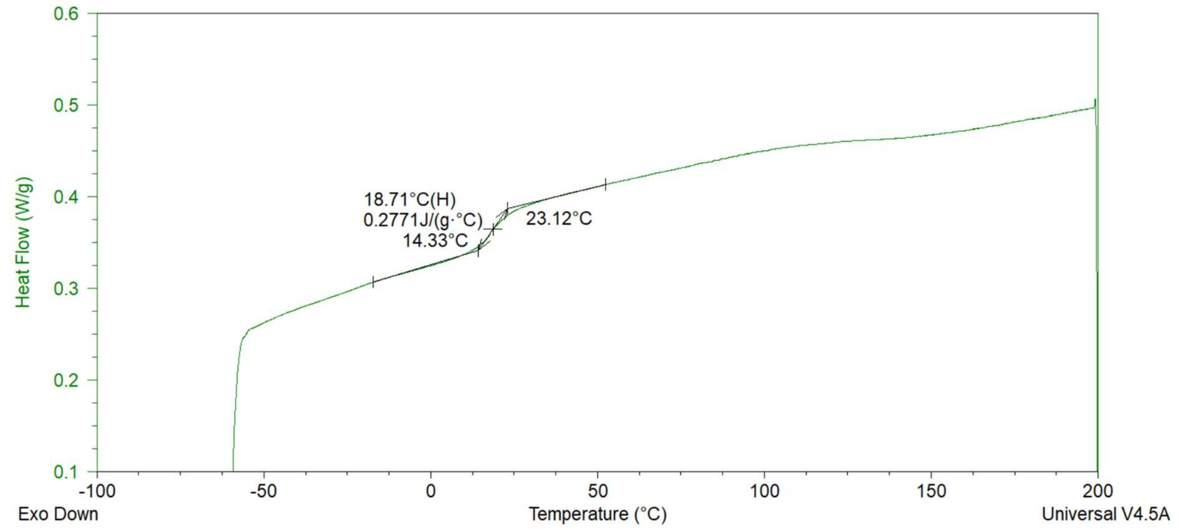

Figure S45: DSC analysis. Second heating run of **1**-Bu_4P.

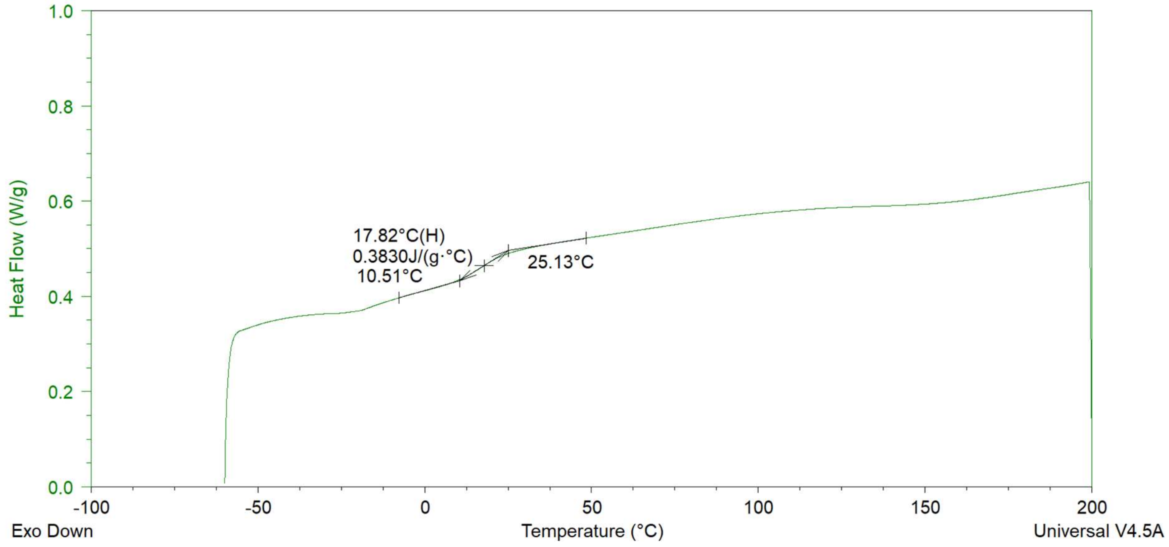

Figure S46: DSC analysis. Second heating run of **2**-Bmim.

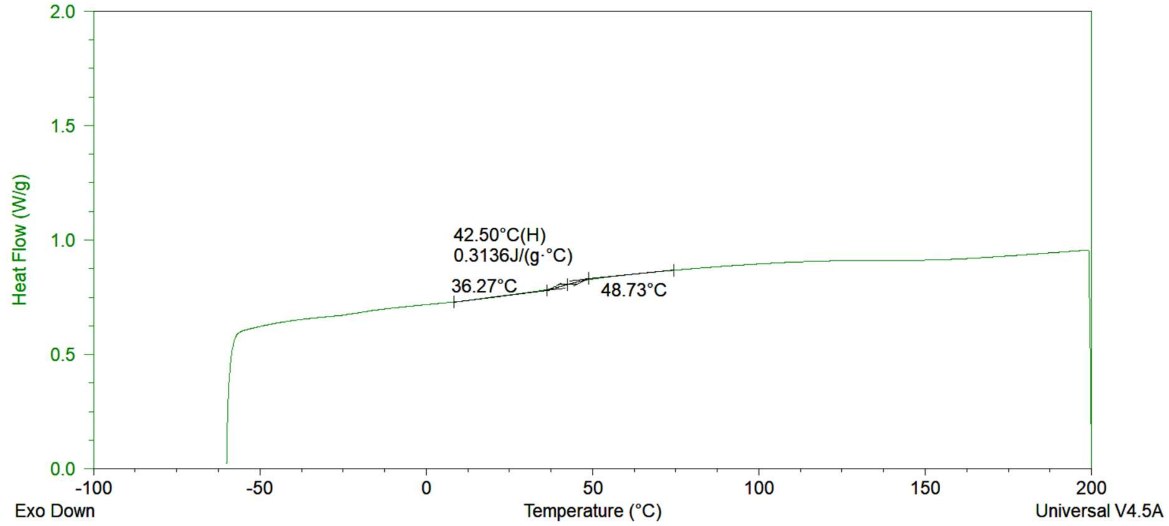

Figure S47: DSC analysis. Second heating run of **3**-Bu_4N.

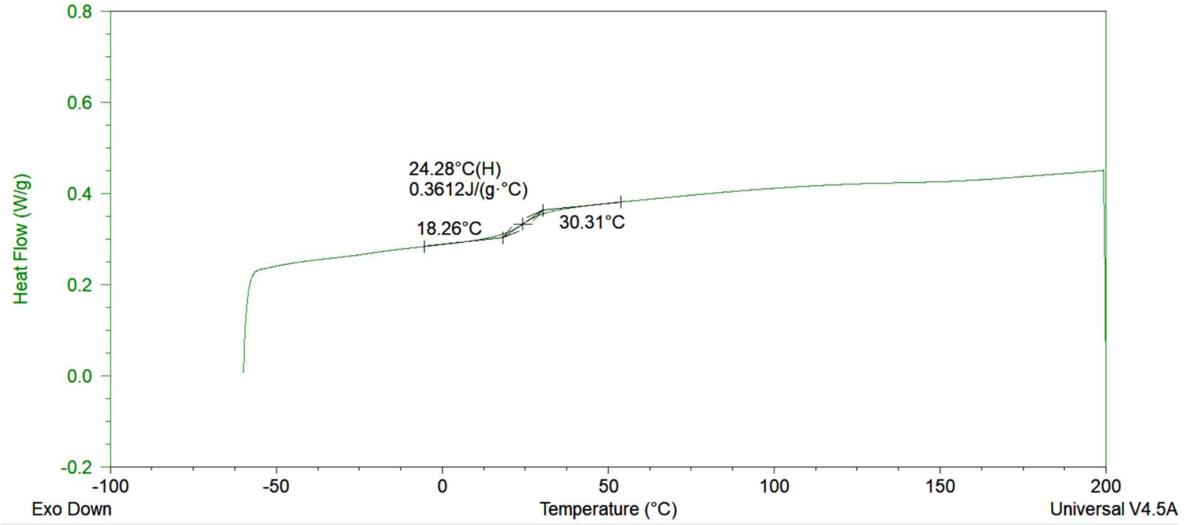

Figure S48: DSC analysis. Second heating run of **3**-Bmim.

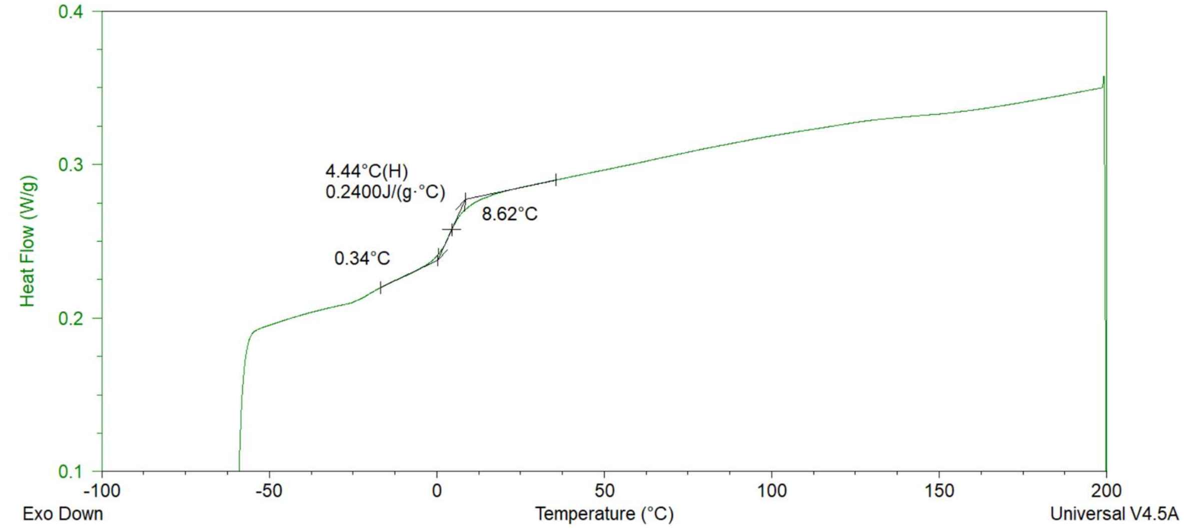

Figure S49: DSC analysis. Second heating run of **3**-Bu_4P.

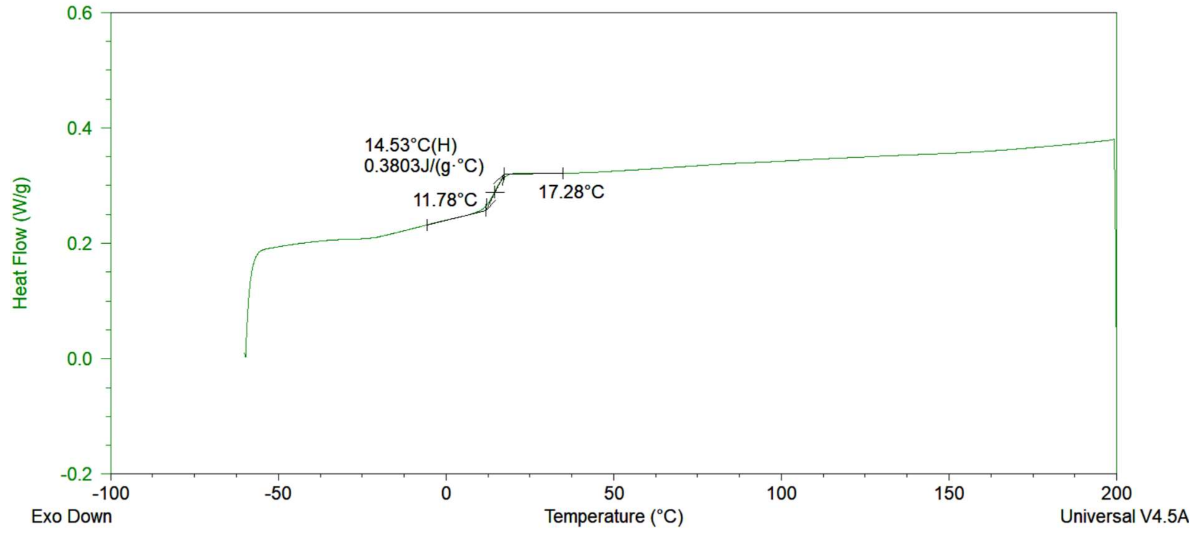

Figure S50: DSC analysis. Second heating run of **4**-Bmim.
